# Large Macrocyclic Lactones as Specific Cuticular Lipids of *Eueides* Butterflies

**DOI:** 10.1021/acs.jnatprod.6c00128

**Published:** 2026-03-24

**Authors:** Ozan Mehmet Solak, Stephanie Ehlers, Bruna Cama, Kanchon Dasmahapatra, Chris D. Jiggins, Stefan Schulz

**Affiliations:** a Institute of Organic Chemistry, 26527 Technische Universität Braunschweig, Braunschweig 38106, Germany; b Department of Biology, 8748 University of York, Heslington, York YO10 5DD, United Kingdom; c Department of Zoology, 2152 University of Cambridge, Cambridge CB2 3EJ, United Kingdom

## Abstract

Linear long-chain hydrocarbons are common constituents of the outer cuticle of most insects. These cuticular hydrocarbons form the first barrier to the environment, preventing desiccation, but also play important roles in communication among many insects, transporting, for example, information on species, sex, or physiological state. *Eueides* is a genus of heliconiine butterflies, a group known for their complex mimicry systems. We report here the identification and synthesis of large macrocyclic lactones from the cuticles of five *Eueides* species, which largely replace the hydrocarbons. These lactones have chain lengths of 23–34 and ring sizes of 17–29. They occur in *Eueides* but not in other closely related genera such as *Heliconius*, *Dryas*, *Dryadula*, *Agraulis,* or *Dione*. Each species has a specific mixture of macrocyclic lactones. These unique bouquets may be used in species recognition, as visual cues may be unreliable in mimicry rings. We identified 43 different compounds using GC/MS, GC/IR, and enantioselective synthesis via ring-closing metathesis and Jacobsen hydrolytic kinetic resolution. The absolute configuration could not be determined because these lactones were not separable by gas chromatography on chiral phases.

## Graphical abstract

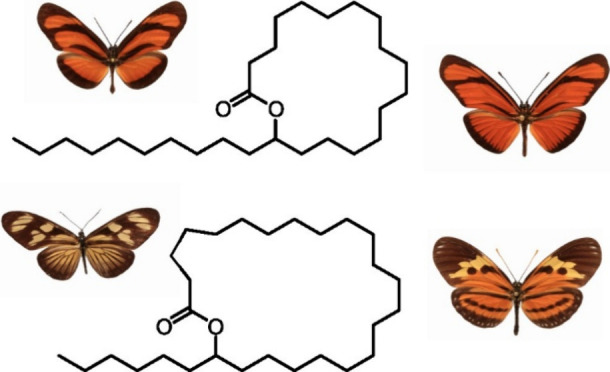

## Introduction

Insects use cuticular lipids as the outermost barrier against the environment. These lipids are found throughout the insect kingdom and consist predominantly of so-called cuticular hydrocarbons (CHCs), which are long alkanes with a chain length mostly between C_20_ and C_40_.^1^ They usually occur in mixtures and also often include methyl-branched alkanes. Additionally, alkenes and oxidized derivatives, such as aldehydes, ketones, alcohols, esters, or dialkyltetrahydrofurans, can be present in these mixtures. The overall bouquet can be complex with up to 200 compounds, and is often species-specific. The epicuticular layer helps prevent desiccation and often plays a role in chemical communication, such as indicating species, sex, colony identity, or physiological status.^1^

Butterflies are no exception. Their CHC mixtures consist mainly of *n*-alkanes and alkenes, but can additionally contain the other mentioned compound classes.^2–6^ Recently, we investigated the scent organs of *Heliconius* butterflies in detail,^7–12^ including androconia, which are scent or pheromone-releasing organs located on the wings of males. Androconia and the rest of the wing were analyzed separately to identify androconia-specific compounds. All of the 32 *Heliconius* species investigated contained CHCs as cuticular lipids; however, in the heliconiine genus *Eueides* , a different type of compounds occurred, not previously described. These are macrocyclic lactones with up to 33 carbons and ring sizes ranging from 17 to 29 (Chart 1), which are described in more detail in the following section.

**Chart 1:**
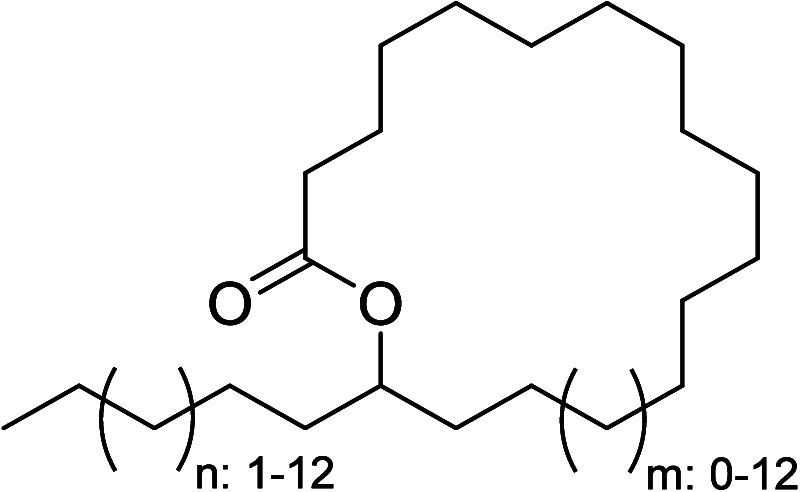
General Structure of Cuticular Macrocyclic Lactones of *Eueides* sp.

*Eueides* species are, in general, less abundant compared to many *Heliconius* species. Therefore, we report here the analyses of only a few individuals from five *Eueides* species available to us from previous studies.^8,10^ However, this material was sufficient to elucidate the structures of these large alkylated macrocyclic lactones, a new class of insect cuticular lipids, using GC/MS, GC/IR, and synthesis. We additionally analyzed individuals from butterfly collections, which was possible without destruction. The macrolides can be conveniently analyzed even in samples older than 20 years, showing their occurrence in species-specific mixtures.

## Results and Discussion

During the analysis of a wing extract of male *Eueides tales calathus* by GC/MS (Figure 1A), two major compounds, **1** and **2**, were detected that showed mass spectra (Figure 1B,C) that were different from those of known insect cuticular compounds. The mass spectra of both **1** and **2** showed a small M^+^ ion at *m*/*z* 408, and an intense ion at *m*/*z* 390, indicating loss of water (Figure 1). A second loss of water is revealed by a small ion at *m*/*z* 372. Together with ions at *m*/*z* 60 and 73, they indicate the presence of a lactone ring.^13^ This was supported by their IR spectra, which showed a strong C = O band at 1731 cm^–1^ and a weaker C–O band at 1176 cm^–1^ for both compounds, with no indication of an O–H or C = C band. High-resolution GC/Orbitrap-MS confirmed the formula of the M-18 ion for both compounds to be C_27_H_50_O (*m*/*z* 390.3858 and 390.3856, calcd. 390.3856 for C_27_H_50_O^+^), while the M^+^ ion was not visible in the Orbitrap spectrum (Figures S44 and S45). This indicated two double-bond equivalents. While these data were consistent with a saturated lactone, the ring size remained unclear.

**Figure 1:**
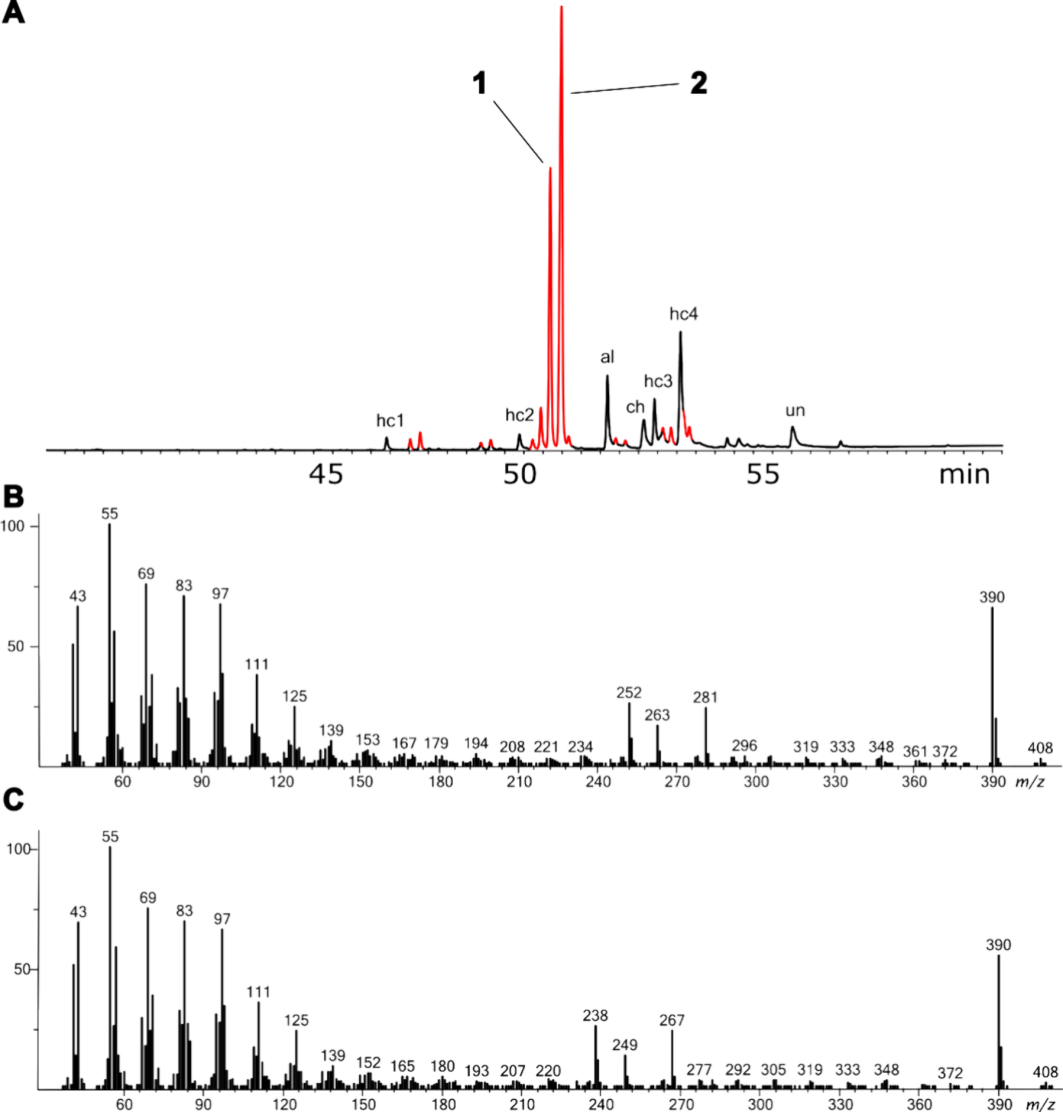
A: Total ion chromatogram (TIC) of an extract of the wings of male *Eueides tales*. Macrocyclic lactones are shown in red. The other compounds consist mainly of long-chain alkanes and aldehydes, with some unknown components. hc1: heptacosane; hc2: nonacosane; hc3: hentriacontane; hc4:9,21- and 9,23-dimethylhentriacontane; al: octacosanal; ch: cholesterol; un: unknown. B: Mass spectrum of natural compound **1** identified as heptacosan-18-olide. C: Mass spectrum of natural compound **2** identified as heptacosan-17-olide. The complete TIC is shown in Figure S47 in the SI.

However, as an example, the ions at *m*/*z* 238, 249, and 267 observed in the mass spectrum of **2**, allowed the determination of the ring size based on the known EI mass spectrometric behavior of macrocyclic lactones.^14^ Ion *m*/*z* 267 (**3**, C_17_H_31_O_2_, see Figure S45) can be formed by α-cleavage of the side chain (Figure 2). A subsequent loss of water leads to *m*/*z* 249 (C_17_H_29_O; see Figure S45). If the α-cleavage occurs inside the ring, a distonic radical cation **4** is formed that readily loses undecanal to form *m*/*z* 238 (**5**). This clarified compound **2** to be heptacosan-17-olide, while the ions *m*/*z* 253, 263, and 281 in **1** revealed similarly this compound to be heptacosan-18-olide. Using this approach, further macrolides were characterized in the extract, typically with slightly different ring sizes and chain length (Table 1). Ring size and chain length can be easily assigned by using the characteristic ions compiled in Table S2 in the SI. For example, next to **1** and **2**, heptacosan-16-, 19-, and 20-olides were also present in the *E. vibilia* extract. The gas chromatographic retention index *I* increases with the length of the side chain, from heptacosan-20-olide (*I* 2922) to heptacosan-17-olide (*I* 2965), thus leading to separation of the regioisomers.

**Table 1:** Occurrence of Macrocyclic Lactones in *Eueides* sp.^a^

	*E. vibilia*		*E. tales*			*E. lybia*	*E. aliphera*		*E. isabella*
	fw	cs	mw	cs	an	an^b^	fw	an	fw
tetracosan-16-olide	x								
tetracosan-17-olide	x		x						
tetracosan-18-olide	x		x						
pentacosan-16-olide	x								
pentacosan-17-olide	x		x		x				
pentacosan-18-olide	x		x		x				
pentacosan-19-olide	x								x
pentacosan-20-olide									x
hexacosan-17-olide	x		x		x				
hexacosan-18-olide	x		x		x				
hexacosan-19-olide	x	x	x						x
hexacosan-20-olide									x
hexacosan-21-olide									x
heptacosan-16-olide	x		x						
heptacosan-17-olide (**2**)	xx	x	xxx	xxx	xxx				
heptacosan-18-olide (**1**)	xxx	xxx	xxx	x	xx				
heptacosan-19-olide	xx	x	xx	x	x				
heptacosan-20-olide	x		xx	x	x	x			x
heptacosan-21-olide						x	xx	x	xx
heptacosan-22-olide						x	x	x	x
octacosan-17-olide	x		x		xx				
octacosan-18-olide (**9**)	xx		x		xx				
octacosan-19-olide	xx								
octacosan-20-olide									
octacosan-21-olide							x	x	xx
octacosan-22-olide (**11**)						x	xxx	xxx	xxx
octacosan-23-olide						x	xx	x	xx
nonacosan-17-olide			x		x				
nonacosan-18-olide	x		x		xx				
nonacosan-19-olide	x		x		xx				
nonacosan-20-olide	x		x		x			x	x
nonacosan-22-olide						x	x	x	xx
nonacosan-23-olide (**10**)						x	xxx	xxx	xx
nonacosan-24-olide (**13**)						x	xxx	xx	
triacontan-23-olide						x			
triacontan-24-olide (**12**)						x	xxx	xxx	
triacontan-25-olide (**14**)						x	x		
hentriacontan-24-olide						x	x		
hentriacontan-25-olide						x	x	x	
hentriacontan-26-olide						x			
tritriacontan-27-olide						x			
tritriacontan-28-olide						x			

a Whole wings in females were analyzed, while in males, androconia were dissected from the wings before analysis. Different body parts analyzed are indicated as follows: fw: female wings; mw: male wings; an: androconia; cs: male clasper scent glands. The relative abundance of a macrocyclic lactone compared to the major lactone in the extract is indicated: xxx: 20-100%, xx: 2-20%, x: < 2%.

b In this case, only traces of the macrocyclic lactones were detected; therefore, no xxx or xx were used.

**Figure 2:**
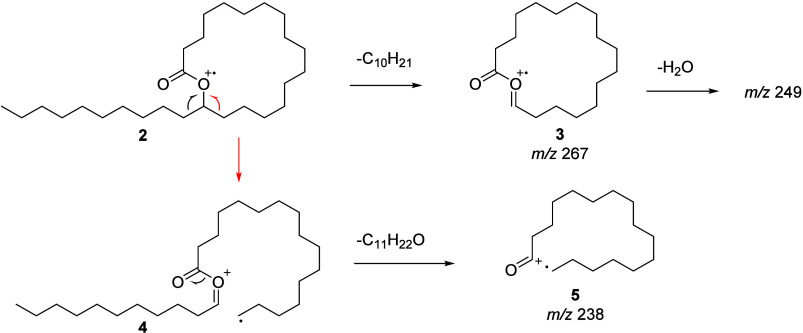
Mass spectrometric fragmentation of macrocyclic lactones, here heptacosan-17-olide (**2**), leading to ions *m*/*z* 238 (**5**), 267 (**3**), and 249, indicating the ring size.

To prove these structural proposals, four macrocyclic lactones were then synthesized according to Scheme 1. Due to the presence of a stereogenic center in the lactones, a stereoselective synthesis was planned. Commercially available oct-7-enyloxirane (**6**) was converted into its enantiomers using Jacobsen’s hydrolytic kinetic resolution; both epoxides, *R-* and *S-* **6**, were obtained with an ee >90%. Epoxide opening of *S* **-6** with nonylmagnesium bromide under CuI catalysis yielded nonadec-1-en-9-ol (*R* **-2**) that was cleanly esterified with dec-9-enoic acid to form ester *R* **-8**. A following ring-closing-metathesis closed the ring, leading to an *E*/*Z*-mixture of the unsaturated macrocyclic lactone. The use of the Stewart-Grubbs catalyst in toluene at 60 °C gave the best results compared with Grubbs’ first- and second-generation catalysts. Finally, hydrogenation delivered the target lactone (*R*)-heptacosan-17-olide (*R* **-2)**, which proved identical to natural compound **2** by MS, IR, and GC data. The *S*-enantiomer *S* **-2** was prepared analogously. However, attempts to separate the enantiomers of the lactones by GC for the determination of their absolute configuration were unsuccessful. Although different chiral GC phases were tried, no separation of the racemate was achieved.

**Scheme 1:**
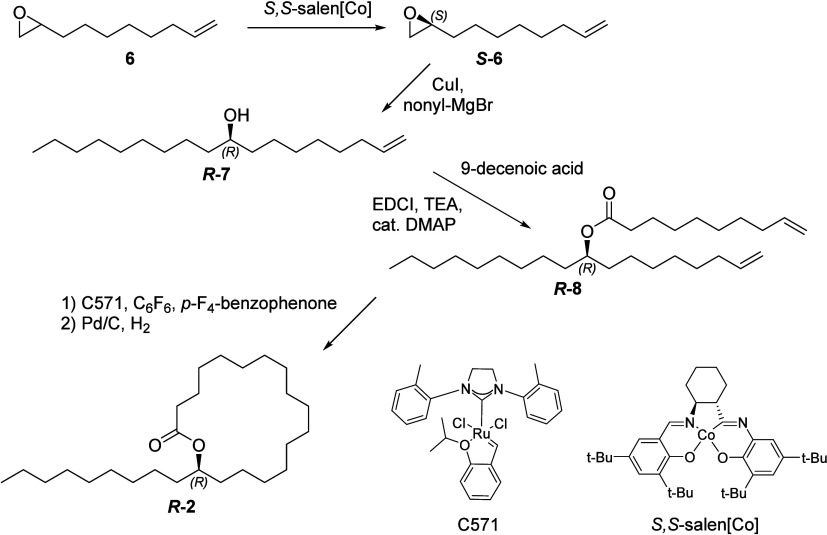
Synthesis of (*R*)-Heptacosan-17-olide (*R* **-2**)

Thus, the absolute configuration of natural **2** could not be established. However, this result was not surprising, as the large, flexible ring likely mimics an open alkyl chain around the stereogenic center. These compounds behave like achiral compounds with two almost identical alkyl groups at the stereogenic center. To further support the structural proposals, the lactones (*S*)-tricosan-17-olide and (*S*)-tetracosan-17-olide were prepared by the same route. The data of all four synthesized macrolides confirmed the proposed mass spectrometric fragmentation and the overall structure of the macrocyclic lactones.

Having established the structure of the lactones, we analyzed all available *Eueides* samples for their presence. Altogether, we identified 43 macrolides, ranging in ring size from 17 to 29, all of which exhibited characteristic ions indicative of their ring sizes in their mass spectra (Table 1 and Table S2). Some of the structures are shown in Chart 2. Although we had only a few samples available, it was evident that the macrolide composition differs between species. However, they occur in both females and males, unlike other butterfly signaling compounds, which are found preferentially in males.^15^ In *E. tales* samples from the wing, the clasper scent glands (CSGs), and the males’ specific, pheromone-dispersing androconia on the wings all showed a similar composition of the lactones, indicating their occurrence on the whole body. However, the CSGs contained lower amounts, likely because the samples contained less cuticular material than glandular material. In contrast, androconia showed a similar composition to the wings because they include a relatively large surface area, and showed no difference in composition from the other wing parts with respect to the macrocyclic lactones.

**Chart 2:**
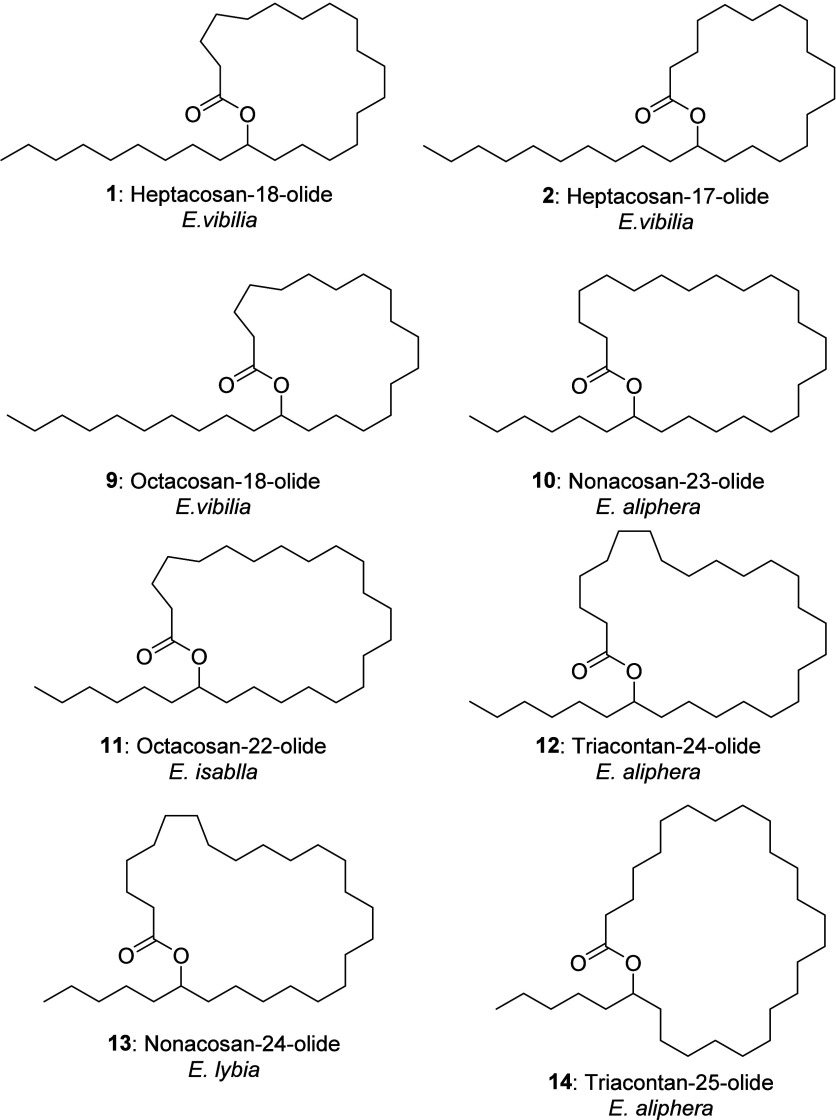
Structures of Some Major Constituents of *Eueides* Cuticular Lipids

The occurrence of the macrolactones seems to be species-specific. While **1** and **2** were the major components of *E. tales*, *E. vibilia* contained octacosan-18-olide (**9**) as a major component, together with various other lactones. *E. lybia* had only low amounts of quite large macrolides, e.g. triacontane-24-olide (**12**), while *E. aliphera* showed nonacosan-23-olide (**10**) as a major component, and octacosan-22-olide (**11**) occurred in *E. isabella*. In the last three species, only samples from the androconia were available.

Because of the limited number of recently collected samples, we tested whether dried specimens from older butterfly collections would allow us to analyze the occurrence and distribution more broadly within *Eueides.* Cuticular hydrocarbons (CHCs) have been analyzed from museum specimens of flies,^16^ wasps,^17–19^ and others,^20^ even on individuals over 200 years old.^17^ The CHCs are quite stable and can thus survive longer storage time when dry. Solvent extracts can be obtained by simple hexane/pentane extraction, leaving the specimens physically intact. We therefore analyzed wings and, when available, bodies of males and females of five *Eueides* species using short-time extraction with pentane and performing GC/MS analyses on both sexes. The CSG and the androconia could not be analyzed separately because doing so would have destroyed the specimens in the collection. The results are summarized in Table 2. In general, macrolactones were reduced relative to CHCs but could still be analyzed, showing distinct mixtures.

**Table 2:** Occurrence of Macrocyclic Lactones in *Eueides* sp. from Scientific Collections^a^

compound	*E. vibilia*		*E. lybia*				*E. isabella*		*E. aliphera*				*E. tales*	
	fw	mw	mb	mw	fb	fw	fw	mw	mb	mw	fb	fw	mw	fw
pentacosan-19-olide			x										x	x
hexacosan-17-olide		x												
hexacosan-18-olide		x												
hexacosan-20-olide			x											
hexacosan-21-olide			x											
heptacosan-17-olide (**2**)	xx	xx											xxx	xxx
heptacosan-18-olide (**1**)	xxx	xxx											x	x
heptacosan-19-olide	x	xx											x	x
heptacosan-20-olide		x	x	x			x	x						
heptacosan-21-olide		x	xxx	x	xx	x	xx	xx	x	x	x	x		
heptacosan-22-olide			xx	x	x	x	x	x	x		x	x		
heptacosan-23-olide			xx		x									
octacosan-17-olide		x												
octacosan-18-olide (**9**)	xx	xxx												
octacosan-19-olide	x	xx												
octacosan-20-olide		x												
octacosan-21-olide		x	x				x	x		x				
octacosan-22-olide (**11**)		xx	x	x	x	x	xxx	xxx	x	x	x	x		
octacosan-23-olide		x	x	x	x	x	x	x	x	x	x	x		
nonacosan-17-olide													x	
nonacosan-18-olide													xx	x
nonacosan-19-olide		x											x	x
nonacosan-20-olide		xx											x	x
nonacosan-22-olide		xx	x		x	xx	x	x	x	x	x	x		
nonacosan-23-olide (**10**)		x	xxx	xxx	xxx	xxx	xx	xx	xxx	xxx	xxx	xxx		
nonacosan-24-olide (**13**)			xxx	xxx	xxx	xxx			xxx	xxx	xxx	xxx		
triacontan-23-olide				x		x			x	x	x	x		
triacontan-24-olide (**12**)				x		x			xx	xx	xx	xx		
triacontan-25-olide (**14**)				x		x			x	x	x	x		
hentriacontan-24-olide						x					x	x		
hentriacontan-25-olide				x		x			x	x	x	x		
Hentriacontan-26-olide			x	x	x									

a Different body parts analyzed are indicated as follows: fw: female wings; mw: male wings; fb: female body; mb: male body. Wings of males included androconia, and bodies included the clasper scent glands. The relative abundance of a macrocyclic lactone compared to the major lactone in the extract is indicated: xxx: 20-100%, xx: 2-20%, x: < 2%.

In general, differences between males and females were low, as was the case for wings and bodies. For example, nonacosan-23-olide (**10**) and nonacosan-24-olide (**13**) were the major components in both males and females of *E. lybia*, in both wings and bodies. Bodies generally contained a slightly more diverse array of compounds compared to wings. *E. aliphera* had nonacosanolides **10** and **13** as major components, but they were accompanied by triacontan-24-olide (**12**) and triacontan-25-olide (**14**), while in *E. lybia* the shorter heptacosanolides were also prominent. *E. isabella* had 22-octacosanolide (**11**) as a major component, while *E. atra* had 17-heptacosanolide (**2**), closely related to *E. vibilia*, which has 18-heptacosanolide (**1**) and 18-octacosanolide (**9**). The data matched those of the wild-caught butterflies. However, although the data clearly support the species specificity of the macrolide mixtures, a more thorough analysis of additional individuals would be needed to more firmly establish these results.

By use of the heliconiine phylogeny by Kozak et al.^21^ (Figure S48), we analyzed whether it reflected the bouquet similarity between the different species. The related *E. aliphera* and *E. lybia* showed high compositional similarity, as both show 23- and 24-nonacosanolides as major components. However, *E. tales* with **1** and **2** dominating was clearly different from *E. lybia*, although it is phylogenetically very close to it. These three species occur in a different clade than *E. isabella* and *E. vibilia.* Both species showed different macrolide compositions, with *E. isabella* favoring an oxidation of the chain around C-21/C-22, while in *E. víbilia* C-18 dominated, supporting phylogenetic differences.

We also reanalyzed data from previous GC/MS analyses of 32 species of the related heliconiines genera *Heliconius*, *Dryas*, *Dione*, *Dryadula,* and *Agraulis*,^8–10^ but in no case did we find any of the discussed macrocyclic lactones. Therefore, we conclude that the lactones are specific for *Eueides*. The function of the lactones is unclear; they may simply replace parts of the CHCs, perhaps evolved by genetic drift, and/or be part of a communicative system involving species recognition, a particularly important task in butterfly mimicry groups.^10,22–24^ That CHCs can play an important role in species discrimination has been shown in butterflies for *Papilio polytes* ^2^ and *Colias* sp.^25,26^

The described macrocyclic lactones, which contain a large ring and a long alkyl side chain, have not been reported in nature previously. Unbranched macrocyclic lactones with ring sizes up to 24, but no alkyl chain, are found in halictine bees, where they function as signaling compounds.^27–29^ Smaller macrocyclic lactones with alkyl side chains occur in other Heliconiine species in the CSGs, but they do not have more than 20 carbons, and the ring size is between 10 and 13.^15,30,31^

The biosynthesis of the macrocyclic lactones likely originates from very long fatty acids that are also precursors in CHC biosynthesis.^32^ These acids, usually with an even-numbered carbon chain, lose the carboxy carbon to form CHCs. If, instead, a midchain hydroxylation occurs, a subsequent lactonization would lead to the title compounds, and the carboxylic group would be retained. Such midchain oxidation products of CHCs or their biosynthetic precursors are known in Lepidoptera as long secondary alcohols. These alcohols have been observed, e.g., in *Manduca sexta*,^33^ *Pieris* sp.,^5^ and *Idea leuconoe*.^34^ Similar to the macrolactones, they occur as mixtures of closely related positional isomers, e.g., 4- to 11-heptacosanol in *Pieris rapae*, but always only as minor components compared to CHCs.^30^

## Conclusions

Careful analysis of GC/MS data revealed the presence of long-chain, internally cyclized, large macrocyclic lactones in the cuticular lipid layer of *Eueides* butterflies. The structures were confirmed by the synthesis of representative members of this compound class. The macrolides occur in specific mixtures in the five *Eueides* sp. and are not known from other natural sources. In total, 43 compounds were detected, which represent major specific components of the cuticular layer. These macrolides replace in part the usual CHCs of the insect cuticle and are specific for *Eueides*, as they do not occur in other genera of the heliconiines. Although enantiomers were synthesized, resolution by chiral chromatography was unsuccessful. The biological function may be similar to CHCs, e.g. desiccation protection, but involvement in other traits, such as species recognition, is also possible. To address these questions, the synthesis of a range of macrolide congeners is needed to allow testing naturally occurring mixtures, as well as access to these butterflies in a larger scale.

## Experimental Section

### General Experimental Procedures

Specific rotation [α]_*D*_^25^ values were determined with an MCP 150 (Anton Paar) polarimeter. Measurements were performed at 25 °C with a 589 nm wavelength and a 100 mm cell length. IR spectra were measured on a Tensor 27 FT-IR-spectrometer with a diamond ATR unit (Bruker). NMR spectra were recorded on an Avance III 300 (^1^H NMR: 300 MHz, ^13^C NMR: 75 MHz) and an AVNeo600 (^1^H NMR: 600 MHz, ^13^C NMR: 150 MHz) instrument. All NMR spectra were referenced to tetramethylsilane at 0.00 ppm. Mass spectra were recorded with a combination of an Agilent Technologies 5977B gas chromatograph connected to an Agilent Technologies 8860 Series MSD. An Exactive GC Orbitrap mass spectrometer (ThermoScientific) was used for high-resolution MS. The resolution was set to 60,000 (fwhm; instrument setting at 200 u). The mass range was 50–650 u, and two micro scans were averaged per data scan. Automated gain control (AGC target) was set to 1 × 10^6^, and the maximum inject time was set to “auto”. Auxiliary temperatures were set to 290 °C for transfer lines 1 and 2, and the temperature of the electron ionization source was set to 220 °C. EI was performed at 70 eV energy in positive mode. Helium (carrier gas) and nitrogen (for C-Trap) were passed through gas purification cartridges (Thermo Scientific, Bremen, Germany) to remove moisture and organic impurities. The column bleed ion at 207.03235 u was used as a lock mass for internal mass calibration of the data. Column chromatography: silica 60 (0.063–0.200 mm, 70–230 mesh ASTM).

### Mass Spectral Data

The mass spectrometric data from extracts of butterflies collected in the wild used in this publication were obtained by reanalyzing previously published GC/MS data. The origin and analytical procedures of these samples were described earlier, for *E. vibilia* (1 individual) and *E. tales* (3 individuals) by Ehlers et al.,^8^ for *E. lybia* (4 individuals), *E. aliphera* (3 individuals), and *E. isabella* (1 individual) by Cama et al.^10^

For the analysis of butterflies from collections, dried individuals (one male and one female each) stored in envelopes from the University of York collection were used. A sample list is provided in the SI (Table S1). Usually, wings and bodies were already separated, but stored in the same envelope. Bodies were available only from *E. lybia* and *E. aliphera*. Wings or bodies were transferred to 5 mL vials and soaked in 1 mL pentane for 5 min. The pentane was transferred to another vial, and the solvent was evaporated in a hood until the samples were dry. The residue was taken up in 30 μL of pentane, and 2 μL were injected into the GC/MS system.

#### General Synthetic Protocol 1 (GSP1) for the Hydrolytic Kinetic Resolution of Epoxides

*N*,*N*′-Bis­(3,5-di-*tert*-butylsalicyliden)-1,2-cyclohexanediaminocobalt­(II) (Jacobsen HKR catalyst, 0.5 mol %) was added to a solution of racemic epoxide (1 equiv), acetic acid (2 mol %), and water (0.55 mol %).^35^ The reaction mixture was stirred at room temperature for 24 h. The enantiomerically enriched epoxide was isolated directly from the solution by distillation (50 °C, 1.3 mbar).

##### (*S*)-2-(Oct-7-en-1-yl)­oxirane (*S*-**6**)

According to GSP1, (*S*,*S*)-Jacobsen HKR catalyst (302 mg, 0.5 mmol, 0.5 mol %) was added to a solution of racemic 2-(oct-7-en-1-yl)­oxirane (**6**, 15.425 g, 100 mmol, 1 equiv), acetic acid (0.12 mL, 2 mmol, 2 mol %) and water (1.00 mL, 55 mmol, 0.55 equiv). (*S*)-2-(Oct-7-en-1-yl)­oxirane ((*S*-**6**) was isolated as a colorless liquid (6.607 g, 42.8 mmol, 43%). [α]_*D*_^25^ = −7.7 (c = 0.57 g/100 mL, CHCl_3_); IR (neat) ν_max_ 3115, 3047, 2924, 2855, 1640, 1451, 1257, 993, 910, 836, 636 cm^–1^; ^1^H NMR (CDCl_3_, 300 MHz) δ (ppm) 5.80 (ddt, *J* = 16.9, 10.2, 6.7 Hz, 1H), 4.99 (ddt, *J* = 17.0, 2.0, 1.5 Hz, 1H), 4.93 (ddt, *J* = 10.2, 2.3, 1.2 Hz, 1H), 2.93–2.87 (m, 1H), 2.74 (dd, *J* = 5.1, 4.0 Hz, 1H), 2.46 (dd, *J* = 5.1, 2.7 Hz, 1H), 2.10–2.00 (m, 2H), 1.57–1.25 (m, 10H); ^13^C NMR (CDCl_3_, 75 MHz) δ (ppm) 139.0 (CH), 114.2 (CH_2_), 52.3 (CH), 47.0 (CH_2_), 33.7 (CH_2_), 32.4 (CH_2_), 29.2 (CH_2_), 28.9 (CH_2_), 28.7 (CH_2_), 25.9 (CH_2_); HREIMS *m*/*z* 121.1013 [M-CH_5_O]^+^ (calcd. for C_9_H_13_, 121.1011).

##### (*R*)-2-(Oct-7-en-1-yl)­oxirane (*R* **-6**)

According to GSP1, (*R*,*R*)-Jacobsen HKR catalyst (115 mg, 0.19 mmol, 0.5 mol %) was added to a solution of racemic 2-(oct-7-en-1-yl)­oxirane (**6**, 5.920 g, 38.37 mmol, 1 equiv), acetic acid (0.05 mL, 0.67 mmol, 2 mol %) and water (0.38 mL, 21.10 mmol, 0.55 equiv). (*R*)-2-(Oct-7-en-1-yl)­oxirane (*R* **-6**) was isolated as a colorless liquid (2.141 g, 13.87 mmol, 36%). [α]_*D*_^25^ = +7.5 (c = 0.52 g/100 mL, CHCl_3_); IR (neat) ν_max_ 3115, 3047, 2924, 2855, 1640, 1451, 1257, 993, 910, 836, 636 cm^–1^; ^1^H NMR (CDCl_3_, 300 MHz) δ (ppm) 5.80 (ddt, *J* = 16.9, 10.2, 6.7 Hz, 1H), 4.99 (ddt, *J* = 17.0, 2.0, 1.5 Hz, 1H), 4.93 (ddt, *J* = 10.2, 2.3, 1.2 Hz, 1H), 2.93–2.87 (m, 1H), 2.74 (dd, *J* = 5.1, 4.0 Hz, 1H), 2.46 (dd, *J* = 5.1, 2.7 Hz, 1H), 2.10–2.00 (m, 2H), 1.57–1.25 (m, 10H); ^13^C NMR (CDCl_3_, 75 MHz) δ (ppm) 139.0 (CH), 114.2 (CH_2_), 52.3 (CH), 47.0 (CH_2_), 33.7 (CH_2_), 32.4 (CH_2_), 29.2 (CH_2_), 28.9 (CH_2_), 28.7 (CH_2_), 25.9 (CH_2_); HREIMS *m*/*z* 121.1013 [M-CH_5_O]^+^ (calcd. for C_9_H_13_, 121.1011).

#### General Synthetic Protocol 2 (GSP2) for Copper Mediated Ring Opening of Epoxides

The Grignard reagent (2 equiv) was added to a suspension of copper iodide (1 equiv) in THF (THF, degassed) under an argon atmosphere at −78 °C, and the solution was stirred at this temperature for half an hour. Epoxide (1 equiv) was added to the solution at this temperature, and the solution was stirred for 18 h as it slowly warmed to room temperature.^36^ The reaction mixture was hydrolyzed by the addition of saturated ammonium chloride solution, followed by extraction with diethyl ether. The combined organic extracts were washed with brine and dried over magnesium sulfate. The solvent was removed under reduced pressure, and the secondary alcohol was isolated by column chromatography (5:1, pentane/diethyl ether).

##### (*R*)-Pentadec-14-en-7-ol

According to GSP2, *n*-pentylmagnesium bromide (3.24 mL, 2 m in Et_2_O, 6.48 mmol, 2 equiv) was added to a suspension of copper iodide (617 mg, 3.24 mmol, 1 equiv) in THF (50 mL). (*S*)-2-(Oct-7-en-1-yl)­oxirane (*S* **-6**, 0.50 g, 3.24 mmol, 1 equiv) was added to the solution. (*R*)-Pentadec-14-en-7-ol was obtained as a colorless solid (0.63 g, 2.78 mmol, 86%). IR (neat) ν_max_ 3324, 2920, 2853, 1640, 1456, 1351, 1123, 1054, 992, 909, 853, 721, 627 cm^–1^; ^1^H NMR (CDCl_3_, 300 MHz) δ (ppm) 5.81 (ddt, *J* = 16.9, 10.2, 6.7 Hz, 1H), 5.03–4.95 (m, 2H), 4.95–4.90 (m, 1H), 3.62–3.54 (m, 1H), 2.09–2.00 (m, 2H), 1.50–1.26 (m, 20H), 0.92–0.86 (m, 3H); ^13^C NMR (CDCl_3_, 75 MHz) δ (ppm) 139.1 (CH), 114.2 (CH_2_), 72.0 (CH), 37.5 (CH_2_), 37.4 (CH_2_), 33.8 (CH_2_), 31.8 (CH_2_), 29.5 (CH_2_), 29.4 (CH_2_), 29.1 (CH_2_), 28.9 (CH_2_), 25.61 (CH_2_), 25.60 (CH_2_), 22.6 (CH_2_), 14.1 (CH_3_); EIMS *m*/*z* 208 ([M-H_2_O]^+^, 1), 141 (5), 123 (17), 97 (51), 81 (55), 67 (38), 55 (100), 41 (52); HREIMS *m*/*z* 208.2185 [M-H_2_O]^+^ (calcd. for C_15_H_28_ 208.2186).

##### (*R*)-Octadec-1-en-9-ol

According to GSP2, *n*-octylmagnesium bromide (6.48 mL, 2 m in Et_2_O, 12.96 mmol, 2 equiv) was added to a suspension of copper iodide (1.23 g, 6.48 mmol, 1 equiv) in THF (100 mL). (*S*)-2-(Oct-7-en-1-yl)­oxirane (*S* **-6**, 1.00 g, 6.48 mmol, 1 equiv) was added to the solution. (*R*)-Octadec-1-en-9-ol was obtained as a colorless solid (1.38 g, 5.15 mmol, 78%). IR (neat) ν_max_ 3324, 2917, 2849, 1641, 1460, 1350, 1117, 1034, 991, 909, 859, 721, 628 cm^–1^; ^1^H NMR (CDCl_3_, 300 MHz) δ (ppm) 5.81 (ddt, *J* = 16.9, 10.2, 6.7 Hz, 1H), 5.03–4.95 (m, 1H), 4.95–4.90 (m, 1H), 3.63–3.53 (m, 1H), 2.09–1.99 (m, 2H), 1.50–1.21 (m, 27H), 0.92–0.83 (m, 3H); ^13^C NMR (CDCl_3_, 75 MHz) δ (ppm) 139.1 (CH), 114.2 (CH_2_), 72.0 (CH), 37.5 (CH_2_), 37.4 (CH_2_), 33.8 (CH_2_), 31.9 (CH_2_), 29.7 (CH_2_), 29.6 (CH_2_), 29.56 (CH_2_), 29.53 (CH_2_), 29.3 (CH_2_), 29.1 (CH_2_), 28.9 (CH_2_), 25.7 (CH_2_), 25.6 (CH_2_), 22.7 (CH_2_), 14.1 (CH_3_); EIMS *m*/*z* 250 ([M-H_2_O]^+^, 4), 222 (3), 157 (15), 141 (8), 123 (43), 112 (13), 97 (48), 83 (97), 81 (100), 69 (60), 67 (59), 55 (91), 43 (42), 41 (55); HREIMS *m*/*z* 250.2657 [M-H_2_O]^+^ (calcd. for C_18_H_34_ 250.2655).

##### (*R*)-Nonadec-1-en-9-ol (*R* **-7**)

According to GSP2, *n*-nonylmagnesium bromide (12.97 mL, 1 m in Et_2_O, 12.96 mmol, 2 equiv) was added to a suspension of copper iodide (1.23 g, 6.48 mmol, 1 equiv) in THF (100 mL). (*S*)-2-(Oct-7-en-1-yl)­oxirane (*S* **-6**), 1.00 g, 6.48 mmol, 1 equiv) was added to the solution. (*R*)-Nonadec-1-en-9-ol (*R* **-7**) was obtained as a colorless solid (1.40 g, 4.95 mmol, 76%). IR (neat) ν_max_ 3324, 2917, 2849, 1641, 1460, 1350, 1117, 1034, 991, 909, 859, 721, 628 cm^–1^; ^1^H NMR (CDCl_3_, 300 MHz) δ (ppm) 5.81 (ddt, *J* = 17.1, 10.2, 6.6 Hz, 1H), 4.99 (ddt, *J* = 17.1, 2.1, 1.6 Hz, 1H), 4.93 (ddt, *J* = 10.2, 2.2, 1.2 Hz, 1H), 3.62–3.53 (m, 1H), 2.09–1.98 (m, 2H), 1.49–1.21 (m, 29H). 0.92–0.83 (m, 3H); ^13^C NMR (CDCl_3_, 75 MHz) δ (ppm) 139.1 (CH), 114.2 (CH_2_), 72.0 (CH), 37.49 (CH_2_), 37.45 (CH_2_), 38.8 (CH_2_), 31.9 (CH_2_), 29.7 (CH_2_), 29.63 (CH_2_), 29.61 (2C, CH_2_), 29.5 (CH_2_), 29.3 (CH_2_), 29.1 (CH_2_), 28.9 (CH_2_), 25.7 (CH_2_), 25.6 (CH_2_), 22.7 (CH_2_), 14.1 (CH_3_); EIMS *m*/*z* 264 ([M-H_2_O]^+^, 3), 171 (14), 141 (8), 123 (46), 111 (21), 97 (68), 83 (61), 81 (100), 69 (56), 67 (61), 57 (49), 55 (91), 43 (54), 41 (69); HREIMS *m*/*z* 141.1276 [M-C_10_H_21_]^+^ (calcd. for C_9_H_17_O, 141.1273).

##### (*S*)-Nonadec-1-en-9-ol (*S-* **7**)

According to GSP2, *n*-nonylmagnesium bromide (12.97 mL, 1 m in Et_2_O, 12.96 mmol, 2 equiv) was added to a suspension of copper iodide (1.23 g, 6.48 mmol, 1 equiv) in THF (100 mL). (*R*)-2-(Oct-7-en-1-yl)­oxirane (*R* **-6**), 1.00 g, 6.48 mmol, 1 equiv) was added to the solution. (*S*)-Nonadec-1-en-9-ol (*S* **-7**) was obtained as a colorless solid (1.17 g, 4.16 mmol, 61%). IR (neat) ν_max_ 3324, 2917, 2849, 1641, 1460, 1350, 1117, 1034, 991, 909, 859, 721, 628 cm^–1^; ^1^H NMR (CDCl_3_, 300 MHz) δ (ppm) 5.81 (ddt, *J* = 17.1, 10.2, 6.6 Hz, 1H), 4.99 (ddt, *J* = 17.1, 2.1, 1.6 Hz, 1H), 4.93 (ddt, *J* = 10.2, 2.2, 1.2 Hz, 1H), 3.62–3.53 (m, 1H), 2.09–1.98 (m, 2H), 1.49–1.21 (m, 29H). 0.92–0.83 (m, 3H); ^13^C NMR (CDCl_3_, 75 MHz) δ (ppm) 139.1 (CH), 114.2 (CH_2_), 72.0 (CH), 37.49 (CH_2_), 37.45 (CH_2_), 38.8 (CH_2_), 31.9 (CH_2_), 29.7 (CH_2_), 29.63 (CH_2_), 29.61 (2C, CH_2_), 29.5 (CH_2_), 29.3 (CH_2_), 29.1 (CH_2_), 28.9 (CH_2_), 25.7 (CH_2_), 25.6 (CH_2_), 22.7 (CH_2_), 14.1 (CH_3_); EIMS *m*/*z* 264 ([M-H_2_O]^+^, 3), 171 (14), 141 (8), 123 (46), 111 (21), 97 (68), 83 (61), 81 (100), 69 (56), 67 (61), 57 (49), 55 (91), 43 (54), 41 (69); HREIMS *m*/*z* 141.1276 [M-C_10_H_21_]^+^ (calcd. for C_9_H_17_O, 141.1273).

#### General Synthetic Protocol 3 (GSP3) for Steglich Esterification of Secondary Alcohols

The secondary alcohol (1 equiv) was dissolved in dichloromethane (DCM), and triethylamine (TEA, 10 equiv), 1-ethyl-3-(3′-dimethylaminopropyl)­carbodiimide hydrochloride (EDCI, 5 equiv), 4-(*N*,*N*-dimethylamino)­pyridine (DMAP, 0.5 equiv) and the carboxylic acid (5 equiv) were added subsequently to the solution.^37^ The reaction mixture was stirred at room temperature for 18 h and afterward hydrolyzed by the addition of water. After phase separation, the aqueous phase was extracted with DCM. The combined organic phases were washed with a solution of hydrochloric acid (1 M in water) and brine, then dried over magnesium sulfate. The solvent was removed under reduced pressure, and the ester was isolated by column chromatography (20:1, pentane/diethyl ether).

##### (*R*)-Pentadec-14-en-7-yl Dec-9-enoate

According to GSP3, (*R*)-pentadec-14-en-7-ol (300 mg, 1.31 mmol, 1 equiv) was dissolved in DCM (30 mL), and TEA (1.83 mL, 13.16 mmol, 10 equiv), EDCI (1.26 g, 6.57 mmol, 5 equiv), DMAP (78 mg, 0.64 mmol, 0.5 equiv) and 9-decenoic acid (1.22 g, 6.55 mmol, 5 equiv) were added subsequently. (*R*)-Pentadec-14-en-7-yl dec-9-enoate was isolated as a colorless liquid (406 mg, 1.22 mmol, 93%). IR (neat) ν_max_ 3074, 2923, 2855, 1732, 1640, 1455, 1369, 1242, 1176, 991, 908, 724, 635 cm^–1^; ^1^H NMR (CDCl_3_, 300 MHz) δ (ppm) 5.80 (ddt, *J* = 16.9, 10.1, 6.7 Hz, 2H), 5.03–4.95 (m, 2H), 4.95–4.89 (m, 2H), 4.86 (quin, *J* = 6.2 Hz, 1H), 2.28 (t, *J* = 7.5 Hz, 2H), 2.03 (tdt, *J* = 6.7, 5.3, 1.4 Hz, 4H), 1.66–1.23 (m, 30H), 0.93–0.82 (m, 3H) ; ^13^C NMR (CDCl_3_, 75 MHz) δ (ppm) 173.7 (C_q_), 139.1 (2C, CH), 114.2 (2C, CH_2_), 74.0 (CH), 34.7 (CH_2_), 34.2 (CH_2_), 34.1 (CH_2_), 33.7 (2C, CH_2_), 31.7 (CH_2_), 29.4 (CH_2_), 29.2 (CH_2_), 29.13 (CH_2_), 29.10 (CH_2_), 29.0 (CH_2_), 28.9 (CH_2_), 28.84 (CH_2_), 28.80 (CH_2_), 25.27 (CH_2_), 25.25 (CH_2_), 25.1 (CH_2_), 22.6 (CH_2_), 14.1 (CH_3_); HREIMS *m*/*z* 208.2188 [M-C_10_H_18_O_2_]^+^ (calcd. for C_15_H_26_ 208.2185).

##### (*R*)-Octadec-1-en-9-yl Dec-9-enoate

According to GSP3 (*R*)-octadec-1-en-9-ol (500 mg, 1.86 mmol, 1 equiv) was dissolved in DCM (50 mL), and TEA (2.62 mL, 18.92 mmol, 10 equiv), EDCI (1.81 g, 9.46 mmol, 5 equiv), DMAP (113 mg, 0.93 mmol, 0.5 equiv) and 9-decenoic acid (1.74 g, 9.46 mmol, 5 equiv) were added subsequently. (*R*)-Octadec-1-en-9-yl dec-9-enoate was isolated as a colorless liquid (709 mg, 1.68 mmol, 91%). IR (neat) ν_max_ 3074, 2923, 2855, 1732, 1640, 1455, 1369, 1242, 1176, 991, 908, 724, 635 cm^–1^; ^1^H NMR (CDCl_3_, 300 MHz) δ (ppm) 5.80 (ddt, *J* = 17.0, 10.1, 6.7 Hz, 2H), 5.03–4.95 (m, 2H), 4.95–4.89 (m, 2H), 4.86 (quin, *J* = 6.2 Hz, 1H), 2.28 (t, *J* = 7.5 Hz, 1H), 2.08–1.98 (m, 4H), 1.68–1.20 (m, 38H), 0.90–0.84 (m, 3H); ^13^C NMR (CDCl_3_, 75 MHz) δ (ppm) 173.7 (C_q_), 139.1 (2C, CH), 114.2 (2C, CH_2_), 74.0 (CH), 34.7 (CH_2_), 34.2 (CH_2_), 34.1 (CH_2_), 33.8 (2C, CH_2_), 31.9 (CH_2_), 29.56 (CH_2_), 29.53 (2C, CH_2_), 29.4 (CH_2_), 29.3 (CH_2_), 29.1 (2C, CH_2_), 29.0 (CH_2_), 28.95 (CH_2_), 28.9 (CH_2_), 28.8 (CH_2_), 25.3 (CH_2_), 25.2 (CH_2_), 25.1 (CH_2_), 22.7 (CH_2_), 14.1 (CH_3_); EIMS *m*/*z* 420 ([M]^+^, 1), 293 (5), 250 (10), 153 (30), 135 (82), 124 (18), 110 (37), 96 (55), 83 (61), 69 (79), 55 (100), 41 (51); HREIMS *m*/*z* 250.2656 [M-C_10_H_18_O_2_]^+^ (calcd. for C_18_H_34_ 250.2655).

##### (*R*)-Nonadec-1-en-9-yl Dec-9-enoate (*R-* **8**)

According to GSP3, (*R*)-nonadec-1-en-9-ol (*R* **-7**, 500 mg, 1.86 mmol, 1 equiv) was dissolved in DCM (50 mL), and TEA (2.62 mL, 18.92 mmol, 10 equiv), EDCI (1.81 g, 9.46 mmol, 5 equiv), DMAP (113 mg, 0.93 mmol, 0.5 equiv) and 9-decenoic acid (1.74 g, 9.46 mmol, 5 equiv) were added subsequently. (*R*)-Nonadec-1-en-9-yl dec-9-enoate (*R* **-8**) was isolated as a colorless liquid (565 mg, 1.30 mmol, 73%). IR (neat) ν_max_ 3074, 2922, 2855, 1732, 1640, 1455, 1369, 1242, 1176, 991, 908, 724, 635 cm^–1^; ^1^H NMR (CDCl_3_, 300 MHz) δ (ppm) 5.80 (ddt, *J* = 16.9, 10.1, 6.6 Hz, 2H), 4.98 (ddt, *J* = 17.1, 2.2, 1.5 Hz, 2H), 4.92 (ddt, *J* = 10.2, 2.1, 1.3 Hz, 2H), 4.86 (quin, *J* = 6.3 Hz, 1H), 2.28 (t, *J* = 7.5 Hz, 2H), 2.11–1.96 (m, 4H), 1.67–1.19 (m, 38H), 0.92–0.82 (m, 3H); ^13^C NMR (CDCl_3_, 75 MHz) δ (ppm) 173.6 (C_q_), 139.1 (2C, CH), 114.2 (2C, CH_2_), 74.0 (CH), 34.7 (CH_2_), 34.2 (CH_2_), 34.1 (CH_2_), 33.8 (2C, CH_2_), 31.9 (CH_2_), 29.61 (CH_2_), 29.58 (CH_2_), 29.55 (CH_2_), 29.54 (CH_2_), 29.4 (CH_2_), 29.3 (CH_2_), 29.13 (CH_2_), 19.12 (CH_2_), 29.0 (CH_2_), 28.95 (CH_2_), 28.85 (CH_2_), 28.82 (CH_2_), 25.32 (CH_2_), 25.26 (CH_2_), 25.15 (CH_2_), 22.7 (CH_2_), 14.1 (CH_3_); EIMS *m*/*z* 293 (5), 264 (19), 222 (10), 170 (8), 153 (40), 135 (84), 110 (45), 96 (69), 83 (63), 69 (84), 55 (100), 41 (47); HREIMS *m*/*z* 293.2475 [M-C_10_H_21_]^+^ (calcd. for C_19_H_33_O_2_, 293.2475).

##### (*S*)-Nonadec-1-en-9-yl Dec-9-enoate (*S-* **8**)

According to GSP3, (*S*)-nonadec-1-en-9-ol (*S* **-6**, 500 mg, 1.86 mmol, 1 equiv) was dissolved in DCM (50 mL), and TEA (2.62 mL, 18.92 mmol, 10 equiv), EDCI (1.81 g, 9.46 mmol, 5 equiv), DMAP (113 mg, 0.93 mmol, 0.5 equiv) and 9-decenoic acid (1.74 g, 9.46 mmol, 5 equiv) were added subsequently. (*S*)-Nonadec-1-en-9-yl dec-9-enoate (*S* **-6**) was isolated as a colorless liquid (398 mg, 0.92 mmol, 52%). IR (neat) ν_max_ 3074, 2922, 2855, 1732, 1640, 1455, 1369, 1242, 1176, 991, 908, 724, 635 cm^–1^; ^1^H NMR (CDCl_3_, 300 MHz) δ (ppm) 5.80 (ddt, *J* = 16.9, 10.1, 6.6 Hz, 2H), 4.98 (ddt, *J* = 17.1, 2.2, 1.5 Hz, 2H), 4.92 (ddt, *J* = 10.2, 2.1, 1.3 Hz, 2H), 4.86 (quin, *J* = 6.3 Hz, 1H), 2.28 (t, *J* = 7.5 Hz, 2H), 2.11–1.96 (m, 4H), 1.67–1.19 (m, 38H), 0.92–0.82 (m, 3H); ^13^C NMR (CDCl_3_, 75 MHz) δ (ppm) 173.6 (C_q_), 139.1 (2C, CH), 114.2 (2C, CH_2_), 74.0 (CH), 34.7 (CH_2_), 34.2 (CH_2_), 34.1 (CH_2_), 33.8 (2C, CH_2_), 31.9 (CH_2_), 29.61 (CH_2_), 29.58 (CH_2_), 29.55 (CH_2_), 29.54 (CH_2_), 29.4 (CH_2_), 29.3 (CH_2_), 29.13 (CH_2_), 19.12 (CH_2_), 29.0 (CH_2_), 28.95 (CH_2_), 28.85 (CH_2_), 28.82 (CH_2_), 25.32 (CH_2_), 25.26 (CH_2_), 25.15 (CH_2_), 22.7 (CH_2_), 14.1 (CH_3_); EIMS *m*/*z* 293 (5), 264 (19), 222 (10), 170 (8), 153 (40), 135 (84), 110 (45), 96 (69), 83 (63), 69 (84), 55 (100), 41 (47); HREIMS *m*/*z* 293.2475 [M-C_10_H_21_]^+^ (calcd. for C_19_H_33_O_2_, 293.2475).

#### General Synthetic Protocol 4 (GSP4) for Ring Closing Metathesis and Hydrogenation of Dienes

The diene (1 equiv) was dissolved in DCM (degassed) or toluene (degassed), and hexafluorobenzene (10 equiv) and tetrafluoro-*p*-benzoquinone (0.1 equiv) were added to the solution. [1,3-Bis­(2-methylphenyl)-2-imidazolidinyliden]-dichloro-(2-isopropoxyphenylmethylen)-ruthenium­(II) (Stewart-Grubbs catalyst, C571, 0.1 equiv) was added to the reaction mixture, and the mixture was heated to reflux for 48 h when DCM was used.^38^ After the addition of another 0.1 eq. of C571, the solution was again heated to reflux for 48 h. For toluene, only heating to reflux for 18 h was required, without a second catalyst addition. After cooling to room temperature, silica gel (1 g) was added, and the solvents were removed under reduced pressure. The crude product mixture was purified by column chromatography (100:1, pentane/diethyl ether). The pure macrocyclic lactone was then dissolved in ethyl acetate (1 mL). To this solution, Pd/C (5 mg) was added, and the suspension was stirred for 10 min under a H_2_ atmosphere at room temperature. The reaction mixture was filtered through a short Celite plug, and the solvent was removed under reduced pressure. The saturated macrolactone was isolated by column chromatography (100:1, pentane/diethyl ether.

##### (*R*)-17-Tricosanolide

According to GSP4, (*R*)-pentadec-14-en-7-yl dec-9-enoate (200 mg, 0.53 mmol, 1 equiv) was dissolved in DCM (200 mL), and to the solution were added hexafluorobenzene (0.60 mL, 0.97 g, 5.30 mmol, 10 equiv) and tetrafluoro-*p*-benzoquinone (9.6 mg, 0.053 mmol, 0.1 equiv). C571 (30.2 mg, 0.071 mmol, 0.1 equiv) was added to the reaction mixture. After hydrogenation, (*R*)-17-tricosanolide was isolated as a colorless liquid (56.1 mg, 0.16 mmol, 30% over two steps). IR (neat) ν_max_ 3074, 2923, 2855, 1732, 1640, 1455, 1369, 1242, 1176, 991, 908, 724, 635 cm^–1^; ^1^H NMR (CDCl_3_, 300 MHz) δ (ppm) 4.83 (quin, *J* = 7.2 Hz, 1H), 2.39–2.21 (m, 2H), 1.69–1.34 (m, 5H), 1.33–1.05 (m, 31H), 0.87–0.72 (m, 5H); ^13^C NMR (CDCl_3_, 75 MHz) δ (ppm) 173.7 (C_q_), 74.1 (CH), 34.9 (CH_2_), 34.3 (CH_2_), 34.1 (CH_2_), 31.7 (CH_2_), 29.2 (CH_2_), 28.8 (CH_2_), 28.4 (CH_2_), 28.2 (CH_2_), 28.0 (CH_2_), 27.4 (CH_2_), 27.08 (CH_2_), 27.07 (CH_2_), 26.9 (CH_2_), 26.4 (CH_2_), 26.0 (CH_2_), 25.4 (CH_2_), 25.1 (CH_2_), 24.6 (CH_2_), 22.6 (CH_2_), 22.2 (CH_2_), 14.1 (CH_3_); HREIMS *m*/*z* 334.3231 [M-H_2_O]^+^ (calcd. for C_23_H_42_O 334.3230).

##### (*R*)-17-Hexacosanolide

According to GSP4, (*R*)-octadec-1-en-9-yl dec-9-enoate (300 mg, 0.71 mmol, 1 equiv) was dissolved in DCM (250 mL), and hexafluorobenzene (0.82 mL, 1.33 g, 7.13 mmol, 10 equiv) and tetrafluoro-*p*-benzoquinone (12.8 mg, 0.071 mmol, 0.1 equiv) were added to the solution. Catalyst C571 (40.5 mg, 0.071 mmol, 0.1 equiv) was then added. After hydrogenation, (*R*)-17-hexacosanolide was isolated as a colorless liquid (163 mg, 0.41 mmol, 57% over two steps). [α]_D_^21^ −0.9 (c 1.0, CHCl_3_); IR (neat) ν_max_ 2921, 2855, 1731, 1455, 1351, 1240, 1176, 721 cm^–1^; ^1^H NMR (CDCl_3_, 300 MHz) δ (ppm) 4.95–4.83 (m, 1H), 2.38–2.22 (m, 2H), 1.77–1.41 (m, 6H), 1.41–1.18 (m, 38H), 0.91–0.83 (m, 3H); ^13^C NMR (CDCl_3_, 75 MHz) δ (ppm) 173.7 (C_q_), 74.1 (CH), 34.9 (CH_2_), 34.3 (CH_2_), 34.2 (CH_2_), 34.1 (CH_2_), 31.88 (CH_2_), 31.86 (CH_2_), 29.5 (2C, CH_2_), 29.3 (CH_2_), 28.8 (CH_2_), 28.4 (CH_2_), 28.2 (CH_2_), 28.0 (CH_2_), 27.4 (CH_2_), 27.1 (2C, CH_2_), 27.0 (CH_2_), 26.9 (CH_2_), 26.5 (CH_2_), 26.0 (CH_2_), 25.4 (CH_2_), 25.1 (CH_2_), 24.6 (CH_2_), 22.7 (CH_2_), 14.1 (CH_3_); EIMS *m*/*z* 394 ([M]^+^, 2), 376 ([M-H_2_O]^+^, 43), 267 (19), 249 (12), 238 (20), 125 (21), 111 (32), 97 (61), 83 (65), 69 (74), 57 (59), 55 (100), 43 (72), 41 (55); HREIMS *m*/*z* 376.3699 [M-H_2_O]^+^ (calcd. for C_26_H_48_O 376.3700).

##### (*R*)-17-Heptacosanolide (*R* **-2**)

According to GSP4, (*R*)-nonadec-1-en-9-yl dec-9-enoate (*R* **-8**, 100 mg, 0.23 mmol, 1 equiv) was dissolved in toluene (50 mL), and to the solution were added hexafluorobenzene (0.26 mL, 428 mg, 2.3 mmol, 10 equiv) and tetrafluoro-*p*-benzoquinone (4.2 mg, 0.023 mmol, 0.1 equiv). C571 (13.1 mg, 0.023 mmol, 0.1 equiv) was added to the reaction mixture. After hydrogenation, (*R*)-17-heptacosanolide (*R* **-2**) was isolated as a colorless liquid (70.9 mg, 0.17 mmol, 75% over two steps). [α]_D_^21^ −1.0 (c 1.0, CHCl_3_); IR (neat) ν_max_ 2921, 2855, 1731, 1455, 1351, 1240, 1176, 721 cm^–1^; ^1^H NMR (CDCl_3_, 300 MHz) δ (ppm) 4.90 (quin, *J* = 5.8 Hz, 1H), 2.38–2.21 (m, 2H), 1.77–1.41 (m, 6H), 1.40–1.18 (m, 40H), 0.91–0.84 (m, 3H); ^13^C NMR (CDCl_3_, 75 MHz) δ (ppm) 173.7 (C_q_), 74.1 (CH), 34.9 (CH_2_), 34.3 (CH_2_), 34.1 (CH_2_), 31.9 (CH_2_), 29.6 (CH_2_), 29.58 (CH_2_), 29.55 (CH_2_), 29.54 (CH_2_), 29.3 (CH_2_), 28.8 (CH_2_), 28.4 (CH_2_), 28.2 (CH_2_), 28.0 (CH_2_), 27.4 (CH_2_), 27.1 (2C, CH_2_), 27.0 (CH_2_), 26.9 (CH_2_), 26.5 (CH_2_), 26.0 (CH_2_), 25.4 (CH_2_), 25.1 (CH_2_), 24.8 (CH_2_), 22.7 (CH_2_), 14.1 (CH_3_); EIMS *m*/*z* 408 ([M]^+^, 2), 390 ([M-H_2_O]^+^, 43), 267 (19), 249 (12), 238 (20), 125 (21), 111 (32), 97 (61), 83 (65), 69 (74), 57 (59), 55 (100), 43 (72), 41 (55); HREIMS *m*/*z* 408.3971 [M]^+^ (calcd. for C_27_H_52_O_2_, 408.3967), 390.3857 [M-H_2_O]^+^ (calcd. for C_27_H_50_O_1_, 390.3856).

##### (*S*)-17-Heptacosanolide (*S* **-2**)

According to GSP4, (*S*)-nonadec-1-en-9-yl dec-9-enoate (*S* **-7**, 100 mg, 0.23 mmol, 1 equiv) was dissolved in toluene (50 mL), and to the solution were added hexafluorobenzene (0.26 mL, 428 mg, 2.3 mmol, 10 equiv) and tetrafluoro-*p*-benzoquinone (4.2 mg, 0.023 mmol, 0.1 equiv). C571 (13.1 mg, 0.023 mmol, 0.1 equiv) was added to the reaction mixture. After hydrogenation, (*S*)-17-heptacosanolide (*S* **-2**) was isolated as a colorless liquid (71.2 mg, 0.17 mmol, 75% over two steps). [α]_D_^21^ +2.2 (c 1.0, CHCl_3_); IR (neat) ν_max_ 2921, 2855, 1731, 1455, 1351, 1240, 1176, 721 cm^–1^; ^1^H NMR (CDCl_3_, 300 MHz) δ (ppm) 4.90 (quin, *J* = 5.8 Hz, 1H), 2.38–2.21 (m, 2H), 1.77–1.41 (m, 6H), 1.40–1.18 (m, 40H), 0.91–0.84 (m, 3H); ^13^C NMR (CDCl_3_, 75 MHz) δ (ppm) 173.7 (C_q_), 74.1 (CH), 34.9 (CH_2_), 34.3 (CH_2_), 34.1 (CH_2_), 31.9 (CH_2_), 29.6 (CH_2_), 29.58 (CH_2_), 29.55 (CH_2_), 29.54 (CH_2_), 29.3 (CH_2_), 28.8 (CH_2_), 28.4 (CH_2_), 28.2 (CH_2_), 28.0 (CH_2_), 27.4 (CH_2_), 27.1 (2C, CH_2_), 27.0 (CH_2_), 26.9 (CH_2_), 26.5 (CH_2_), 26.0 (CH_2_), 25.4 (CH_2_), 25.1 (CH_2_), 24.8 (CH_2_), 22.7 (CH_2_), 14.1 (CH_3_); EIMS *m*/*z* 408 ([M]^+^, 2), 390 ([M-H_2_O]^+^, 43), 267 (19), 249 (12), 238 (20), 125 (21), 111 (32), 97 (61), 83 (65), 69 (74), 57 (59), 55 (100), 43 (72), 41 (55); HREIMS *m*/*z* 408.3971 [M]^+^ (calcd. for C_27_H_52_O_2_, 408.3962), 390.3857 [M-H_2_O]^+^ (calcd. for C_27_H_50_O_1_, 390.3856).

## Supplementary Material





## Notes

NMR raw data (ZIP)
Sample Table, mass spectral data, NMR spectra, IR spectra, phylogeny (PDF)
