# Supplementary material for: Large Macrocyclic Lactones as Specific Cuticular Lipids of Eueides Butterflies
Source: J Nat Prod. 2026 Mar 24;89(4):1276–86. doi: 10.1021/acs.jnatprod.6c00128 (PMC13122645; doi:10.1021/acs.jnatprod.6c00128)
Supplement: Supplementary file 2 [file np6c00128_si_002.pdf]

Supporting Information

Large Macrocyclic Lactones as Specific Cuticular Lipids of *Eueides* Butterflies

Ozan Mehmet Solak[a], *Stephanie Ehlers*[a], *Bruna Cama*[b], *Kanchon Dasmahapatra*[b], *Chris D. Jiggins*[c], *Stefan Schulz*[a,]*

[a]Institute of Organic Chemistry, Technische Universität Braunschweig, 38106 Braunschweig, Germany

[b]Department of Biology, University of York, Heslington YO10 5DD, United Kingdom

[c]Department of Zoology, University of Cambridge, Cambridge CB2 3EJ, United Kingdom

*Stefan.schulz@tu-braunschweig.de

Contents

1. Collection Samples 2
2. Mass Spectra 2
3. High-Resolution EI-Orbitrap Mass Spectra of Compounds A and B 24
4. Total ion chromatogram of a collection sample 26
5. Phylogeny of *Eueides* butterflies 28
6. NMR Spectra 29
7. IR Spectra 43
8. References 44

2 Collection Samples

Table S1. Codes and country of collection of individual specimens from the collection of *Eueides* butterflies from York University.

Species	sex	Id	Location	Year
E. vibilia	female	05-1285	Peru	2005
	male	09-147	Peru	2001
E. lybia	female	LQ13-131	Panama	2013
	male	LQ13-115	Panama	2013
E. isabella	female	09-193	Peru	2009
	male	09-182	Peru	2009
E. tales	female	09-132	Peru	2009
	male	09-256	Peru	2009
E. aliphera	female	LQ13-81	Panama	2013
	male	LQ13-80	Panama	2013

2 Mass Spectra

Table S2. Characteristic ions in mass spectra indicating ring size and chain length of macrocyclic lactones. Top line: M^+ and M^+-18 ions. Left column: ions indicating ring size. Abbreviations indicate the ring size as, e. g. -17-olide, and the chain length, e. g. C28. Compounds occurring in *Eueides* sp. are shown in red.

	348-366	362-380	376-394	390-408	404-422	418-436	432-450	446-464	446-464	446-464
224-235-253	C24-16-olide	C25-16-olide	C26-16-olide	C27-16-olide	C28-16-olide	C29-16-olide	C30-16-olide	C31-16-olide	C32-16-olide	C33-16-olide
238-249-267	C24-17-olide	C25-17-olide	C26-17-olide	C27-17-olide	C28-17-olide	C29-17-olide	C30-17-olide	C31-17-olide	C32-17-olide	C33-17-olide
252-263-281	C24-18-olide	C25-18-olide	C26-18-olide	C27-18-olide	C28-18-olide	C29-18-olide	C30-18-olide	C31-18-olide	C32-18-olide	C33-18-olide
266-277-295	C24-19-olide	C25-19-olide	C26-19-olide	C27-19-olide	C28-19-olide	C29-19-olide	C30-19-olide	C31-19-olide	C32-19-olide	C33-19-olide
280-291-309	C24-20-olide	C25-20-olide	C26-20-olide	C27-20-olide	C28-20-olide	C29-20-olide	C30-20-olide	C31-20-olide	C32-20-olide	C33-20-olide
294-305-323	C24-21-olide	C25-21-olide	C26-21-olide	C27-21-olide	C28-21-olide	C29-21-olide	C30-21-olide	C31-21-olide	C32-21-olide	C33-21-olide
308-319-337	C24-22-olide	C25-22-olide	C26-22-olide	C27-22-olide	C28-22-olide	C29-22-olide	C30-22-olide	C31-22-olide	C32-22-olide	C33-22-olide
322-333-351	C24-23-olide	C25-23-olide	C26-23-olide	C27-23-olide	C28-23-olide	C29-23-olide	C30-23-olide	C31-23-olide	C32-23-olide	C33-23-olide
336-347-365		C25-24-olide	C26-24-olide	C27-24-olide	C28-24-olide	C29-24-olide	C30-24-olide	C31-24-olide	C32-24-olide	C33-24-olide
350-361-379			C26-25-olide	C27-25-olide	C28-25-olide	C29-25-olide	C30-25-olide	C31-25-olide	C32-25-olide	C33-25-olide
364-375-393				C27-26-olide	C28-26-olide	C29-26-olide	C30-26-olide	C31-26-olide	C32-26-olide	C33-26-olide
378-389-407					C28-27-olide	C29-27-olide	C30-27-olide	C31-27-olide	C32-27-olide	C33-27-olide
392-403-421						C29-28-olide	C30-28-olide	C31-28-olide	C32-28-olide	C33-28-olide

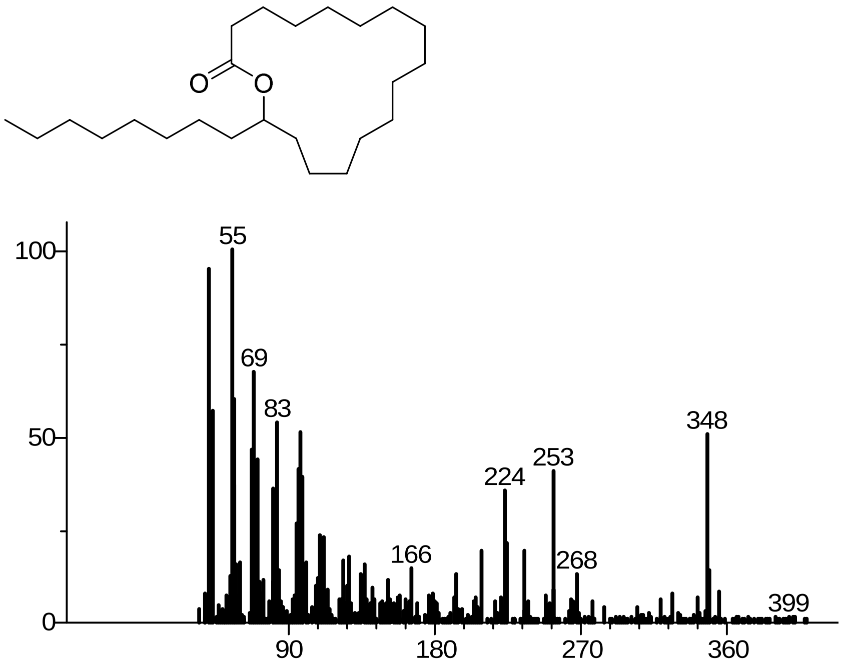

Figure S1. Mass spectrum and structure of tetracosan-16-olide.

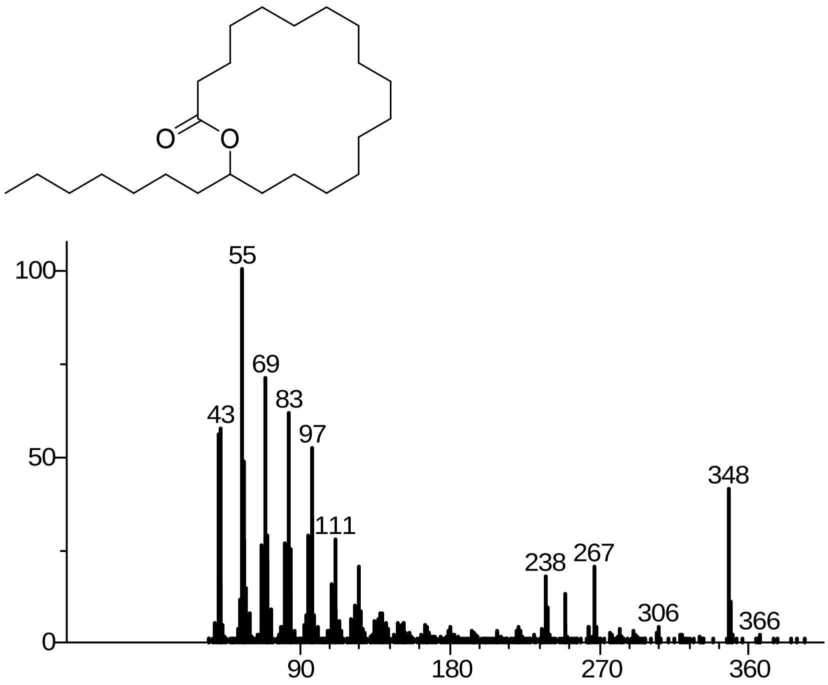

Figure S2. Mass spectrum and structure of tetracosan-17-olide.

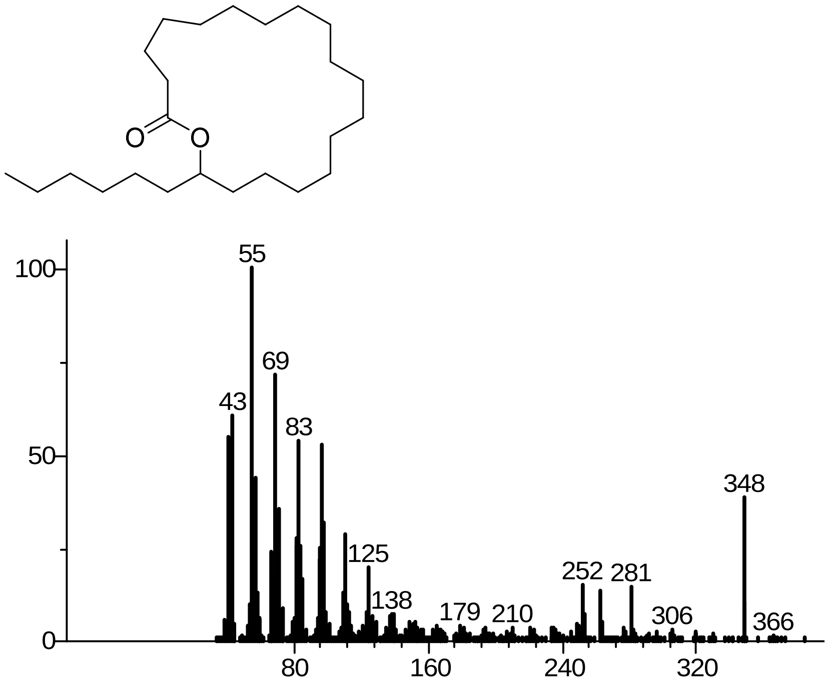

Figure S3. Mass spectrum and structure of tetracosan-18-olide.

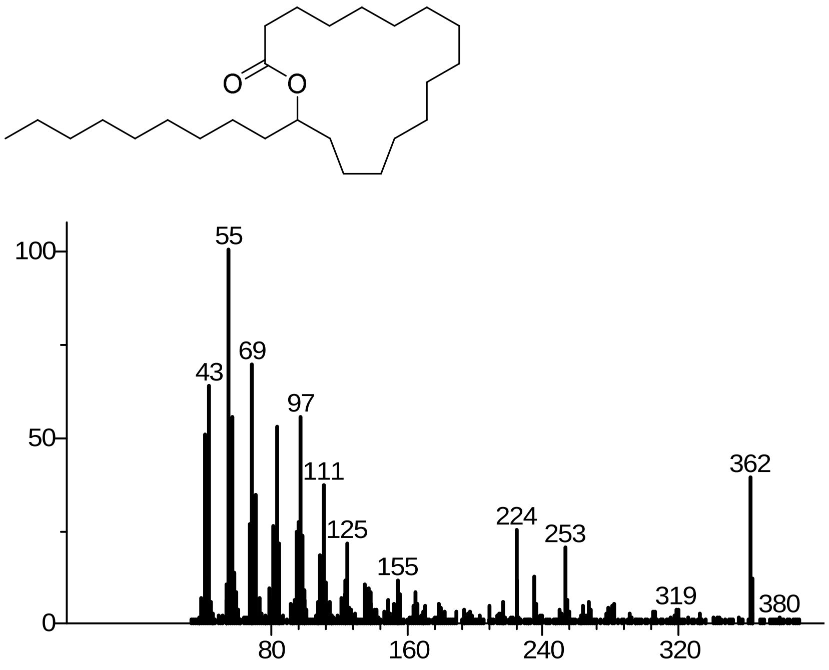

Figure S4. Mass spectrum and structure of pentacosan-16-olide.

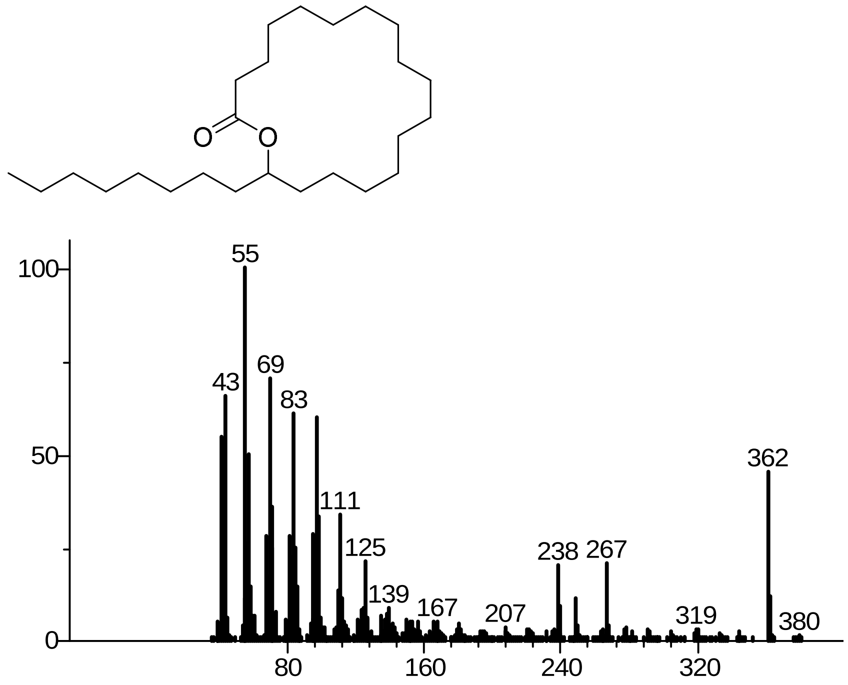

Figure S5. Mass spectrum and structure of pentacosan-17-olide.

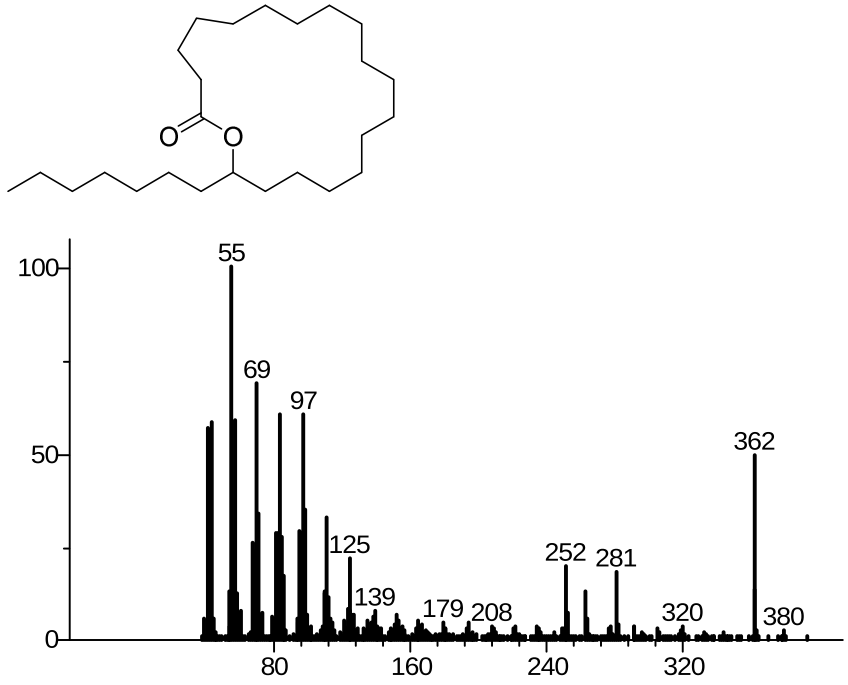

Figure S6. Mass spectrum and structure of pentacosan-18-olide.

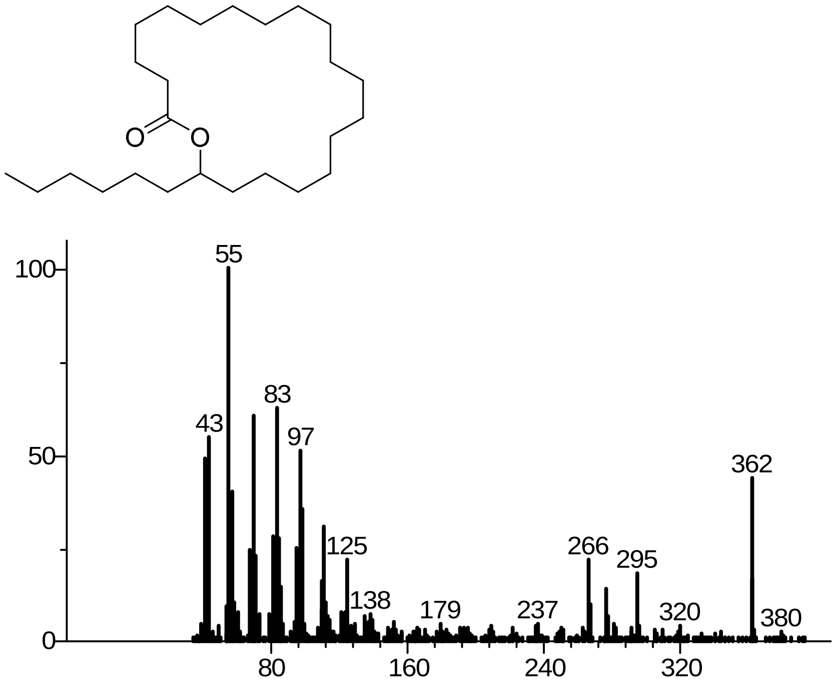

Figure S7. Mass spectrum and structure of pentacosan-19-olide.

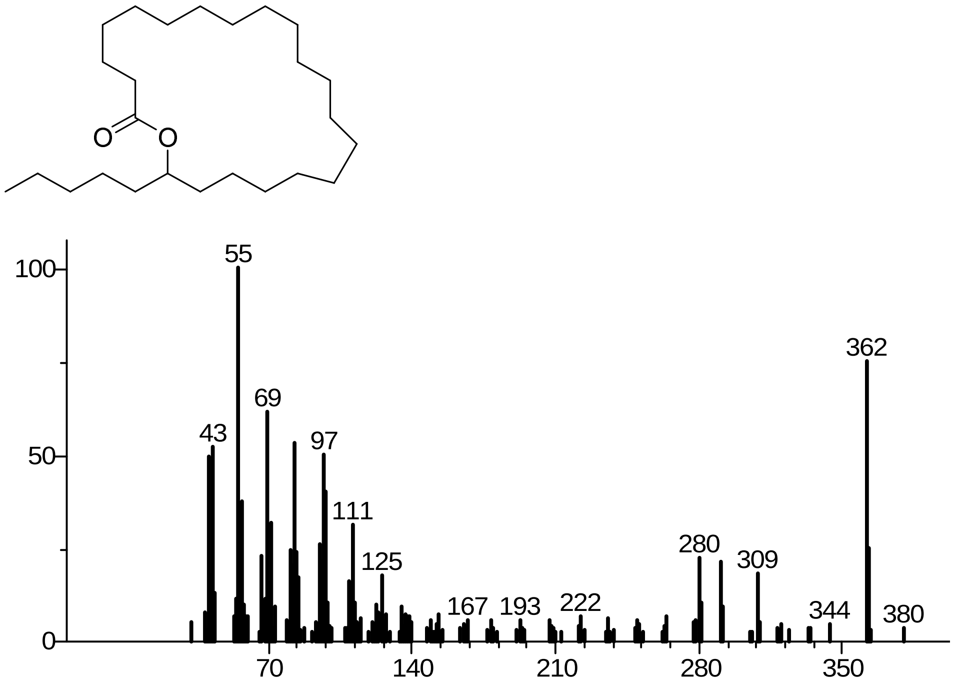

Figure S8. Mass spectrum and structure of pentacosan-20-olide.

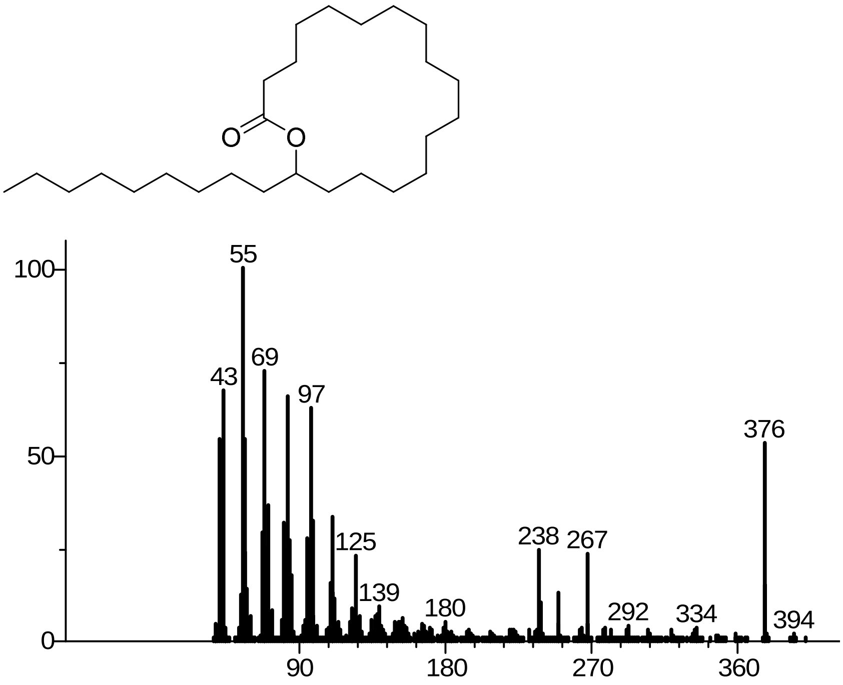

Figure S9. Mass spectrum and structure of hexacosan-17-olide.

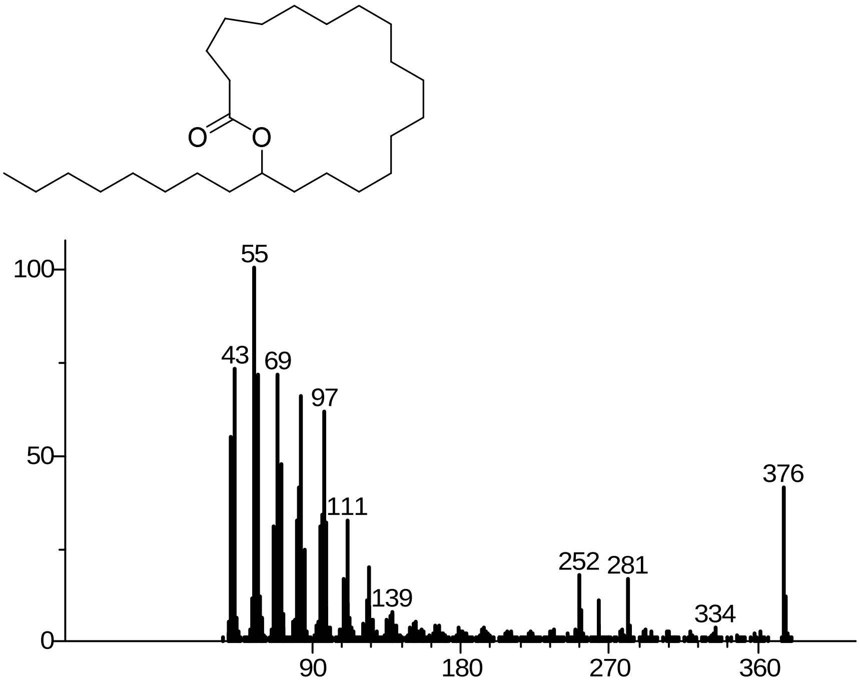

Figure S10. Mass spectrum and structure of hexacosan-18-olide.

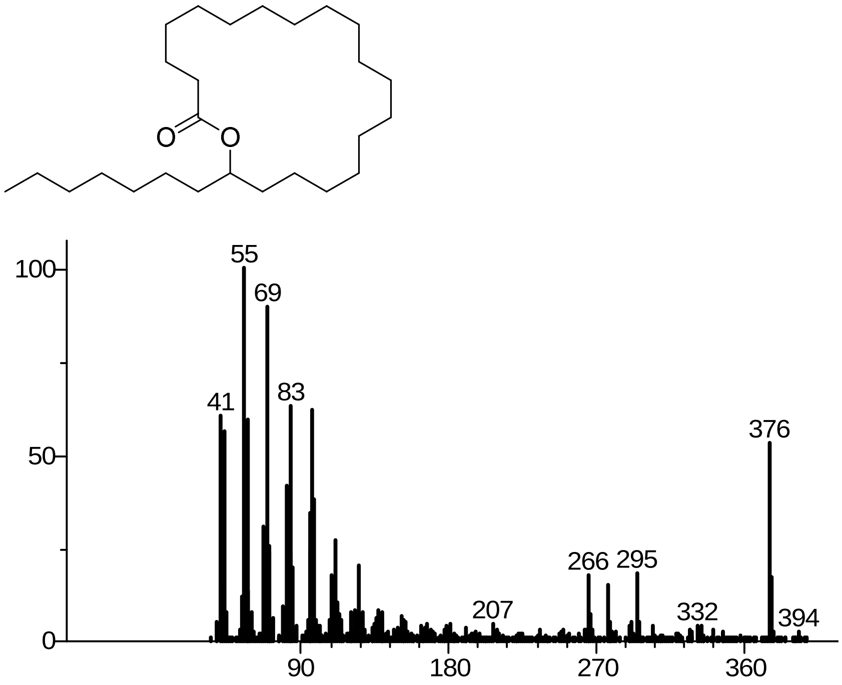

Figure S11. Mass spectrum and structure of hexacosan-19-olide.

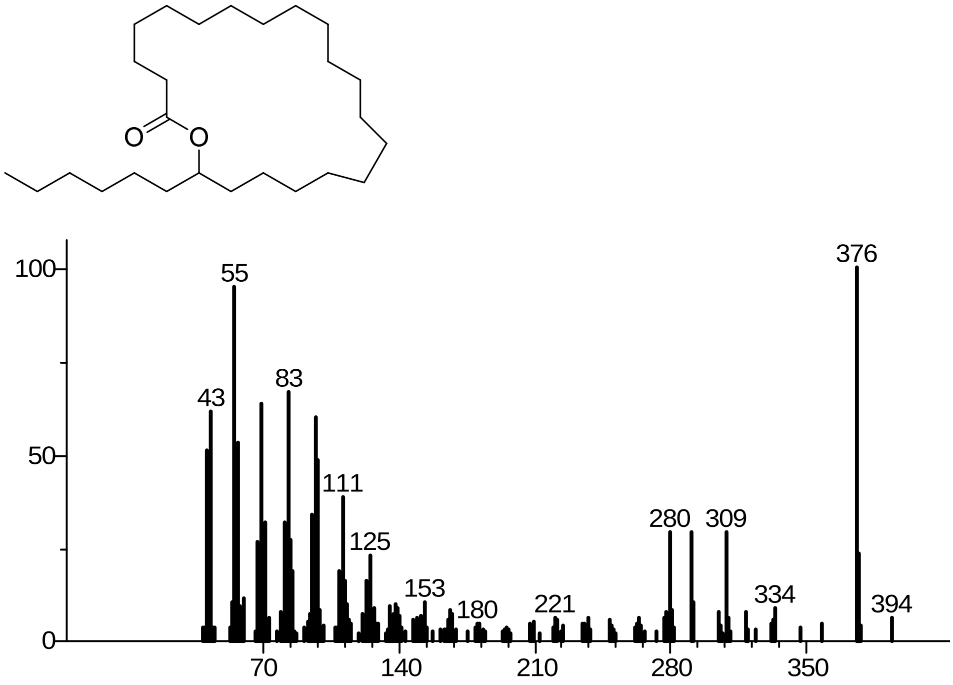

Figure S12. Mass spectrum and structure of hexacosan-20-olide.

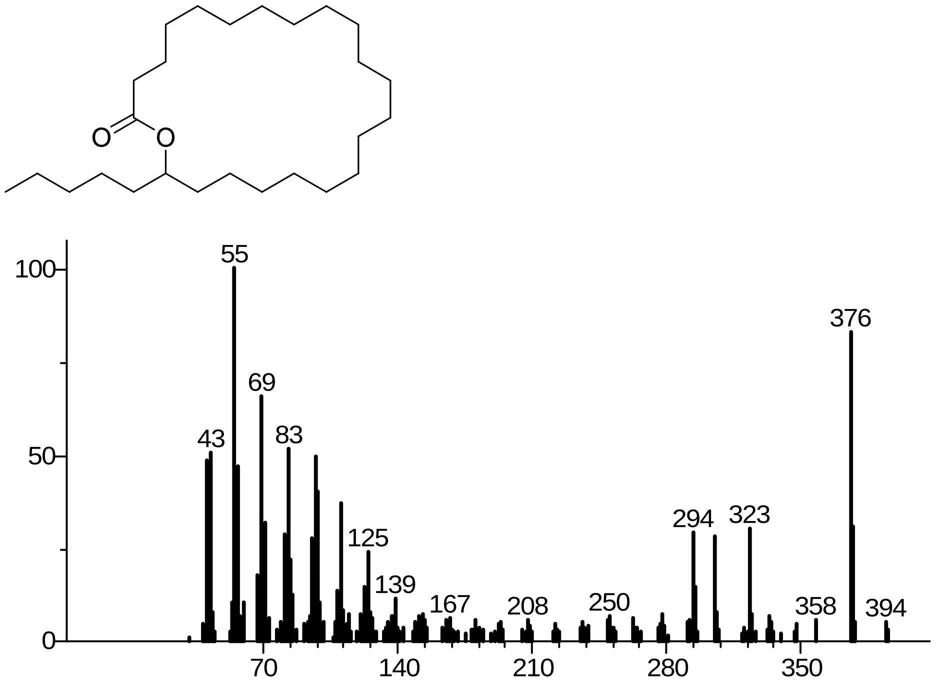

Figure S13. Mass spectrum and structure of hexacosan-21-olide.

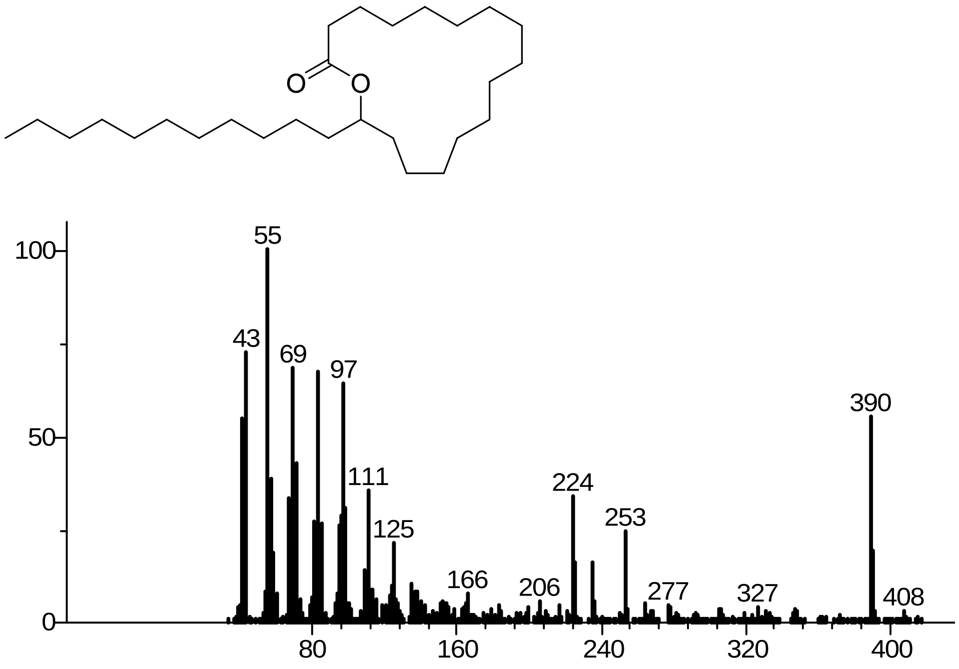

Figure S14. Mass spectrum and structure of heptacosan-16-olide.

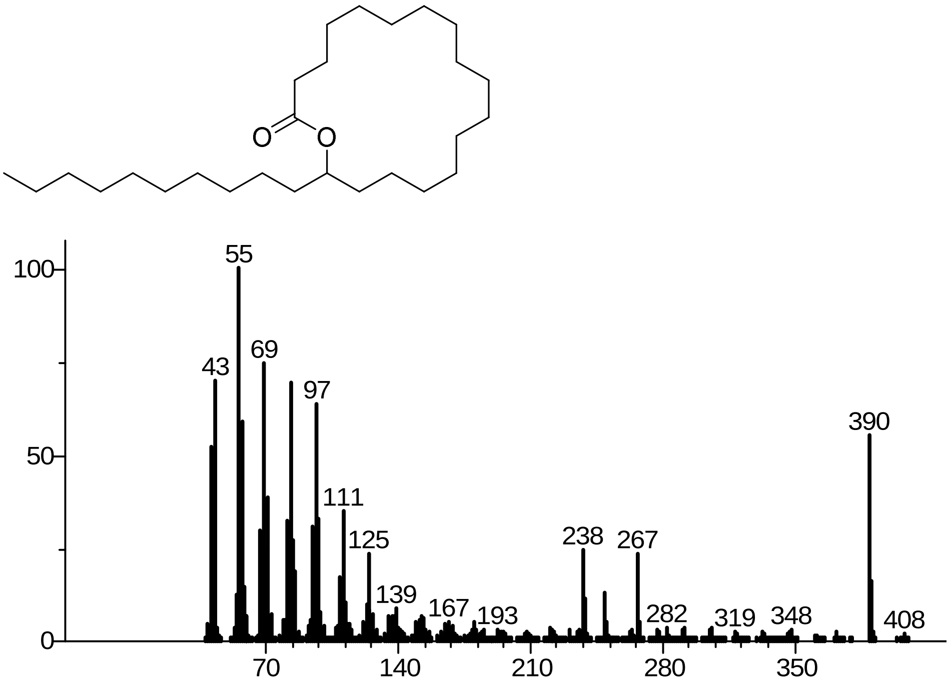

Figure S15. Mass spectrum and structure of heptacosan-17-olide (**2**).

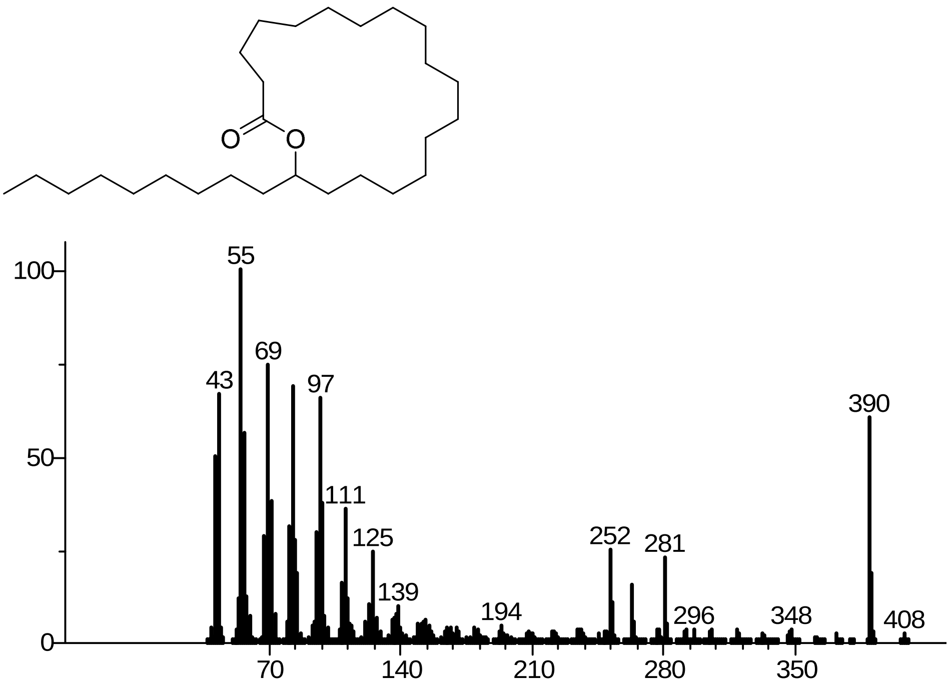

Figure S16. Mass spectrum and structure of heptacosan-18-olide (**1**).

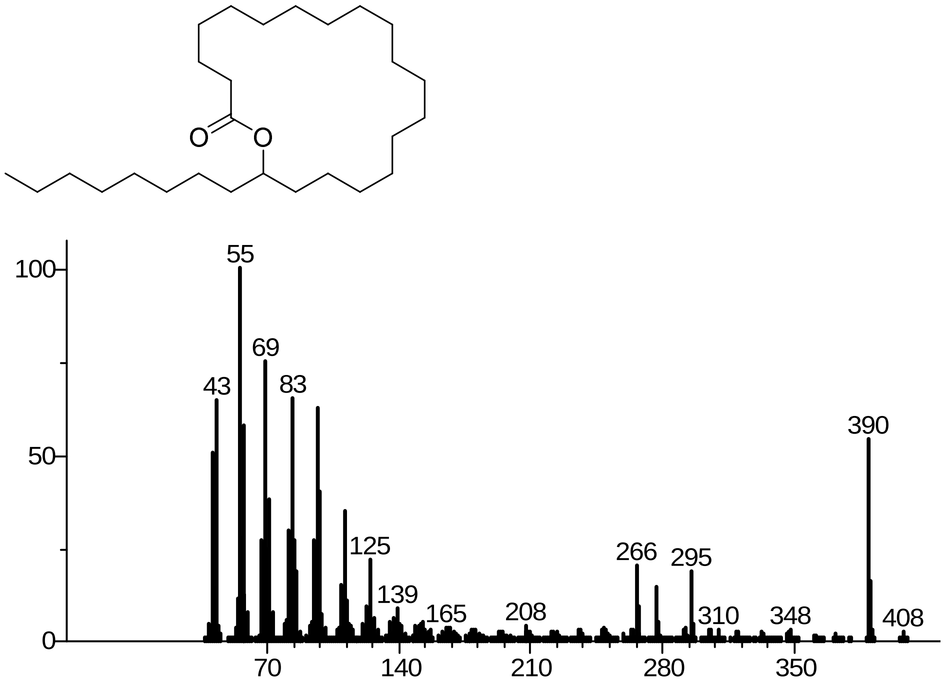

Figure S17. Mass spectrum and structure of heptacosan-19-olide.

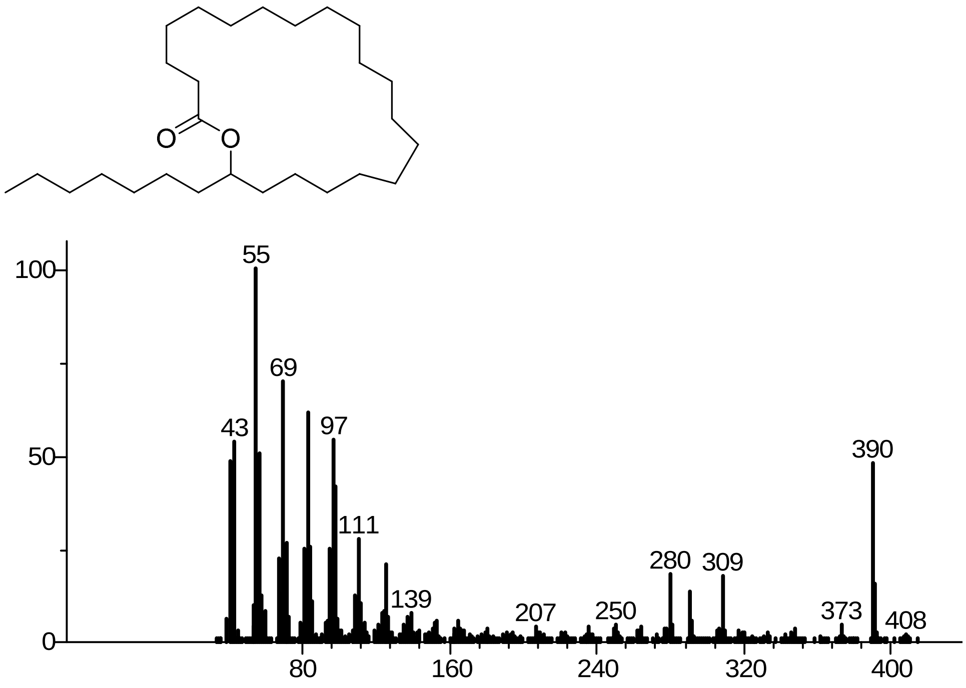

Figure S18. Mass spectrum and structure of heptacosan-20-olide.

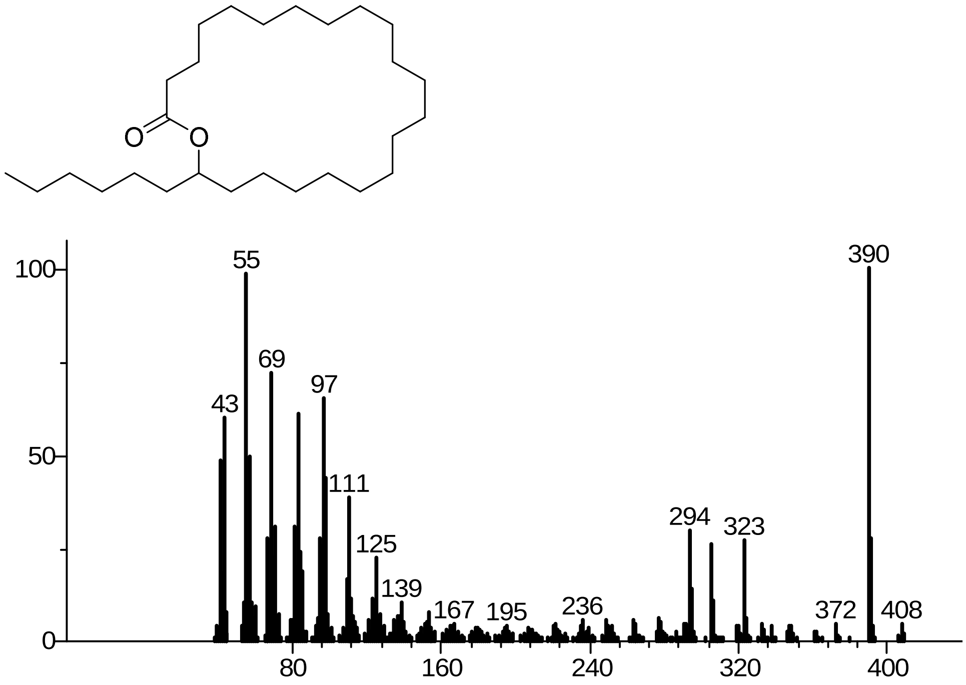

Figure S19. Mass spectrum and structure of heptacosan-21-olide.

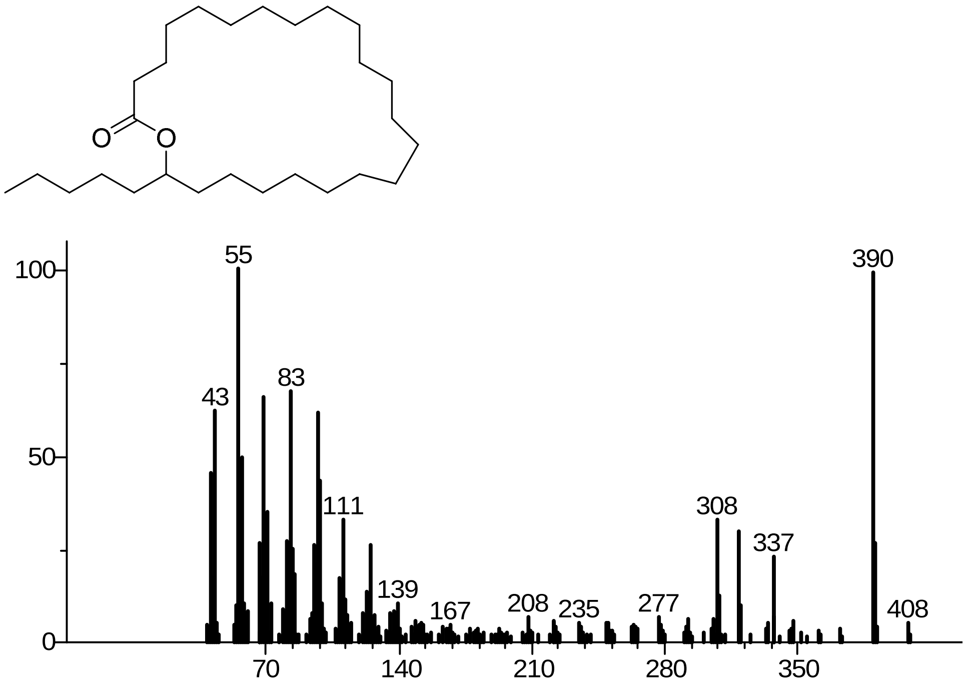

Figure S20. Mass spectrum and structure of heptacosan-22-olide.

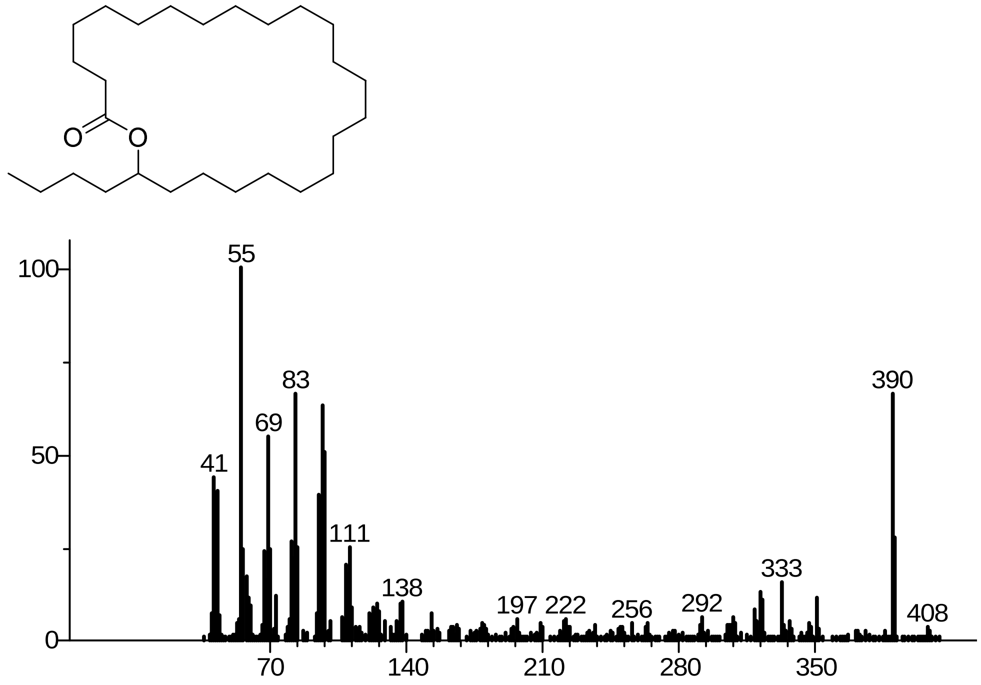

Figure S21. Mass spectrum and structure of heptacosan-23-olide.

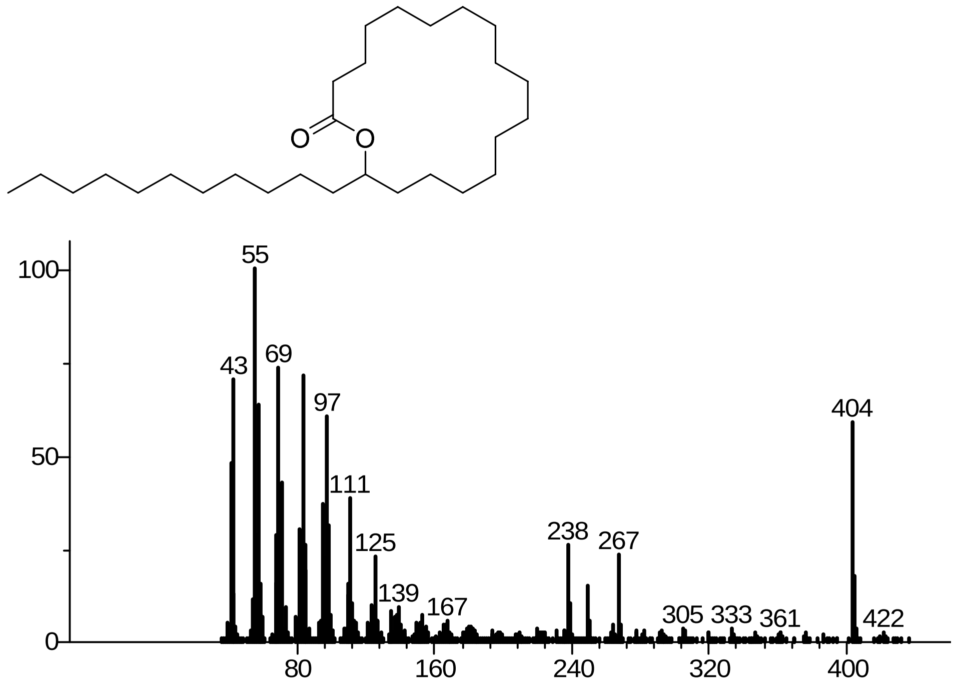

Figure S22. Mass spectrum and structure of octacosan-17-olide.

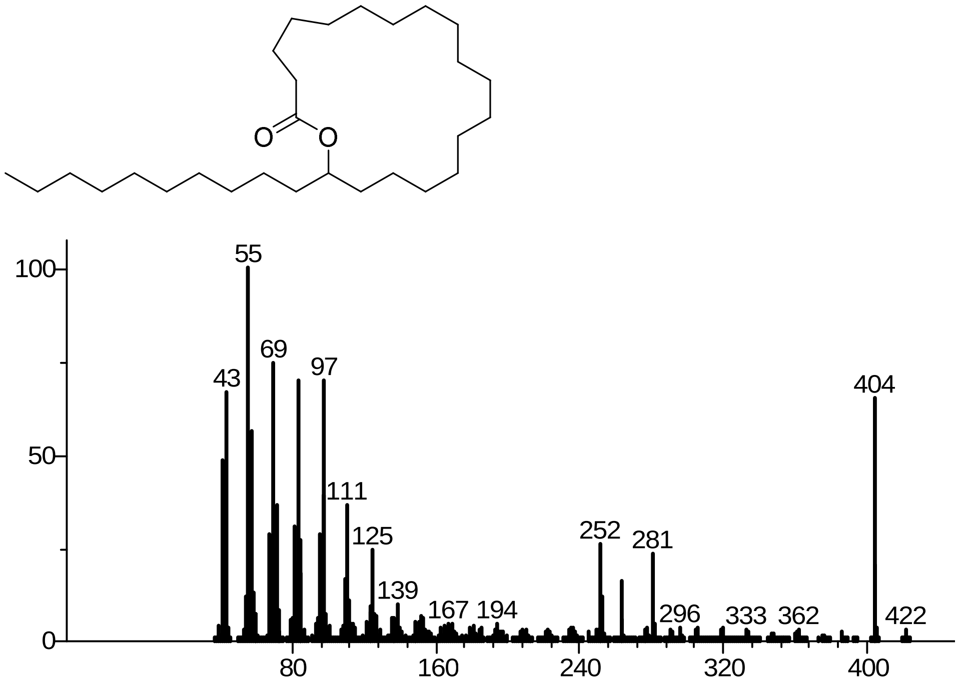

Figure S23. Mass spectrum and structure of octacosan-18-olide (**9**).

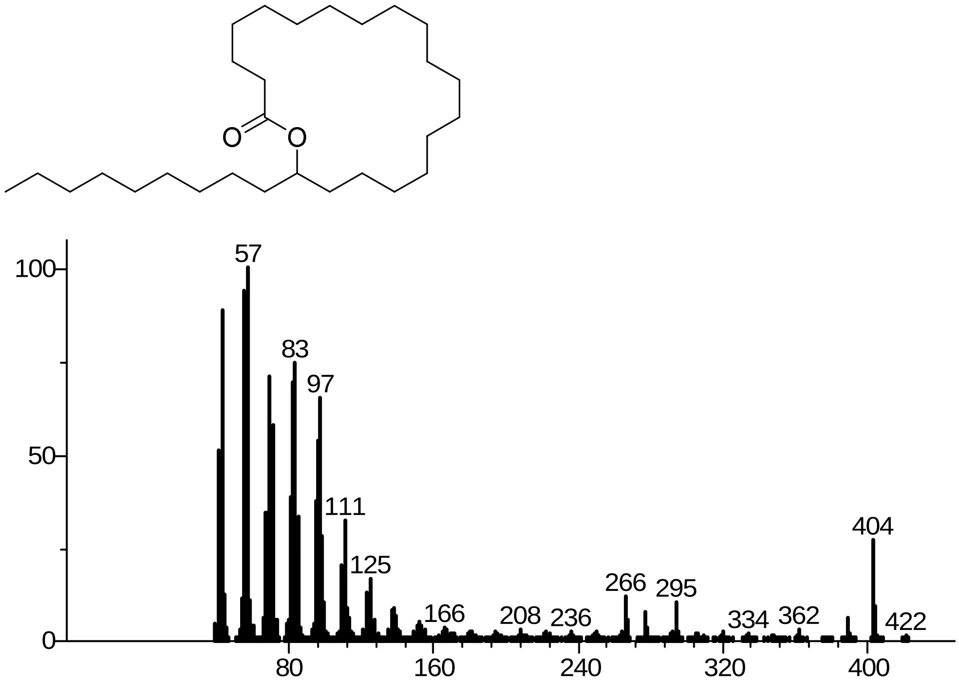

Figure S24. Mass spectrum and structure of octacosan-19-olide.

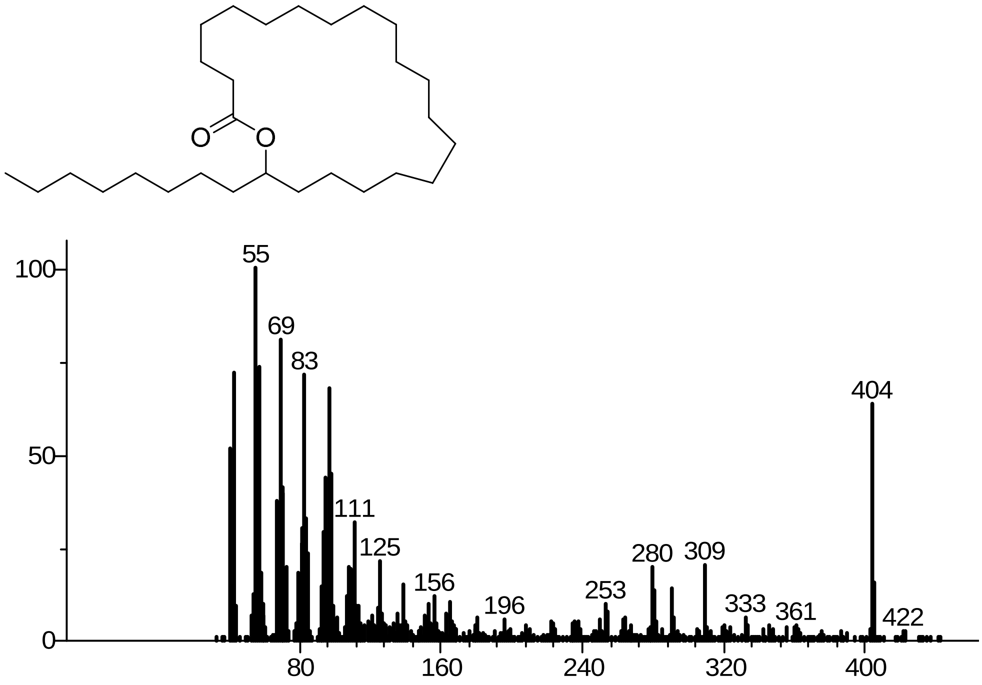

Figure S25. Mass spectrum and structure of octacosan-20-olide.

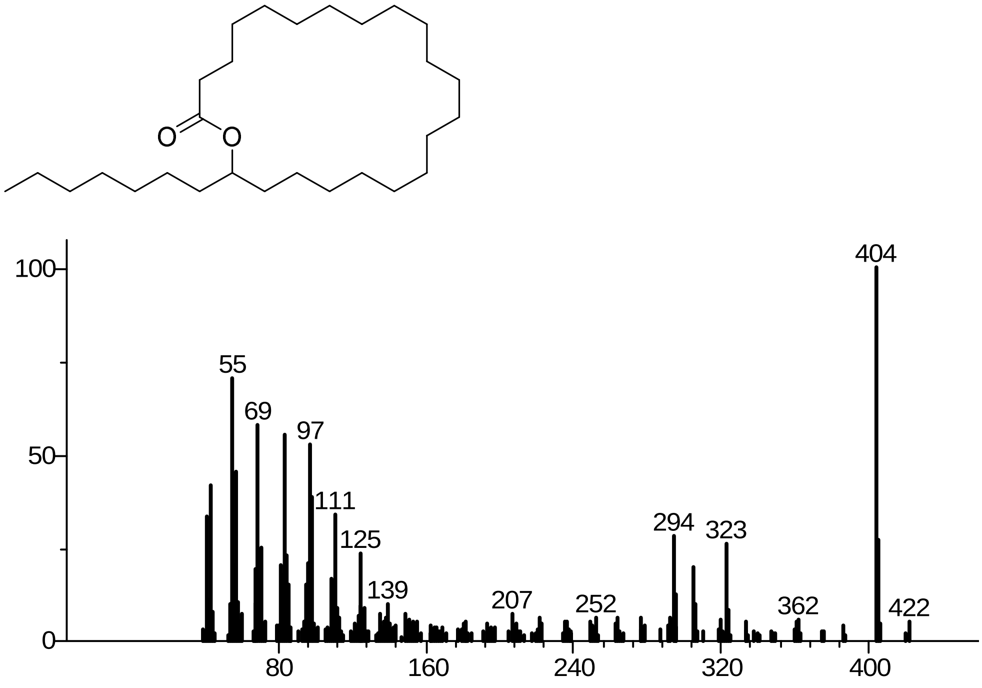

Figure S26. Mass spectrum and structure of octacosan-21-olide.

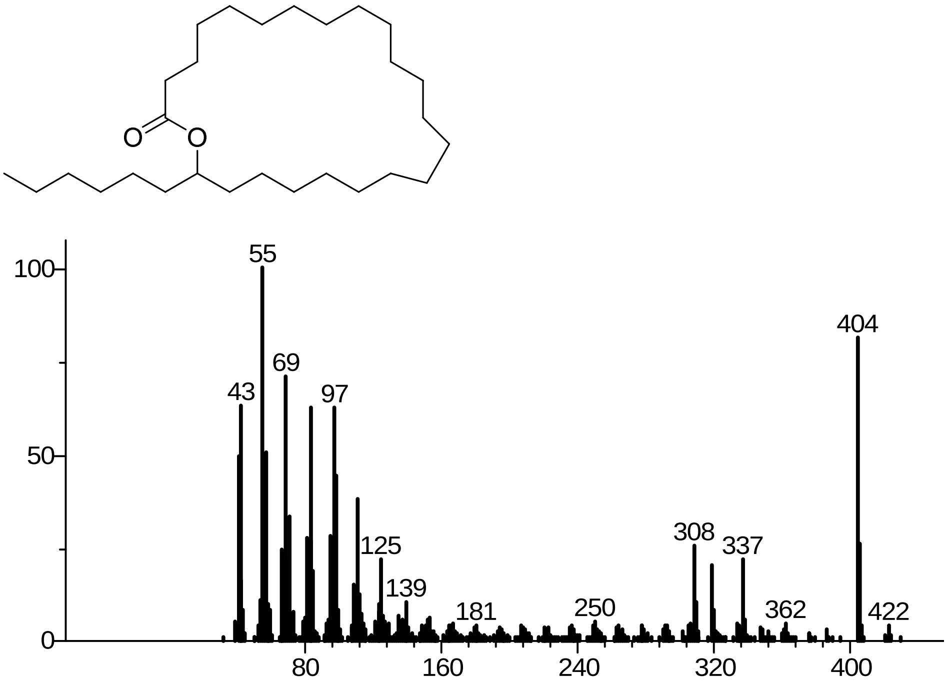

Figure S27. Mass spectrum and structure of octacosan-22-olide (**11**).

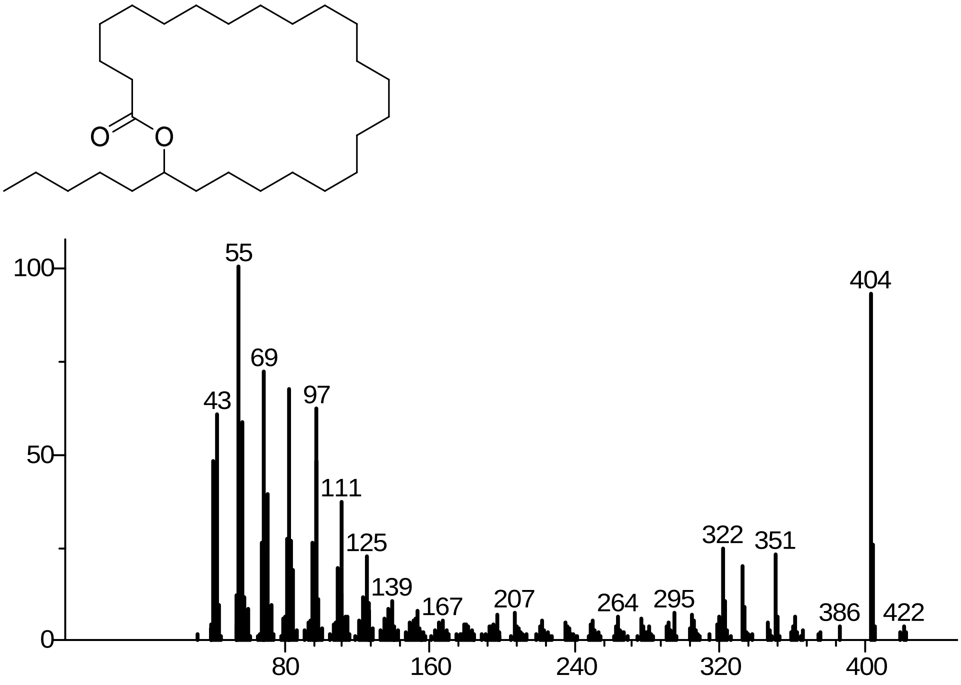

Figure S28. Mass spectrum and structure of octacosan-23-olide.

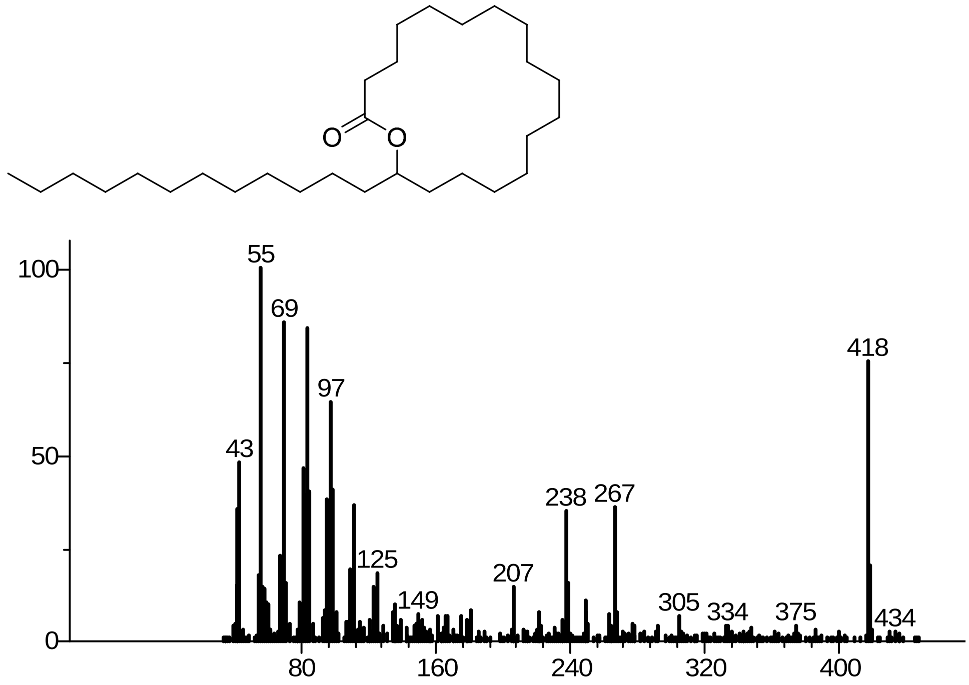

Figure S29. Mass spectrum and structure of nonacosan-17-olide.

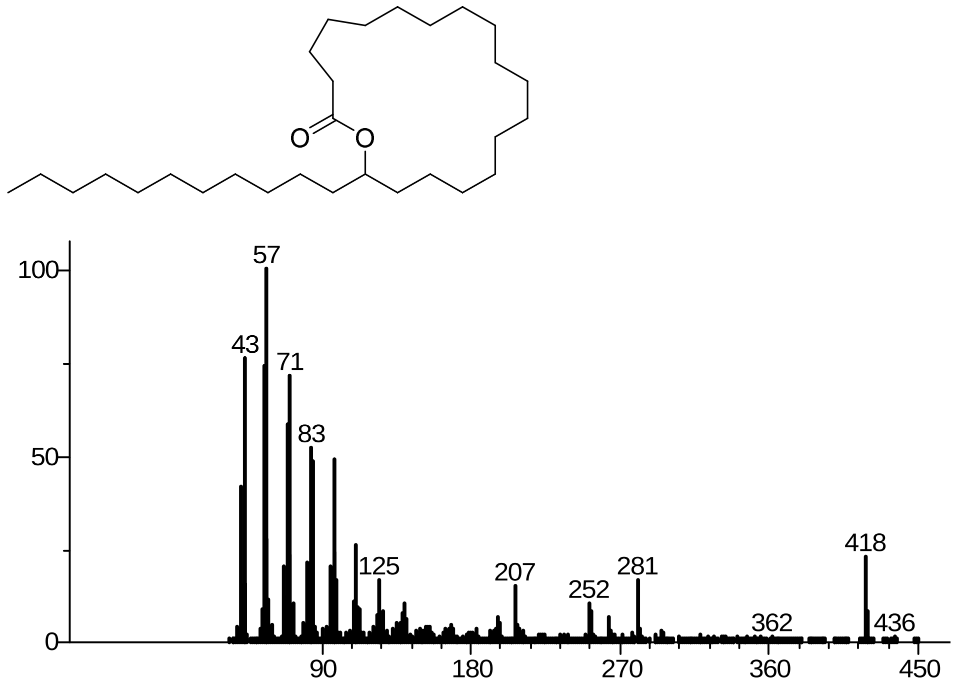

Figure S30. Mass spectrum and structure of nonacosan-18-olide.

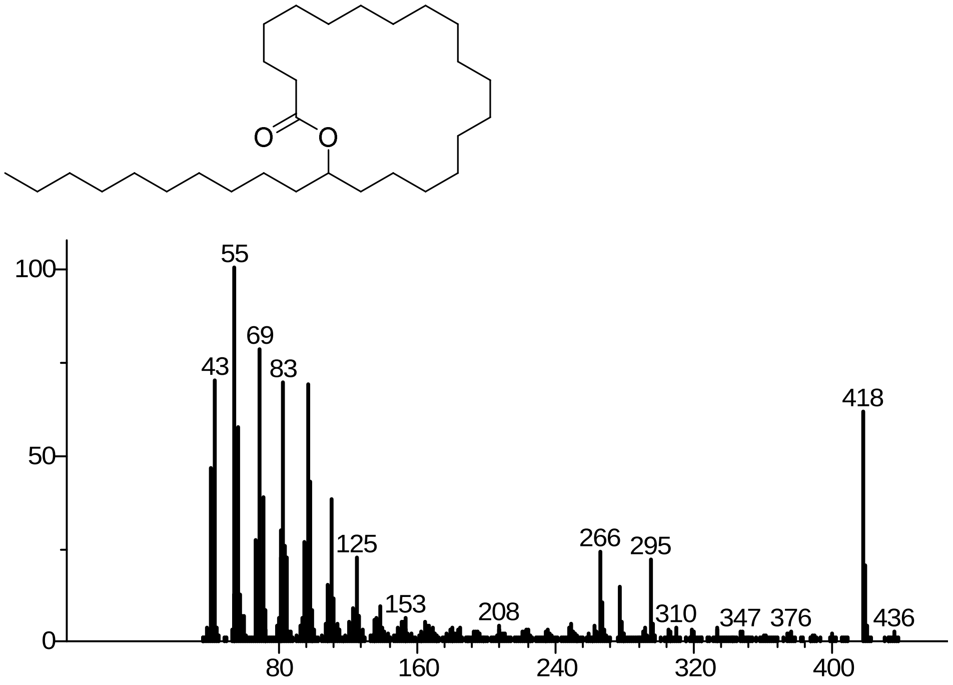

Figure S31. Mass spectrum and structure of nonacosan-19-olide.

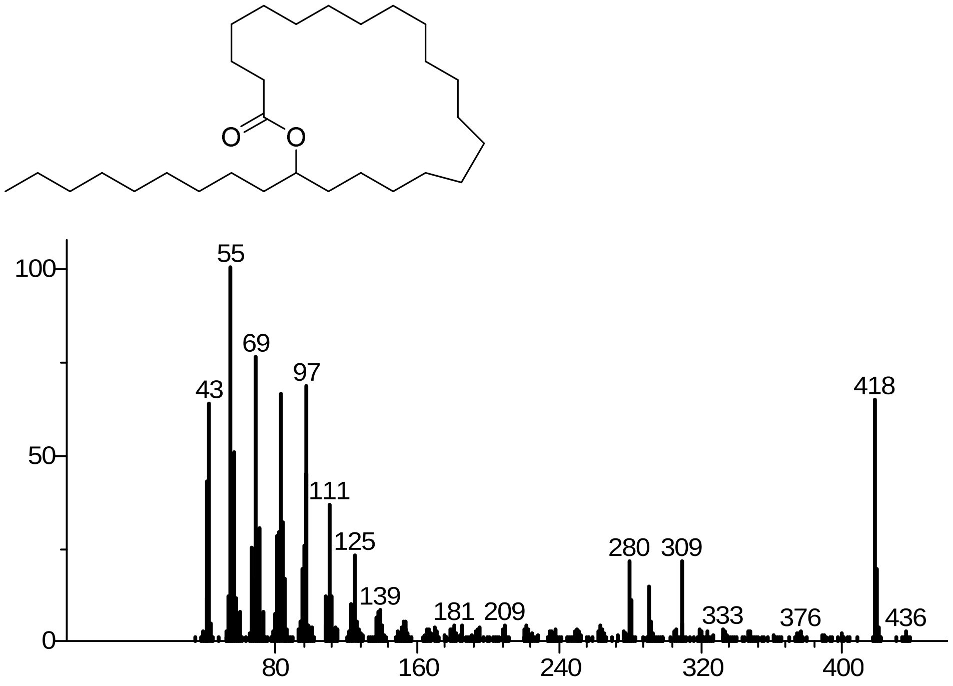

Figure S32. Mass spectrum and structure of nonacosan-20-olide.

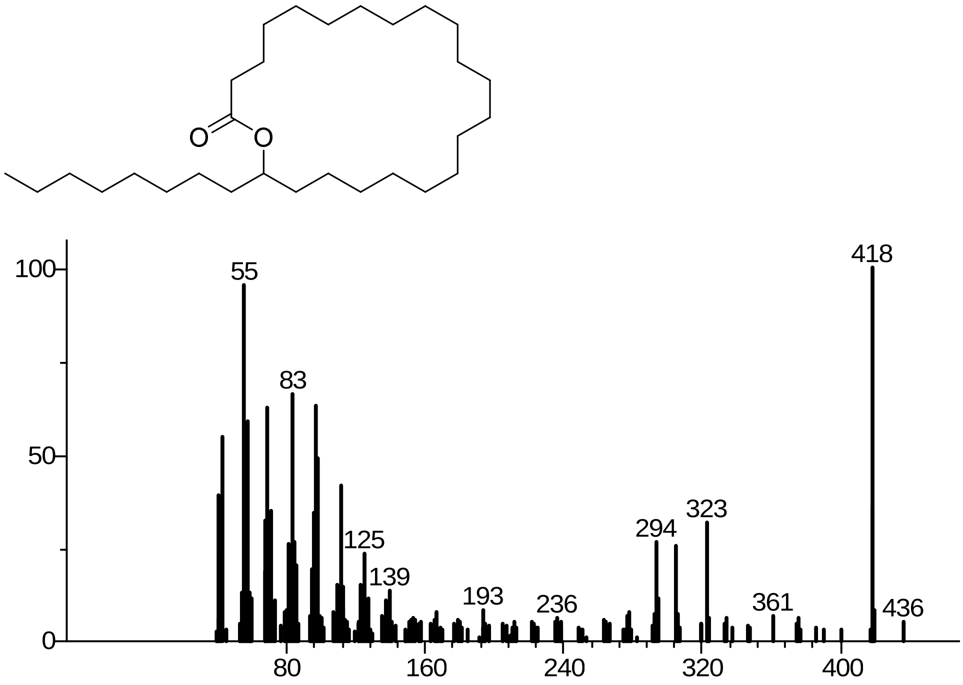

Figure S33. Mass spectrum and structure of nonacosan-21-olide.

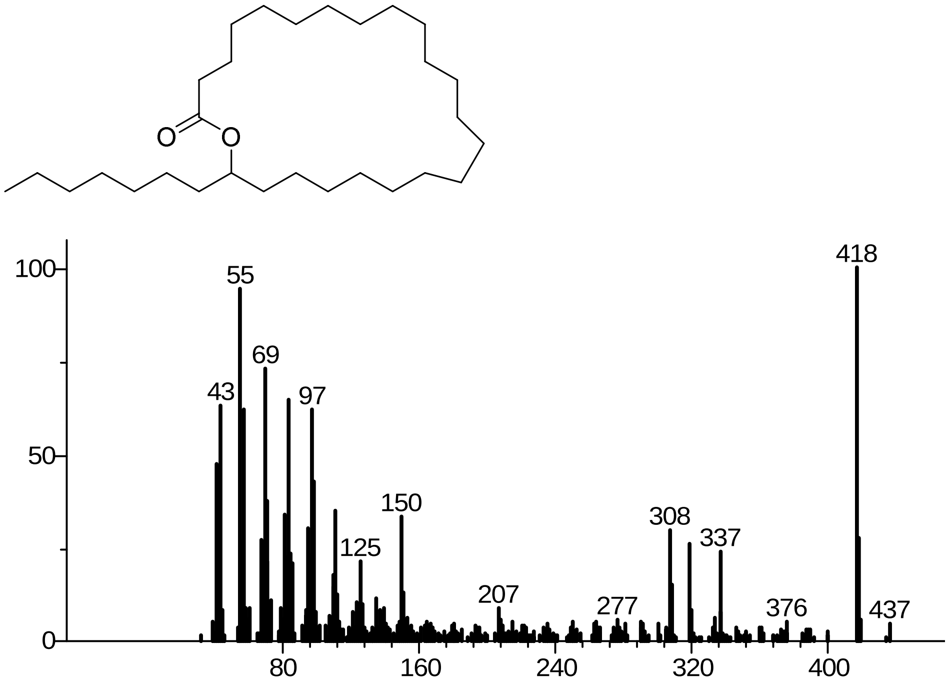

Figure S34. Mass spectrum and structure of nonacosan-22-olide.

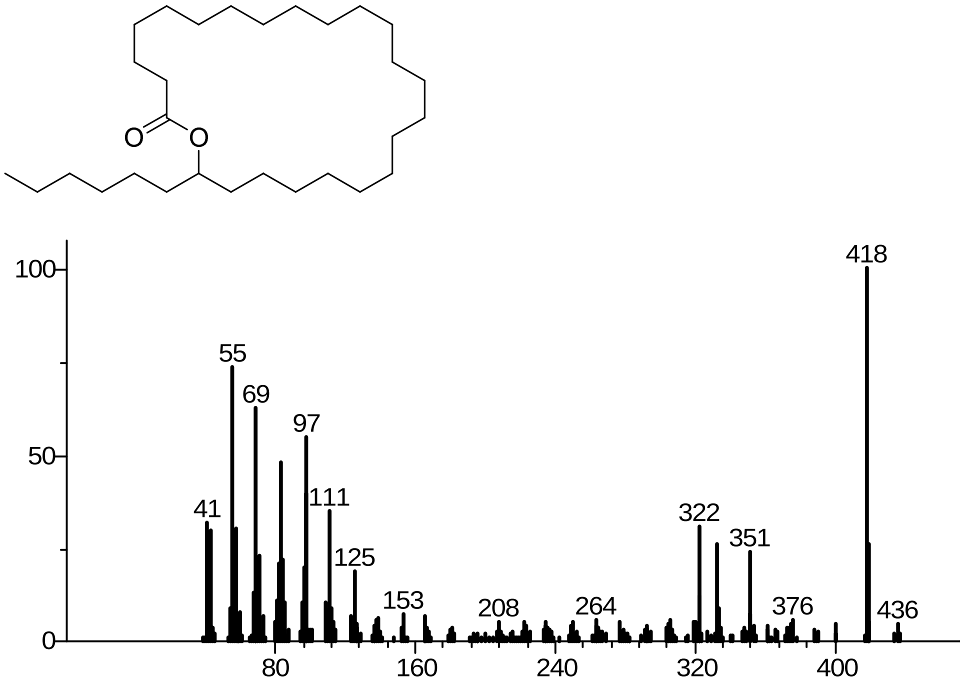

Figure S35. Mass spectrum and structure of nonacosan-23-olide (**10**).

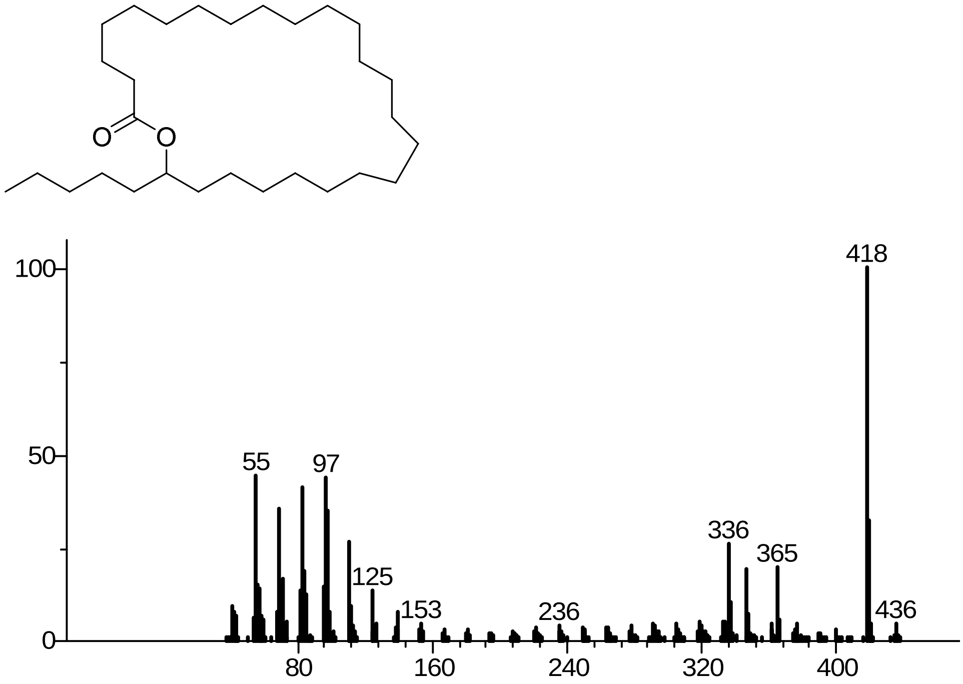

Figure S36. Mass spectrum and structure of nonacosan-24-olide (**13**).

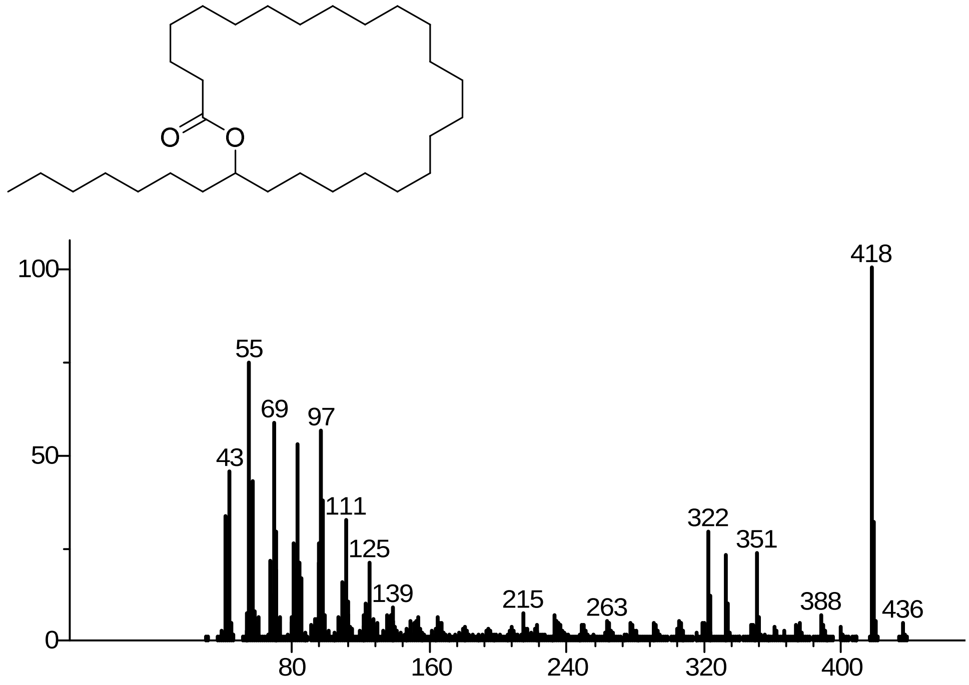

Figure S37. Mass spectrum and structure of triacontan-23-olide (**12**).

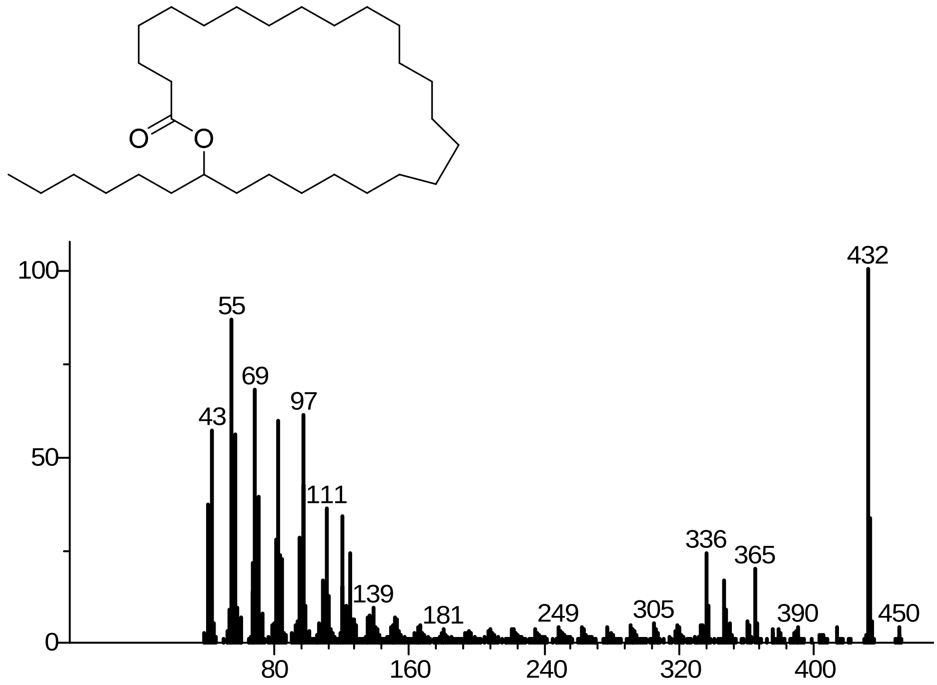

Figure S38. Mass spectrum and structure of triacontan-24-olide (**14**).

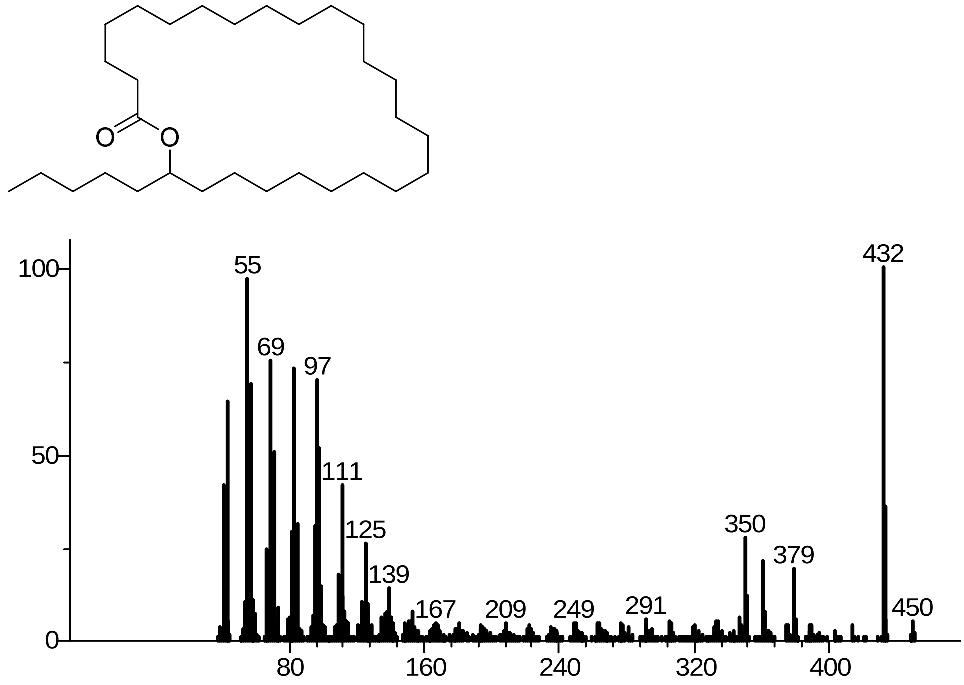

Figure S39. Mass spectrum and structure of triacontan-25-olide.

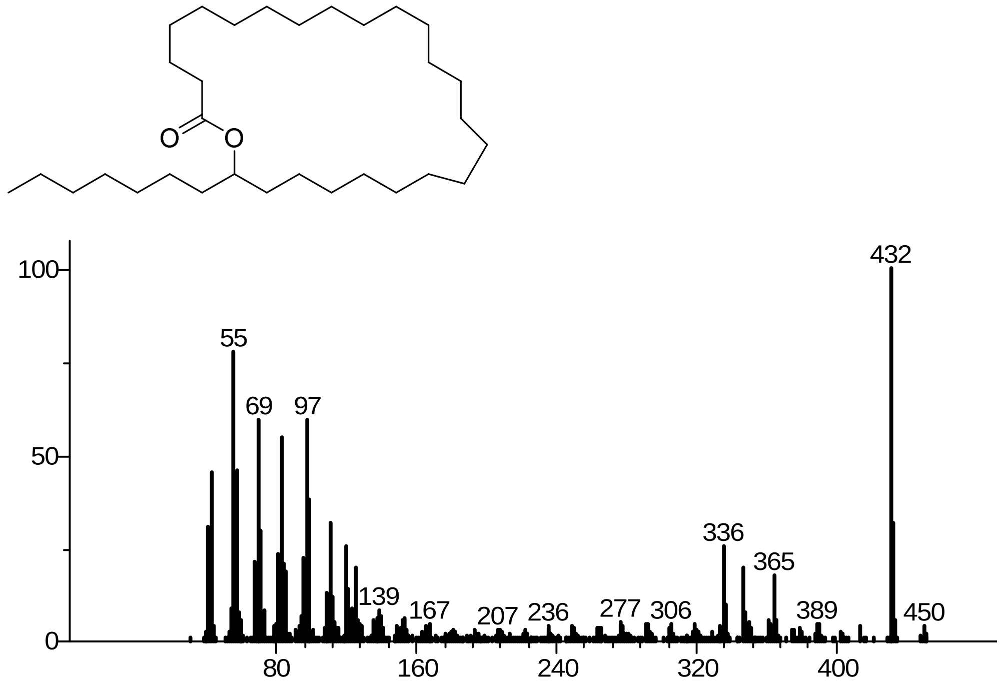

Figure S40. Mass spectrum and structure of hentriacontan-24-olide.

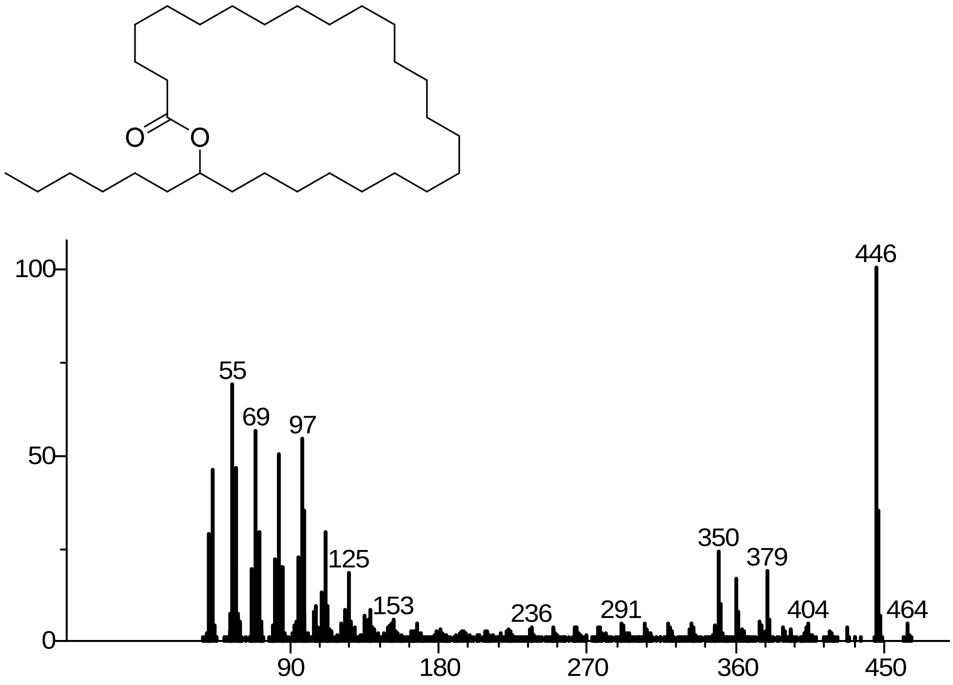

Figure S41. Mass spectrum and structure of hentriacontan-25-olide.

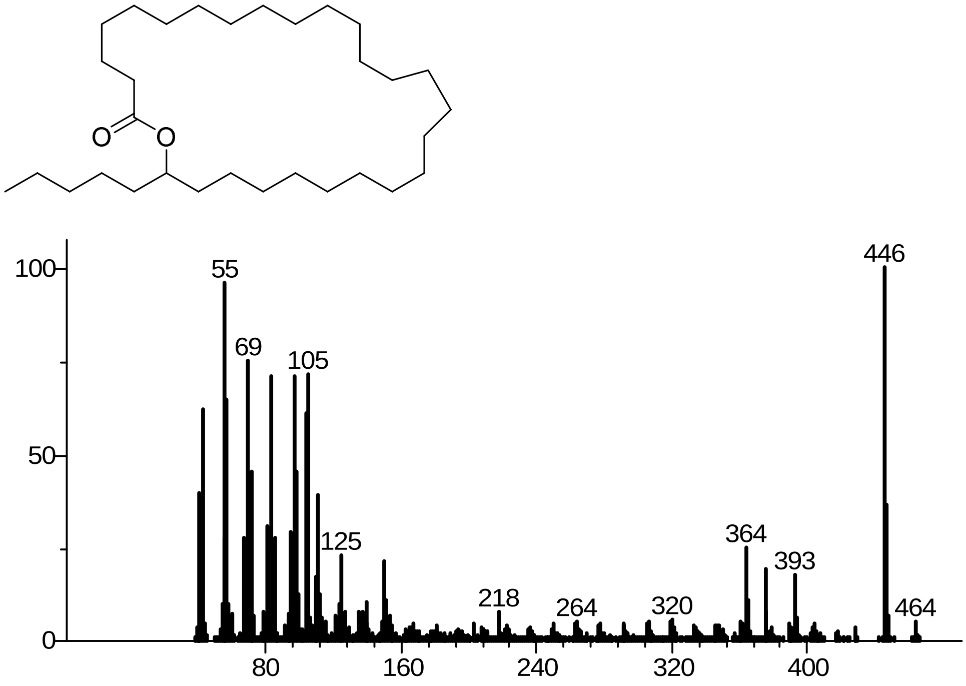

Figure S42. Mass spectrum and structure of hentriacontan-26-olide.

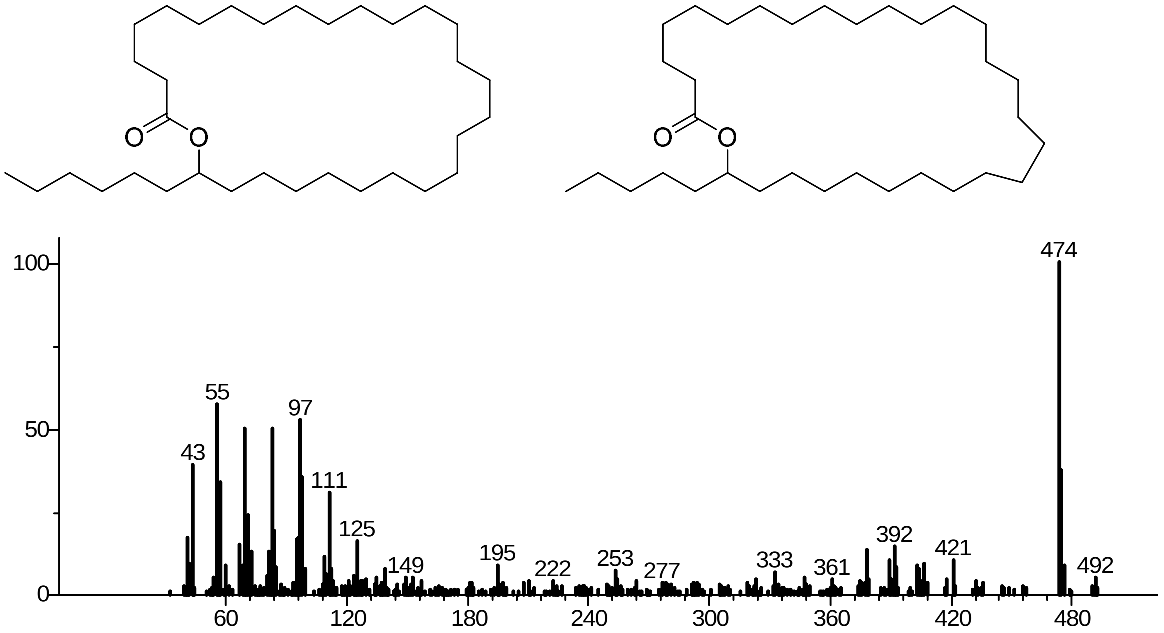

Figure S43. Mass spectrum and structures of a mixture of tritriacontan-27-olide and tritriacontan-28-olide.

Representative mass spectra shown in the Figures in this article will be published in the open-access database MACE in downloadable format[1,2] in release mace_r09. The current release mace_r08 can be found at https://doi.org/10.24355/dbbs.084-202508211140-0

3 High-Resolution EI-Orbitrap Mass Spectra of Compounds 1 and 2

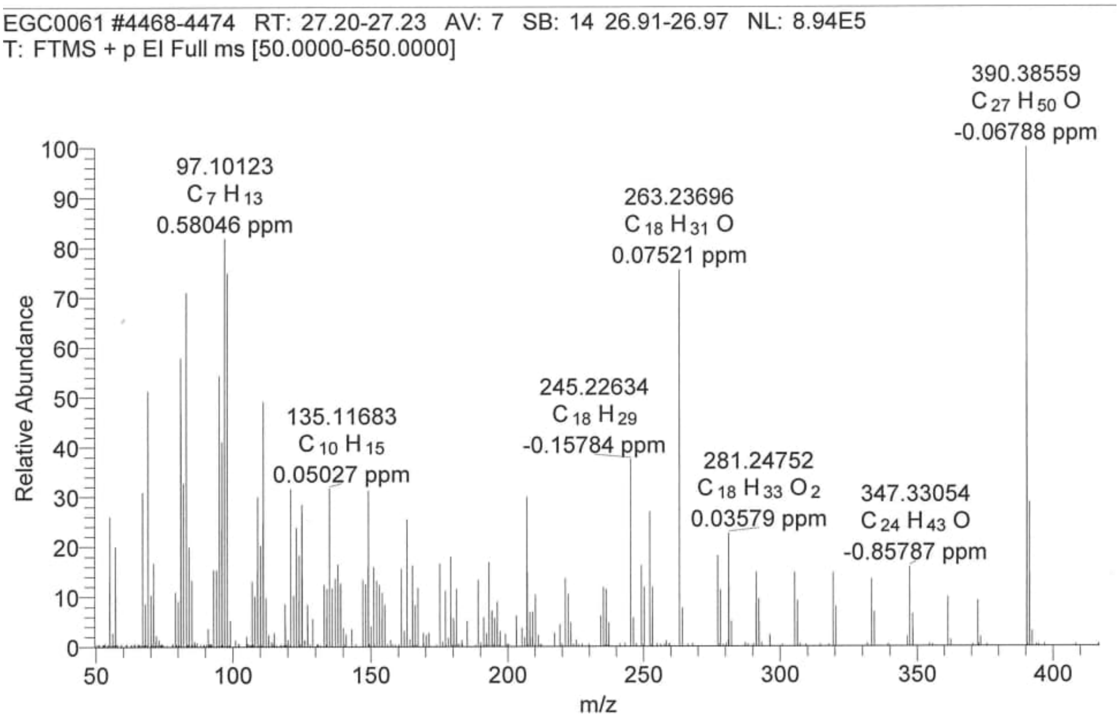

Figure S44. HR-Mass spectrum of heptacosan-18-olide (**1**).

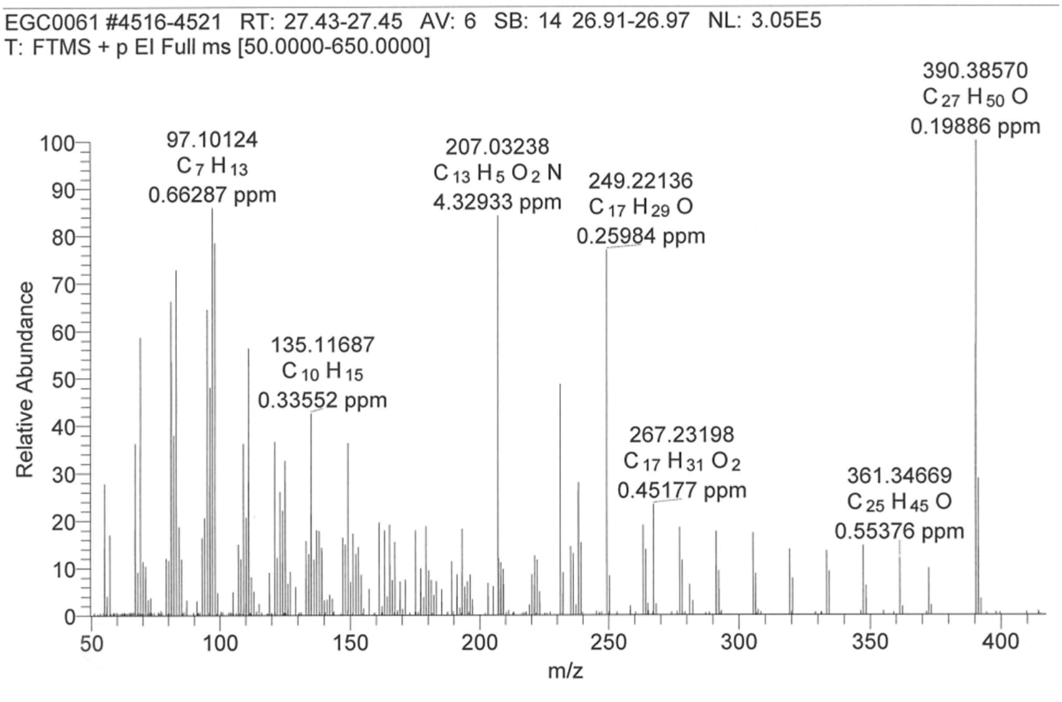

Figure S45. HR-Mass spectrum of heptacosan-17-olide (**2**). The ion *m*/*z* 207 arose from an impurity.

4 Total ion chromatograms

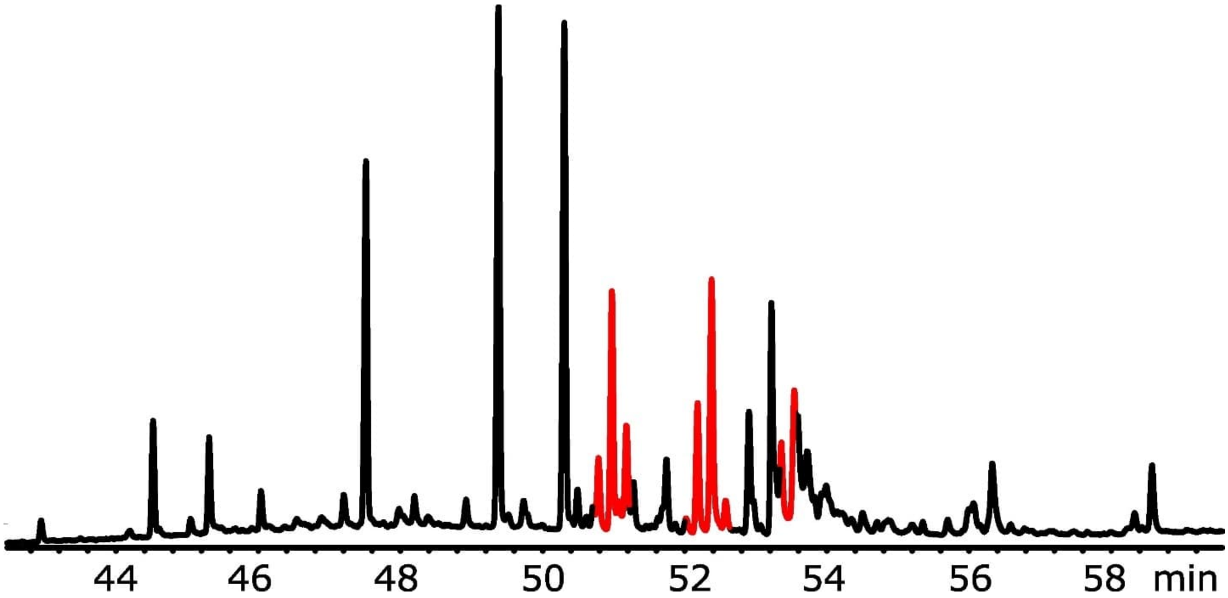

Figure S46. TIC of a wing extract of a male *Eueides vibilia* stored over several years in an envelope. Peaks corresponding to macrocyclic lactones are shown in red.

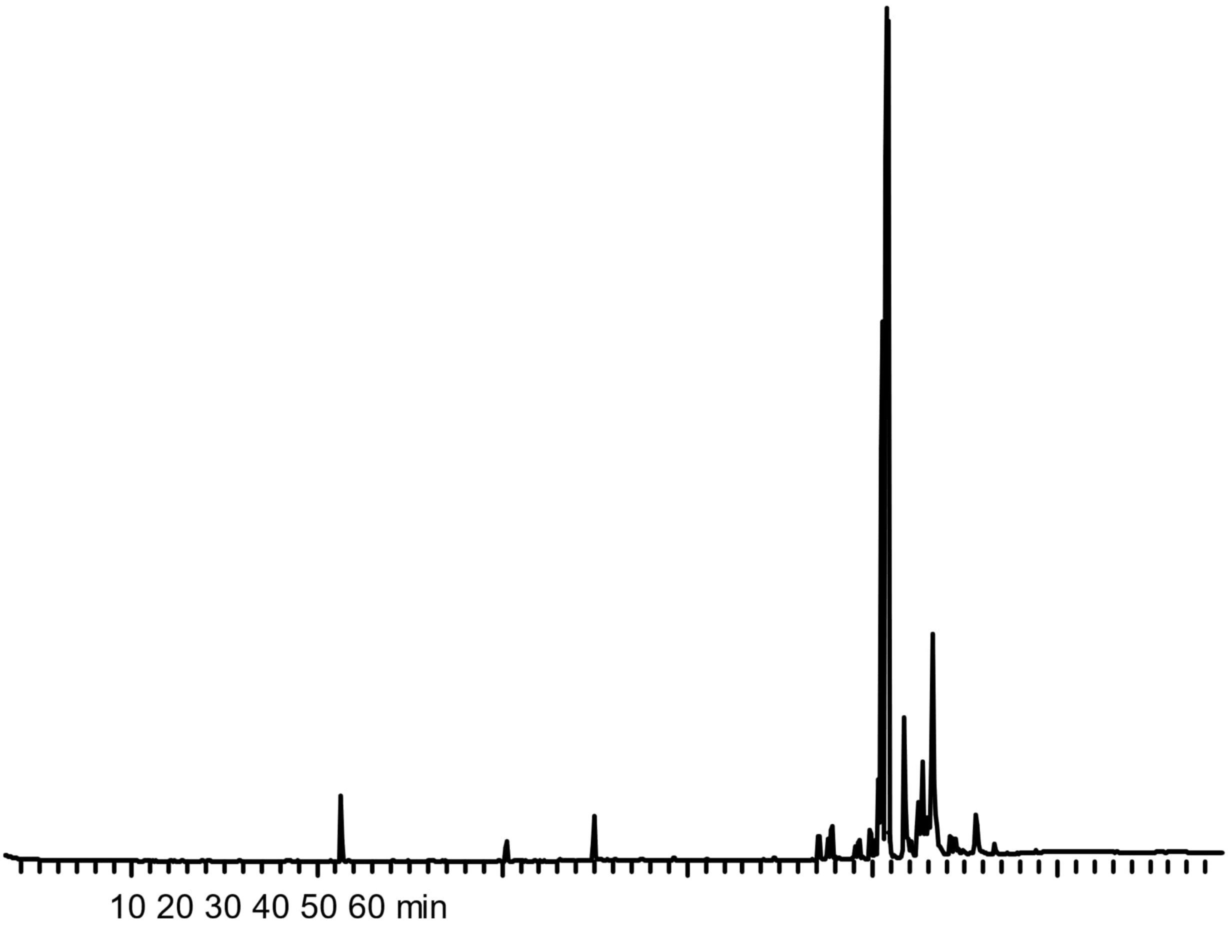

Figure S47. TIC of an extract of wings of a male of *Eueides tales* from the wild. The portion starting from minute 45 is shown in the main manuscript (Figure 1a), including peak assignments. The peak at 21 min is triacetin, the one at 30 min is the internal standard, 2-tetradecylacetate, and the peak at 35 min is palmitic acid.

5 Phylogeny of *Eueides* butterflies

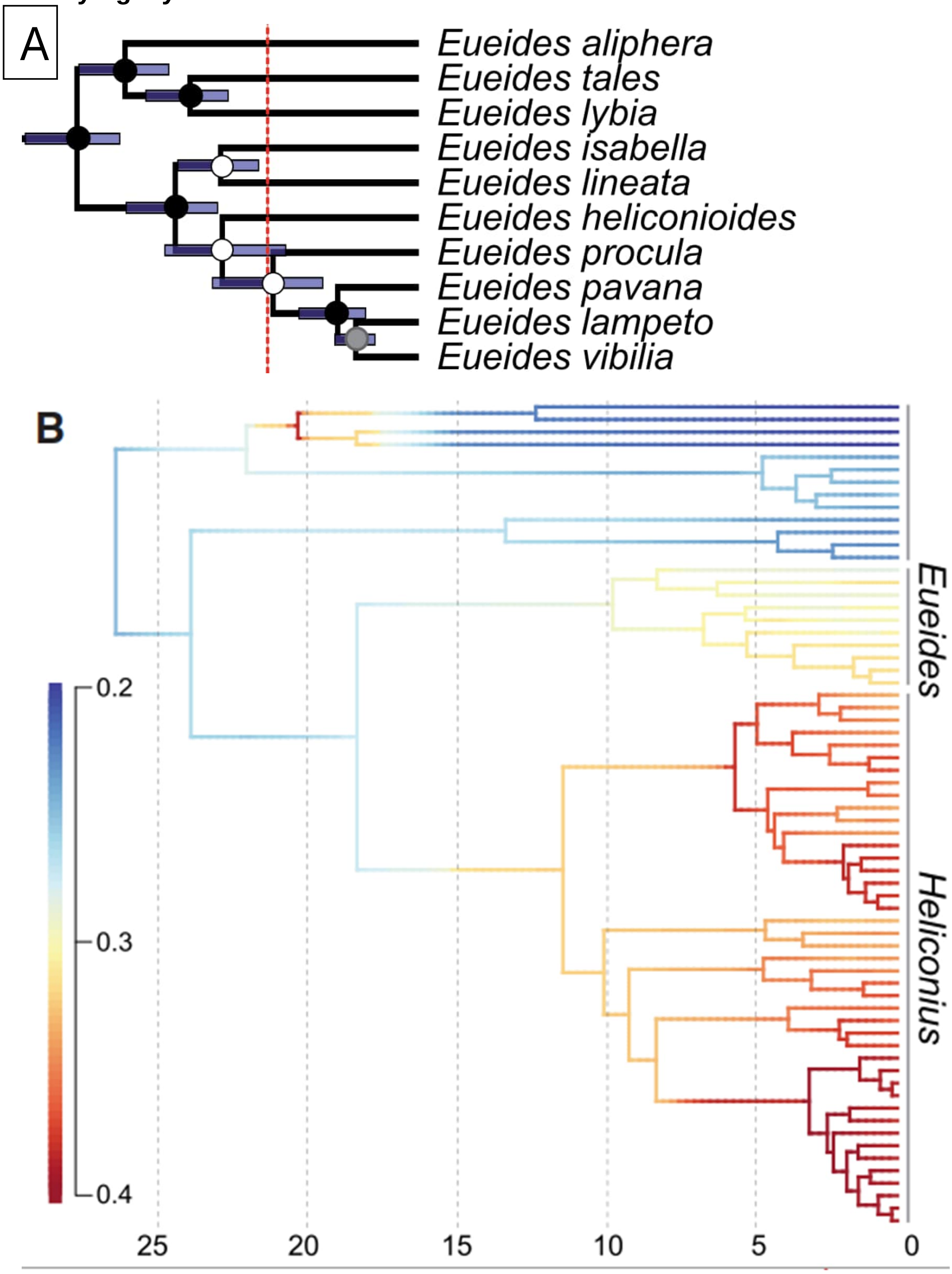

Figure S48. Phylogeny of *Eueides* (A) and general tree of the Heliconiinae (B) according to Kozak et al.[3] Snippets from his Figure 1 showing the full phylogenetic tree of the Heliconiinae.

6 NMR Spectra

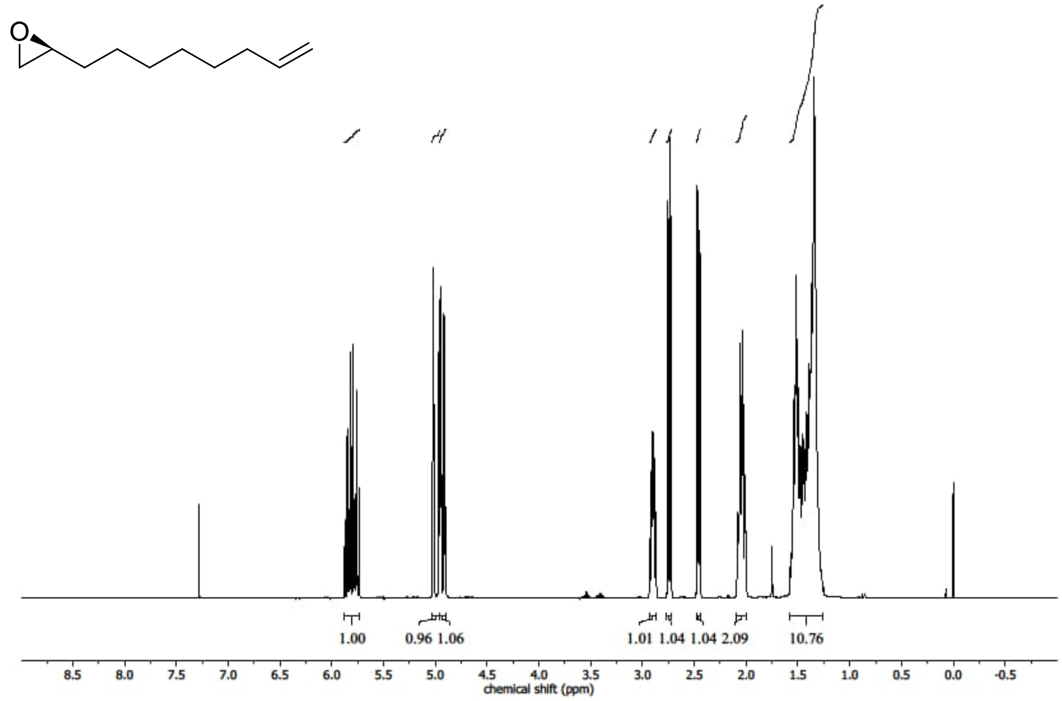

Figure S49. 300 MHz ^{1}H NMR spectrum of (*S*)-2-(oct-7-en-1-yl)oxirane.

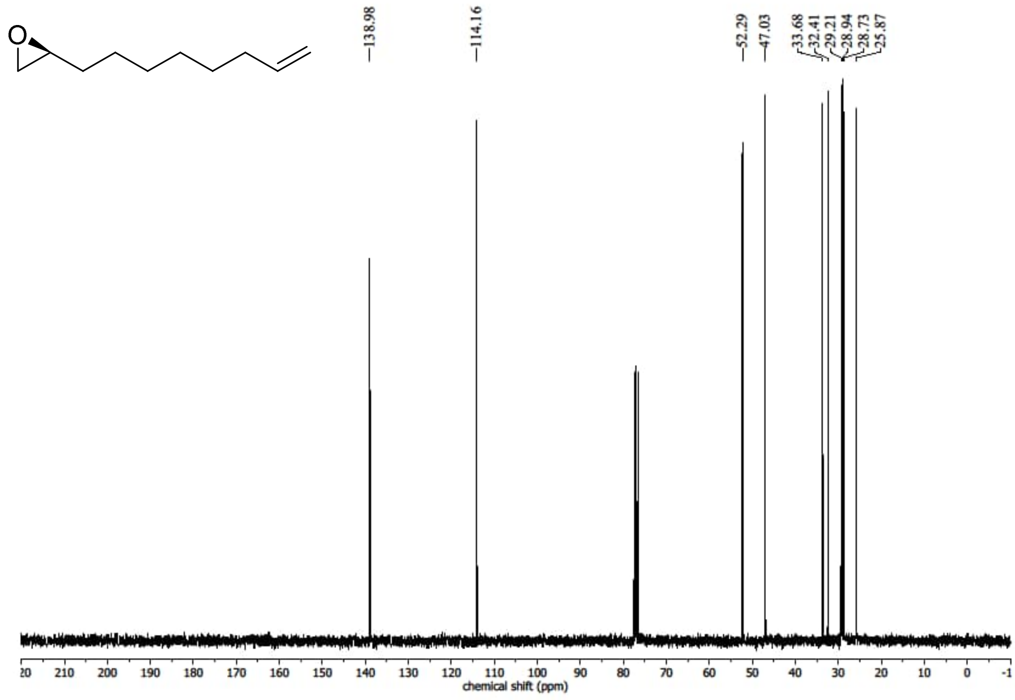

Figure S50. 75 MHz ^{13}C NMR spectrum of (*S*)-2-(oct-7-en-1-yl)oxirane.

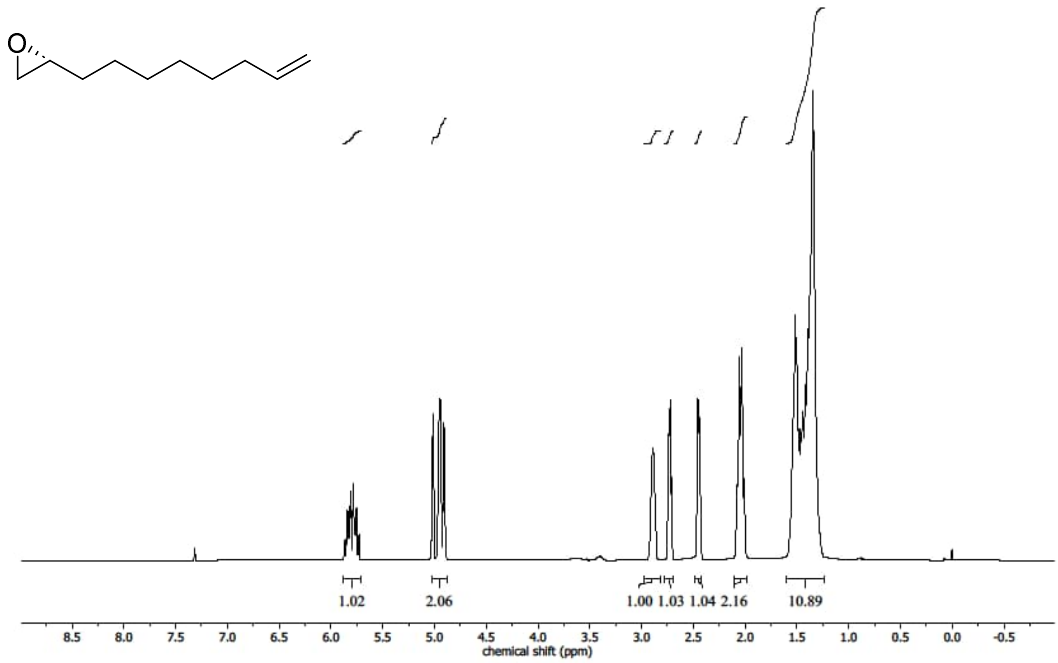

Figure S51. 300 MHz ^{1}H NMR spectrum of (*R*)-2-(oct-7-en-1-yl)oxirand.

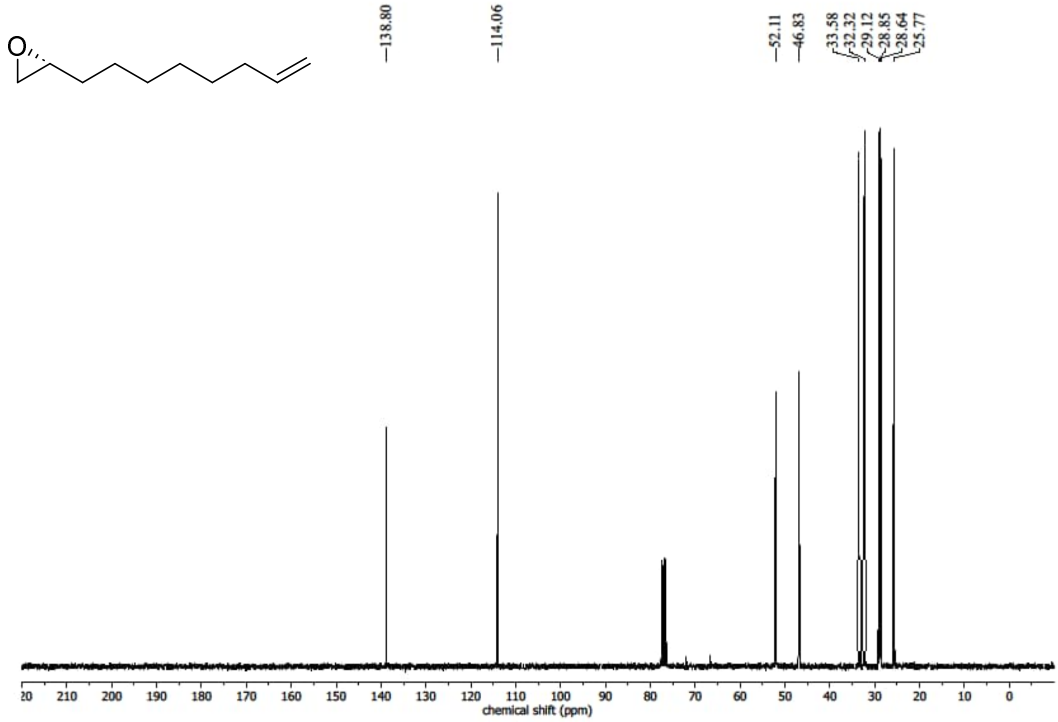

Figure S52. 75 MHz ^{13}C NMR spectrum of (*R)*-2-(oct-7-en-1-yl)oxirane.

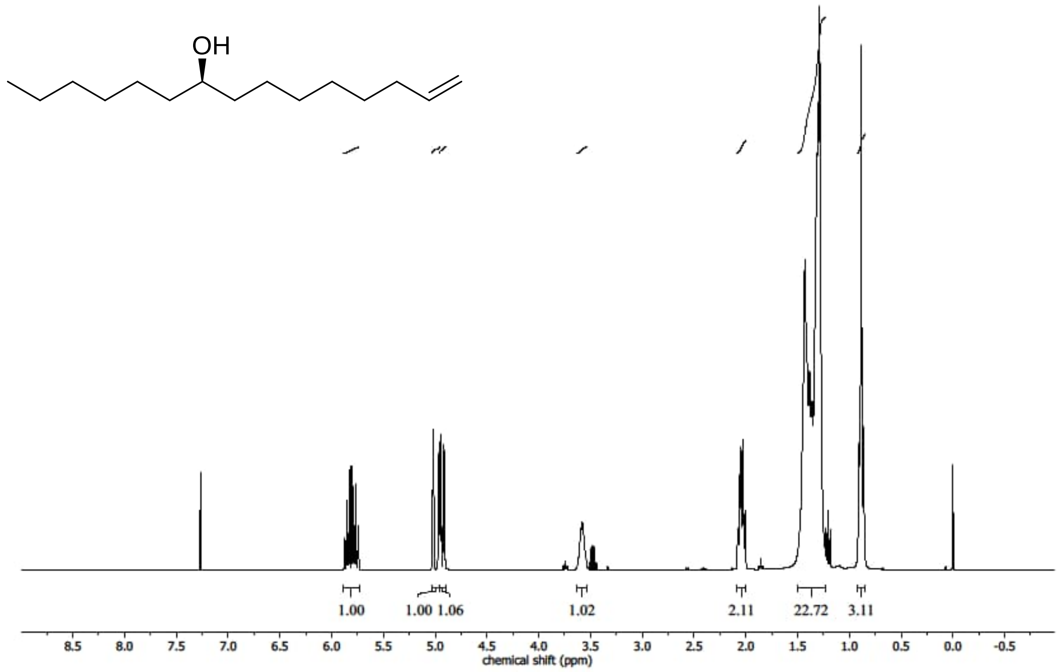

Figure S53. 300 MHz ^{1}H NMR spectrum of (*R*)-pentadec-14-en-7-ol.

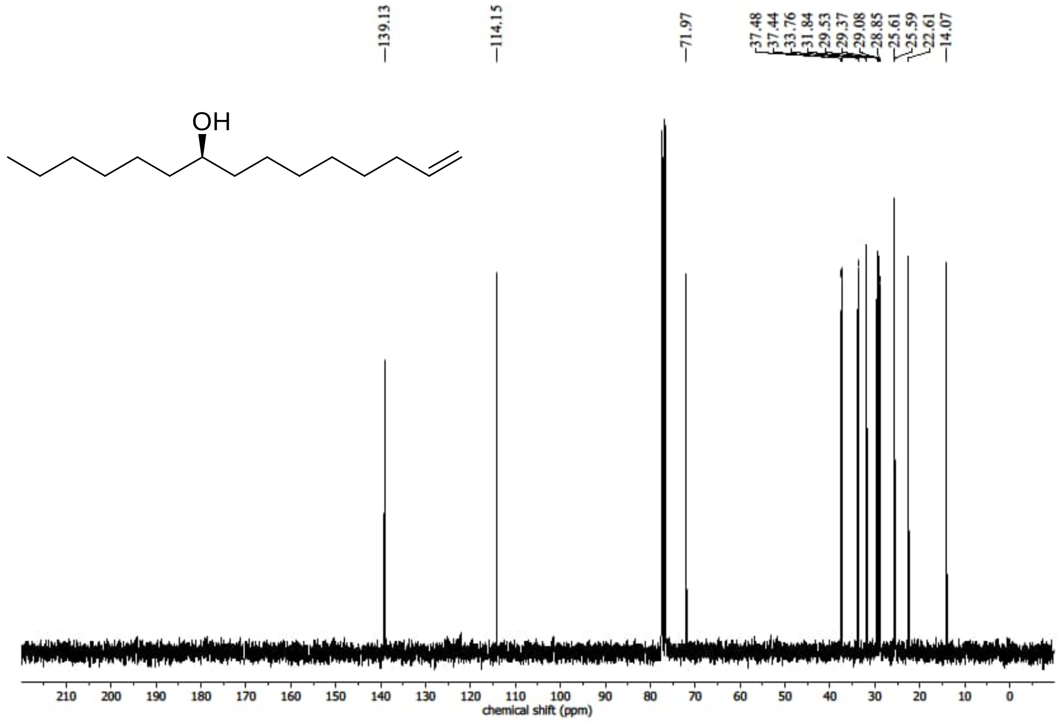

Figure S54. 75 MHz ^{13}C NMR spectrum of (*R*)-pentadec-14-en-7-ol.

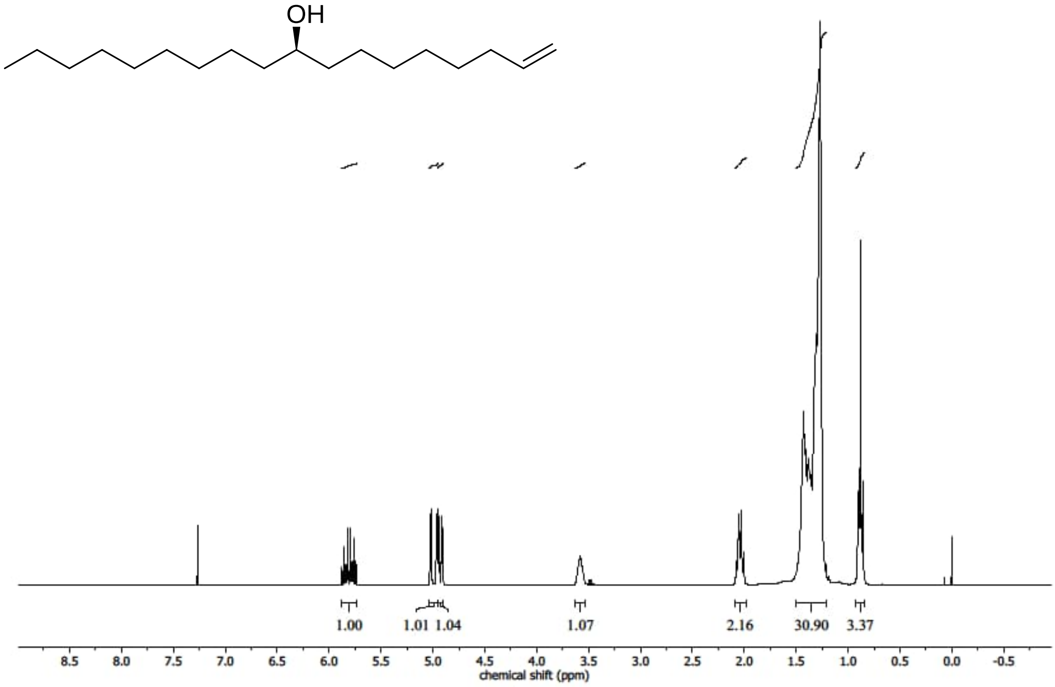

Figure S55. 300 MHz ^{1}H NMR spectrum of (*R*)-octadec-1-en-9-ol.

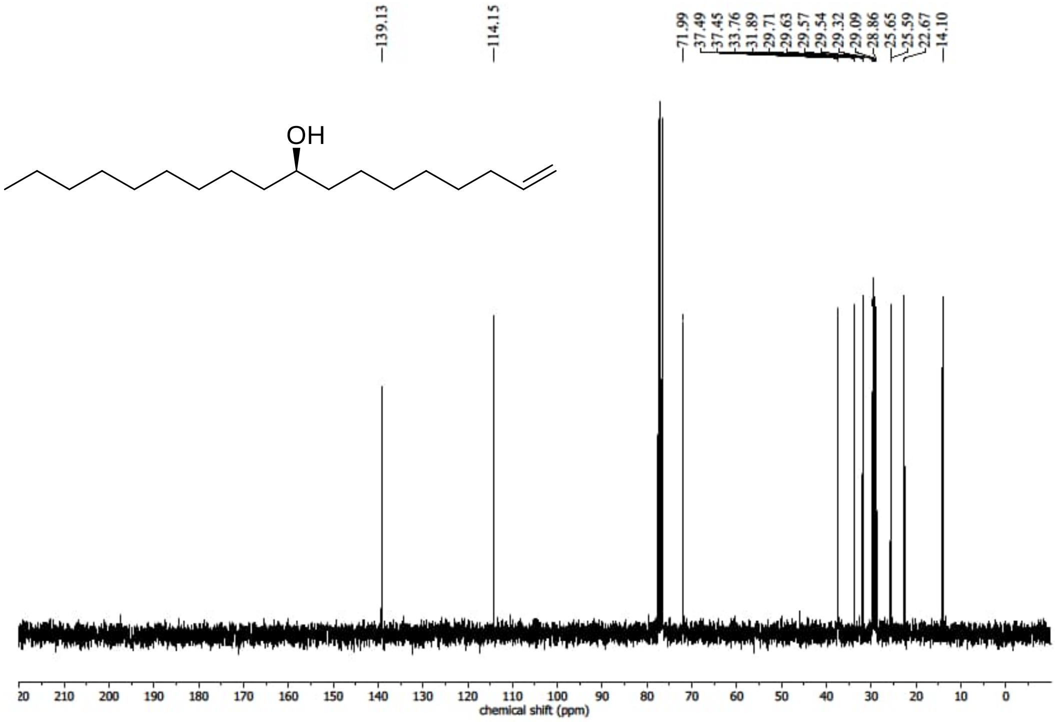

Figure S56. 75 MHz ^{13}C NMR spectrum of (*R*)-octadec-1-en-9-ol.

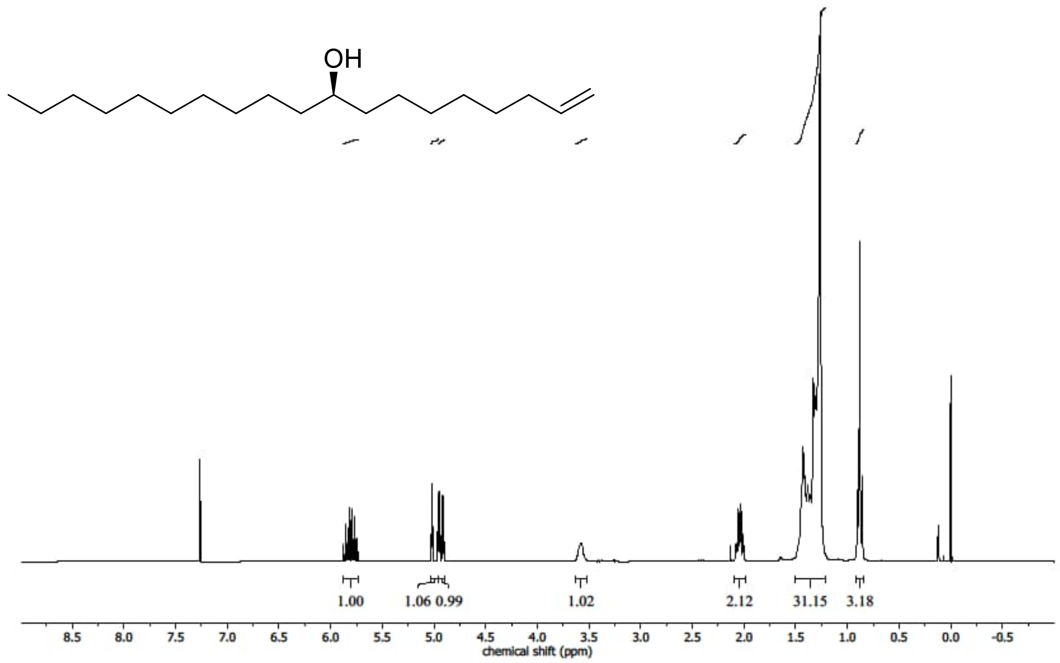

Figure S57. 300 MHz ^{1}H NMR spectrum of (*R*)-nonadec-1-en-9-ol .

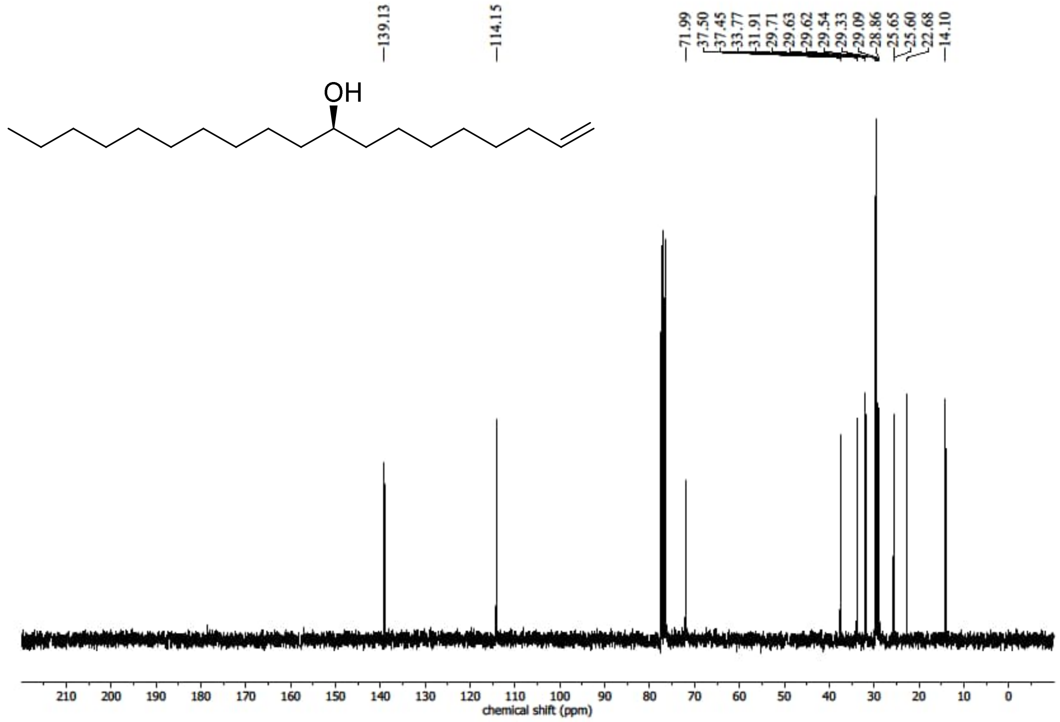

Figure S58. 75 MHz ^{13}C NMR spectrum of (*R*)-nonadec-1-en-9-ol.

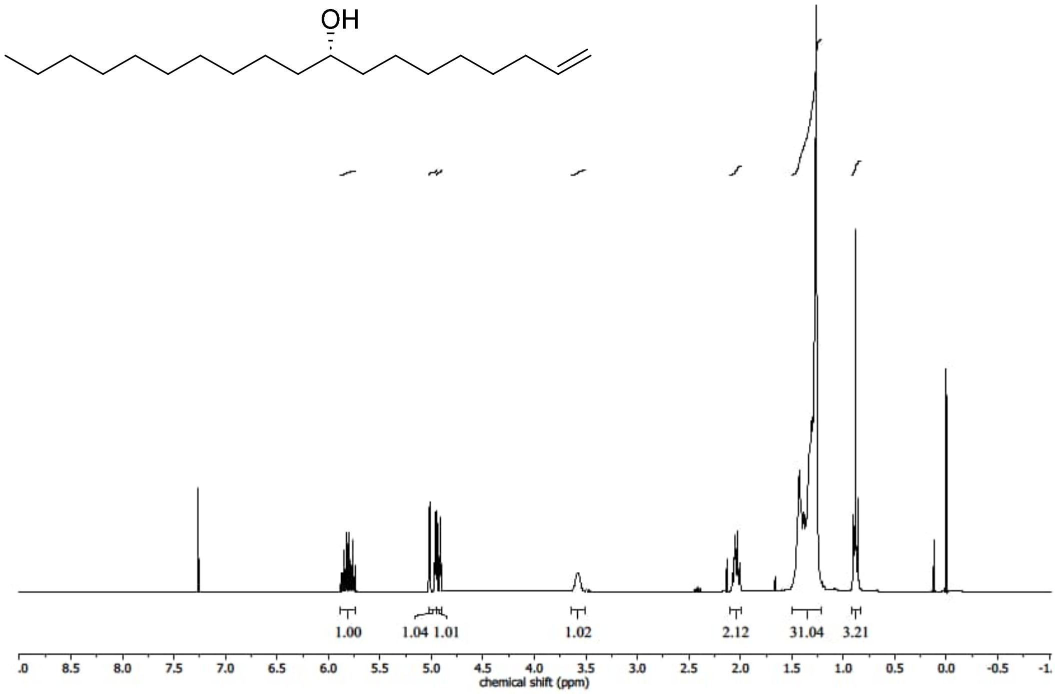

Figure S59. 300 MHz ^{1}H NMR spectrum of (*S*)-nonadec-1-en-9-ol .

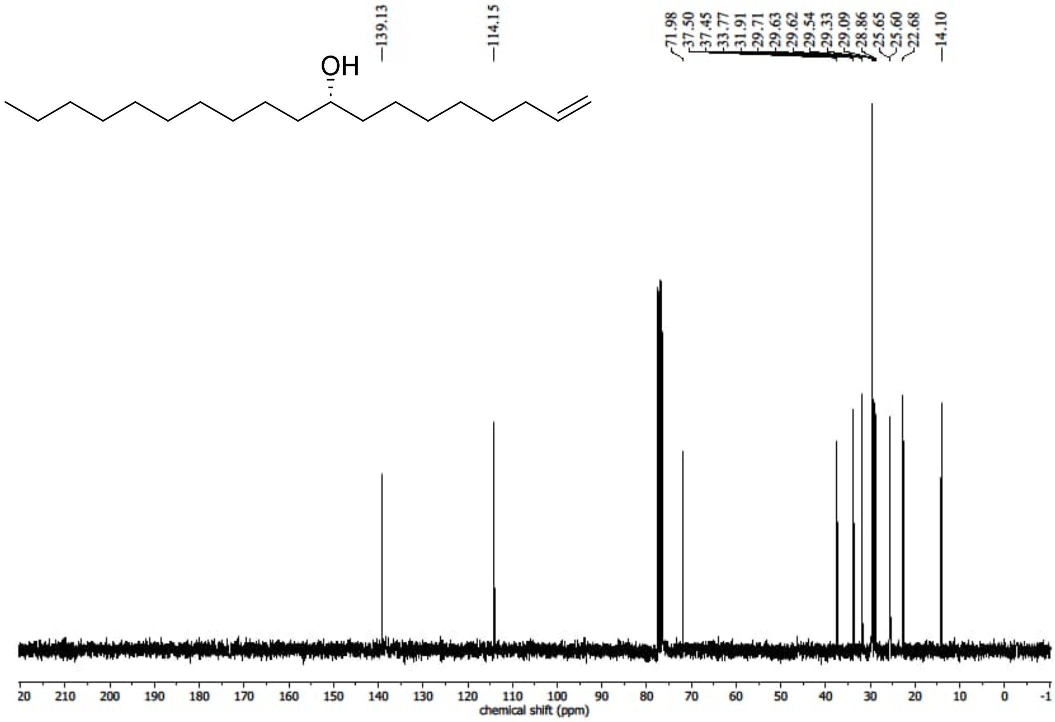

Figure S60.75 MHz ^{13}C NMR spectrum of (*S*)-nonadec-1-en-9-ol.

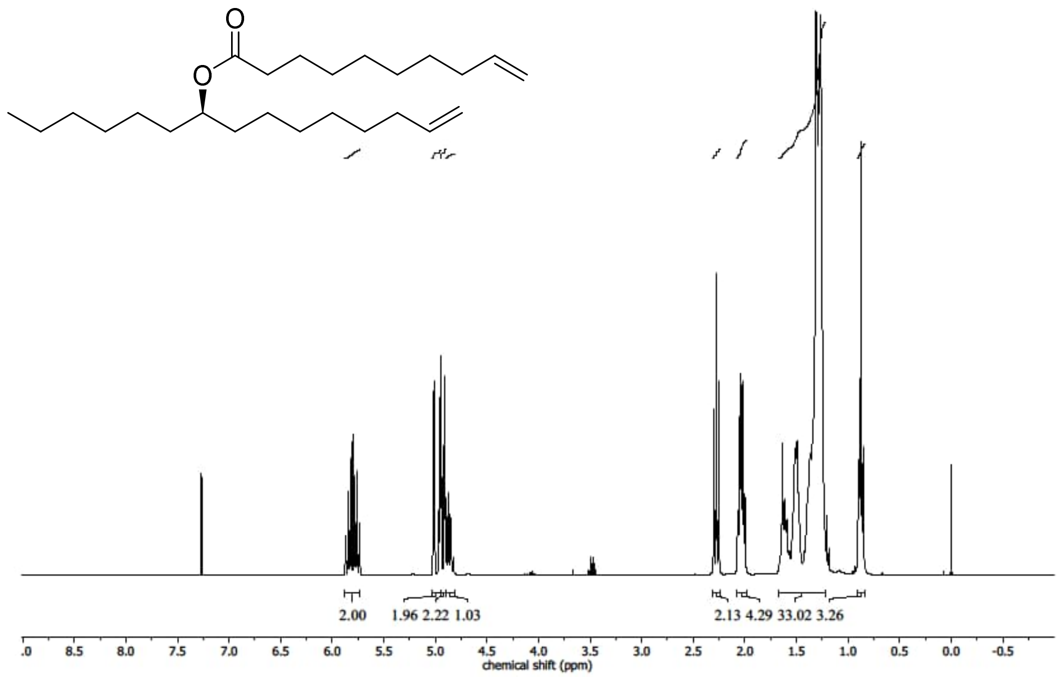

Figure S61. 300 MHz ^{1}H NMR spectrum of (*R*)-pentadec-14-en-7-yl dec-9-enoate.

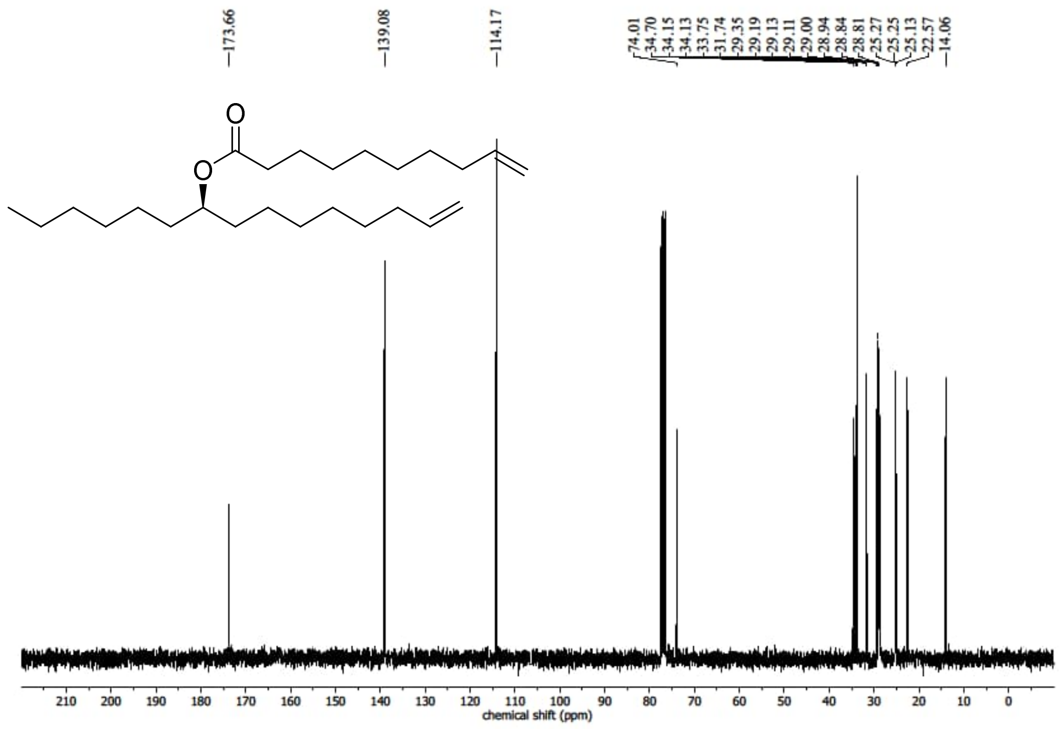

Figure S62. 75 MHz ^{13}C NMR spectrum of (*R*)-pentadec-14-en-7-yl dec-9-enoate.

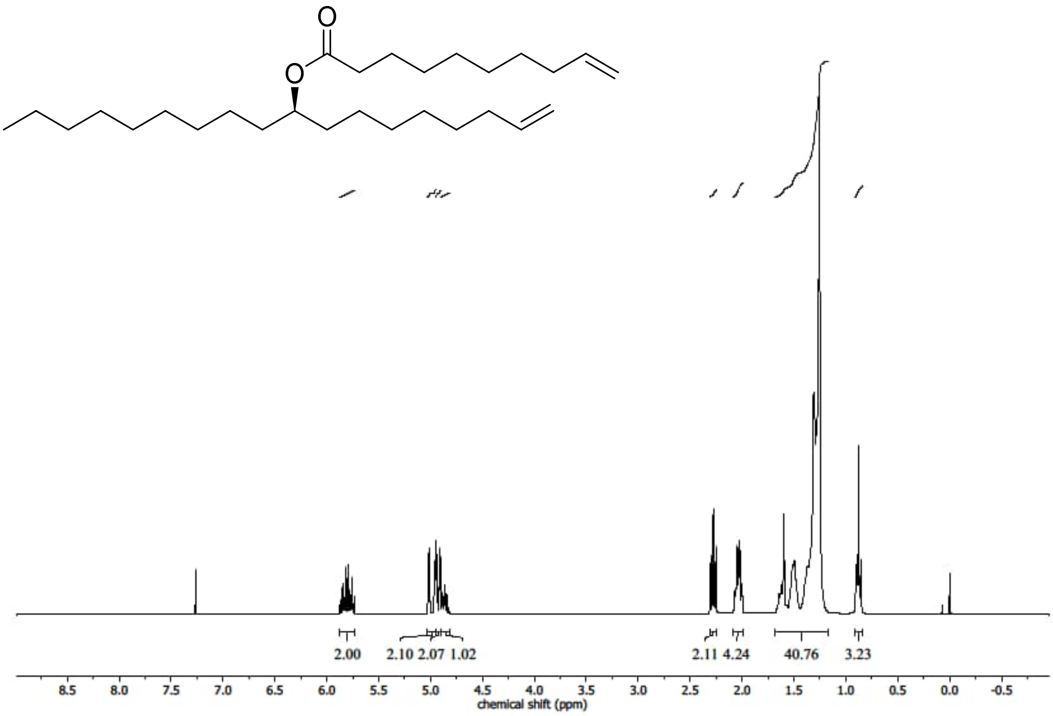

Figure S63. 300 MHz ^{1}H NMR spectrum of (*R*)-octadec-1-en-9-yl dec-9-enoate.

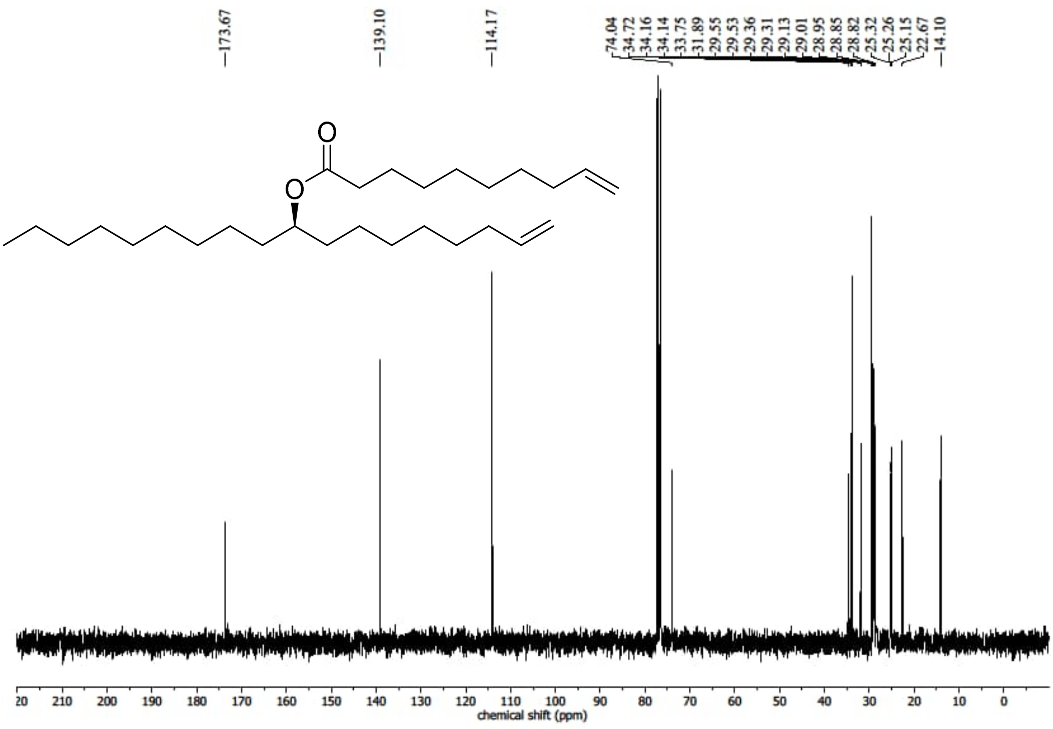

Figure S64. 75 MHz ^{13}C NMR spectrum of (*R*)-octadec-1-en-9-yl dec-9-enoate.

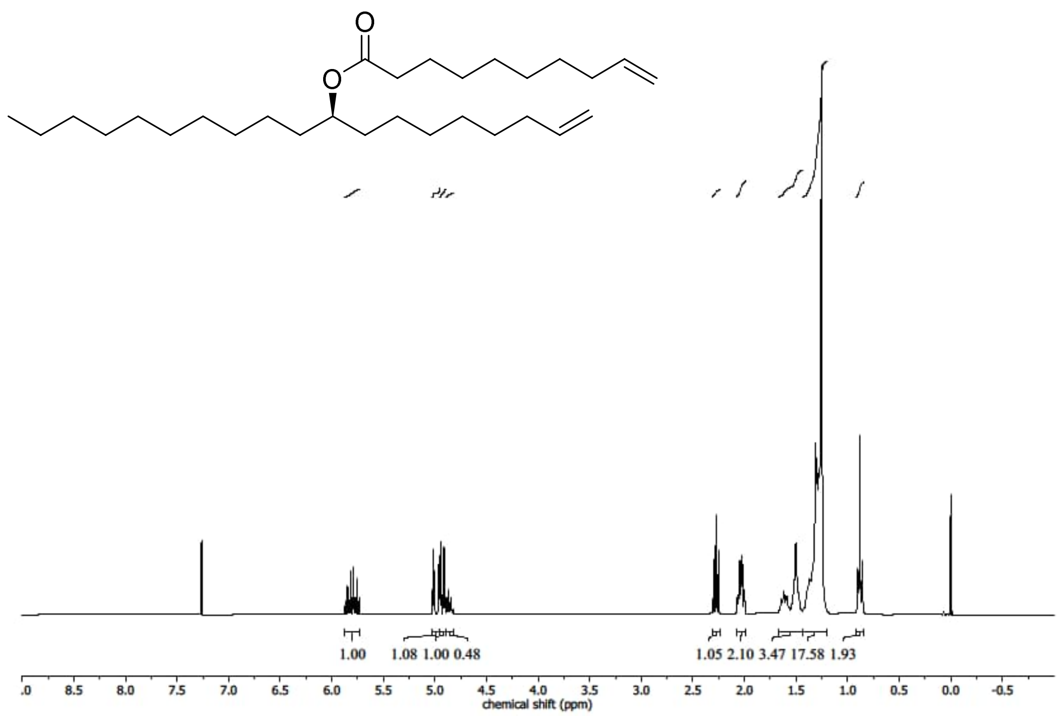

Figure S65. 300 MHz ^{1}H NMR spectrum of (*R*)-nonadec-1-en-9-yl dec-9-enoate.

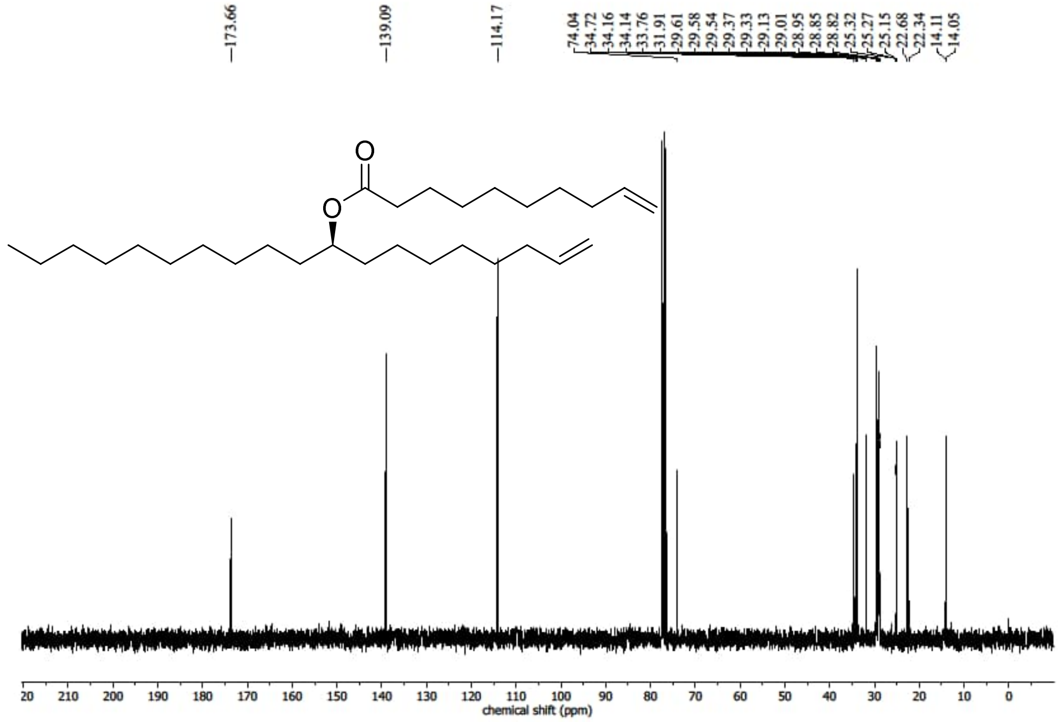

Figure S66 75 MHz ^{13}C NMR spectrum of (*R*)-nonadec-1-en-9-yl dec-9-enoate.

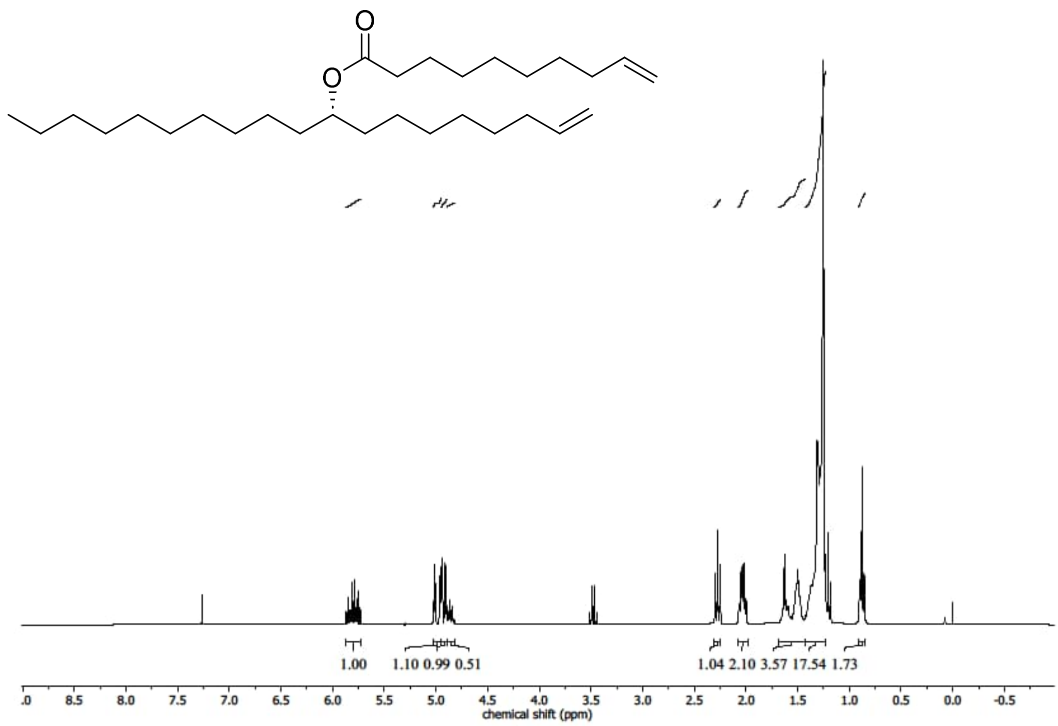

Figure S67. 300 MHz ^{1}H NMR spectrum of (*S*)-nonadec-1-en-9-yl dec-9-enoate.

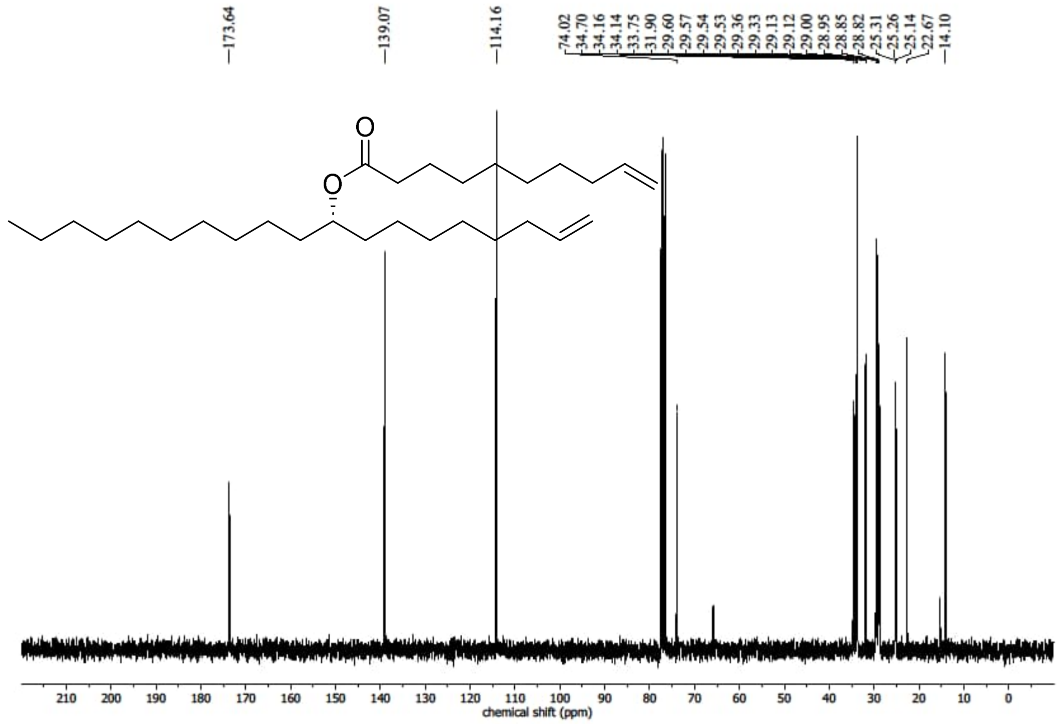

Figure S68. 75 MHz ^{13}C NMR spectrum of (*S*)-nonadec-1-en-9-yl dec-9-enoate.

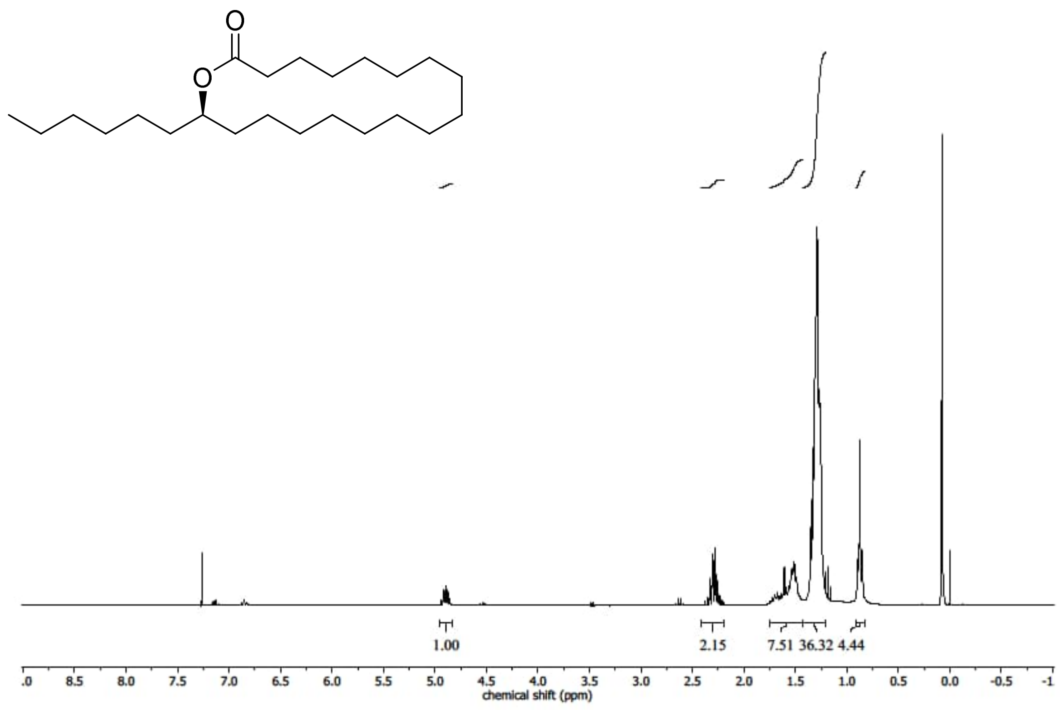

Figure S69. 300 MHz ^{1}H NMR spectrum of (*R*)-tricosan-17-olide.

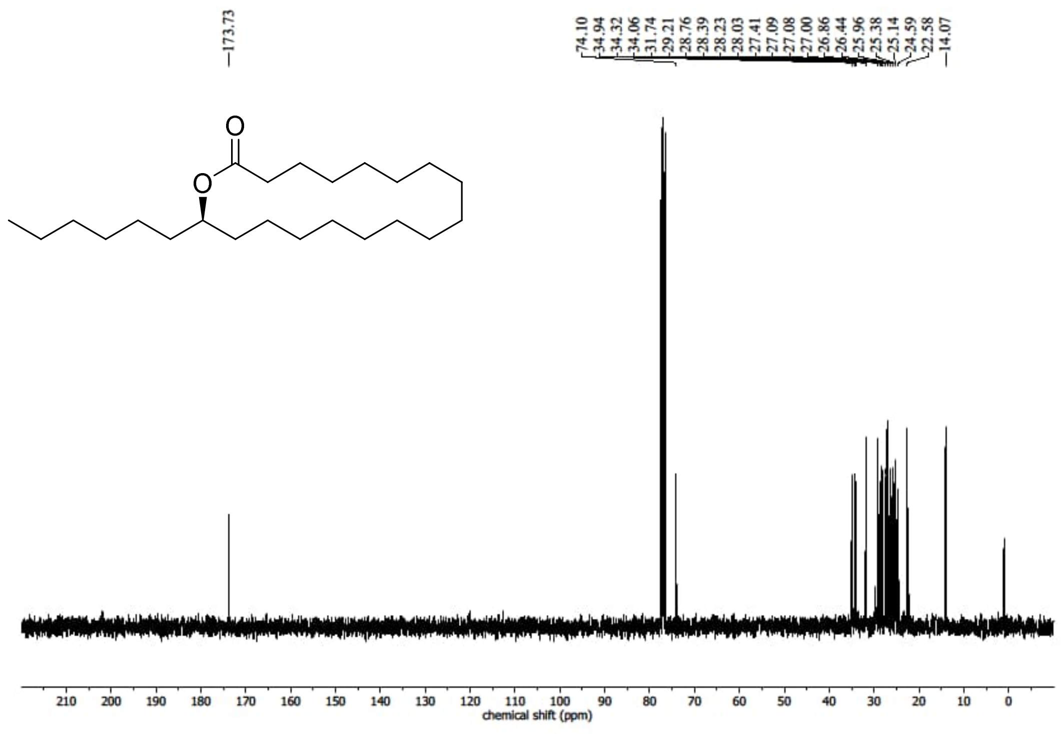

Figure S70. 75 MHz ^{13}C NMR spectrum of (*R*)-tricosan-17-olide.

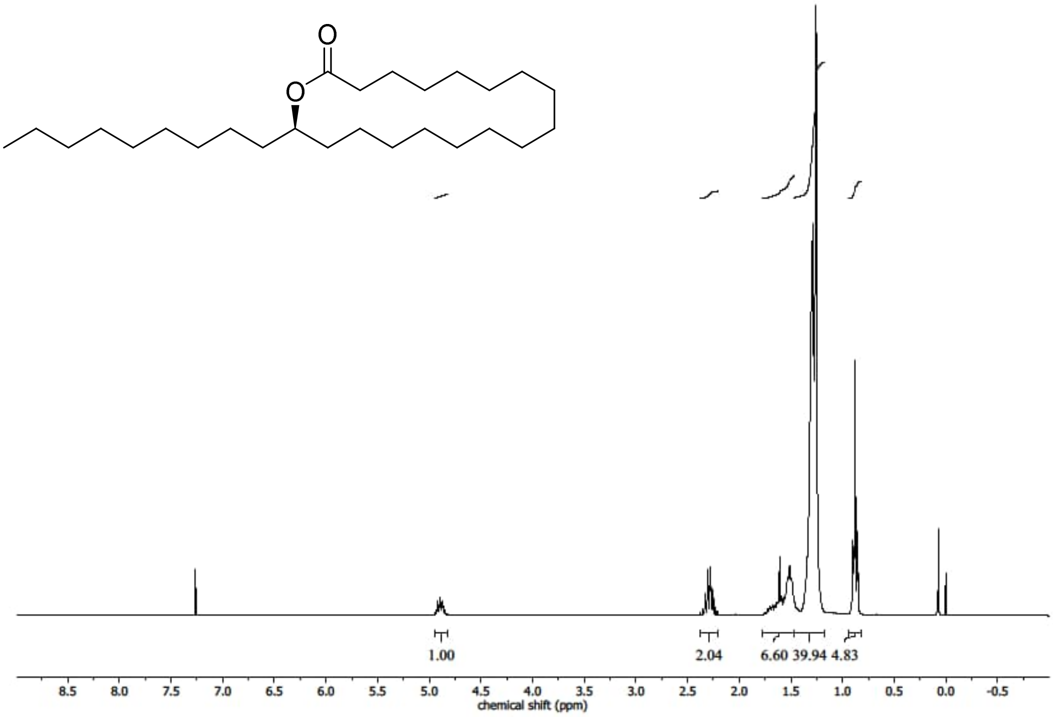

Figure S71. 300 MHz ^{1}H NMR spectrum of (*R*)-hexacosan-17-olide.

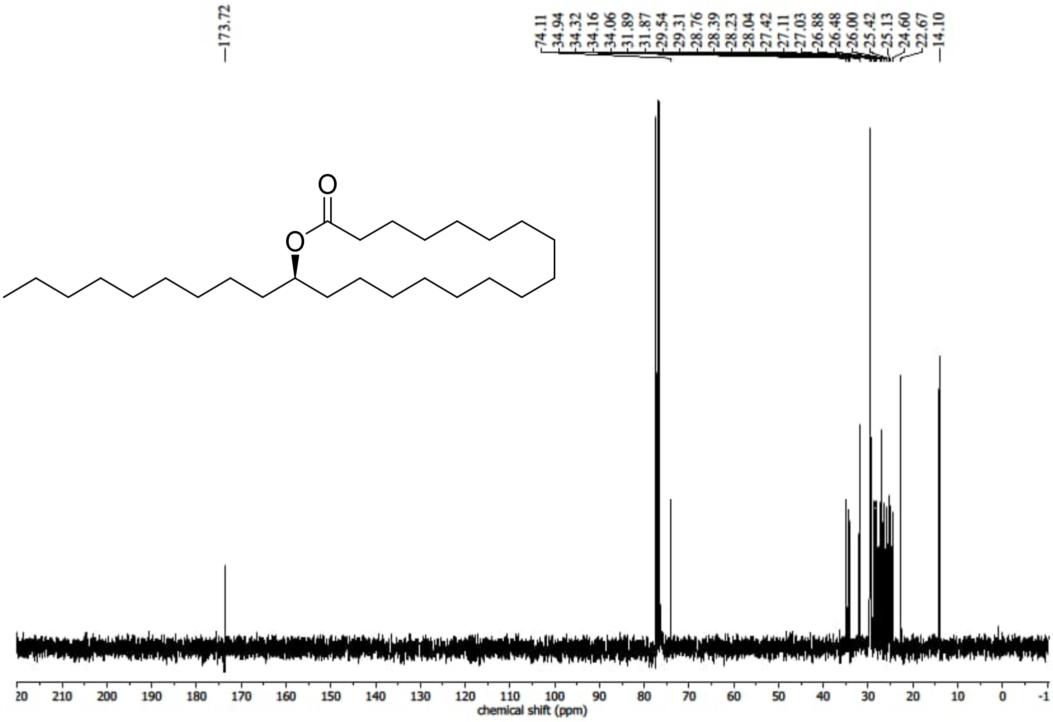

Figure S72. 75 MHz ^{13}C NMR spectrum of (*R*)-hexacosan-17-olide.

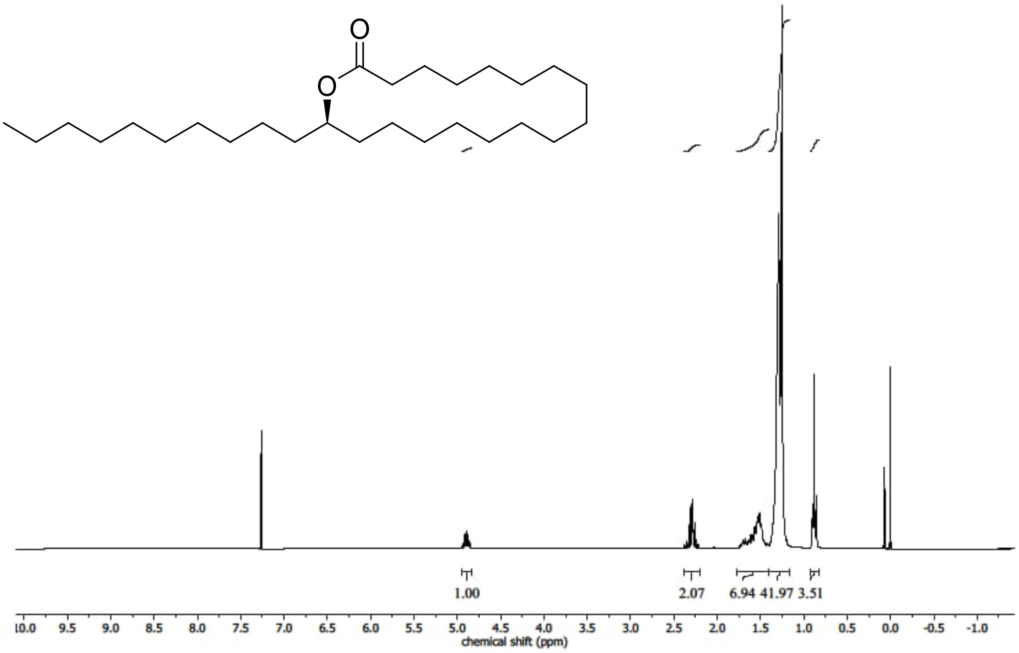

Figure S73. 300 MHz ^{1}H NMR spectrum of heptacosan-17-olide (*R*-**2**).

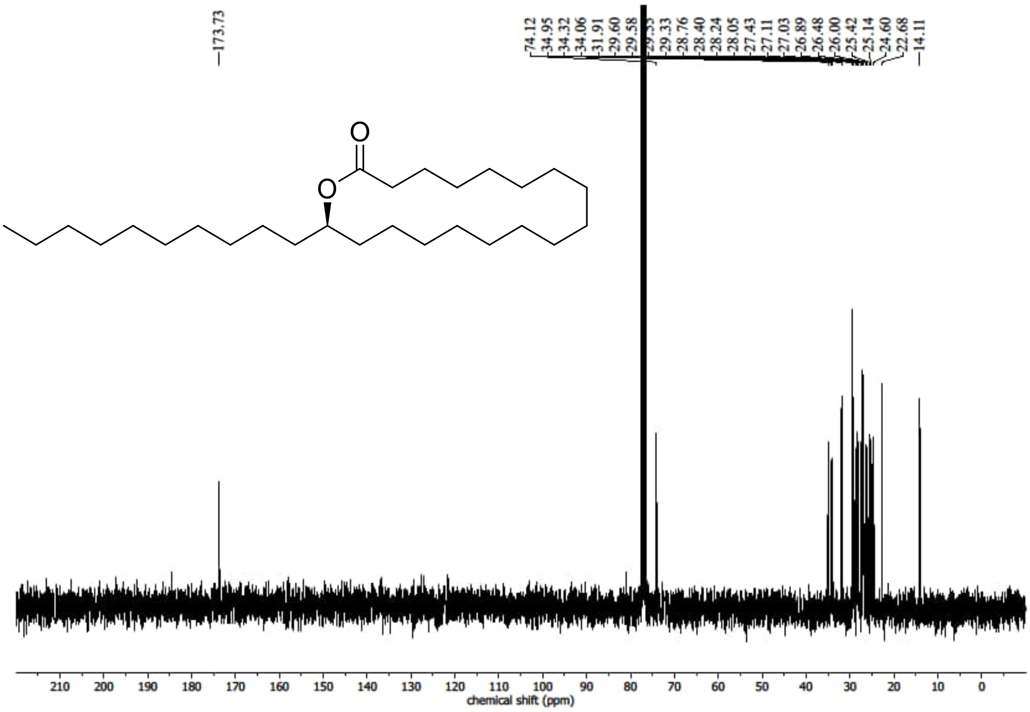

Figure S74. 75 MHz ^{13}C NMR spectrum of heptacosan-17-olide (*R*-**2**).

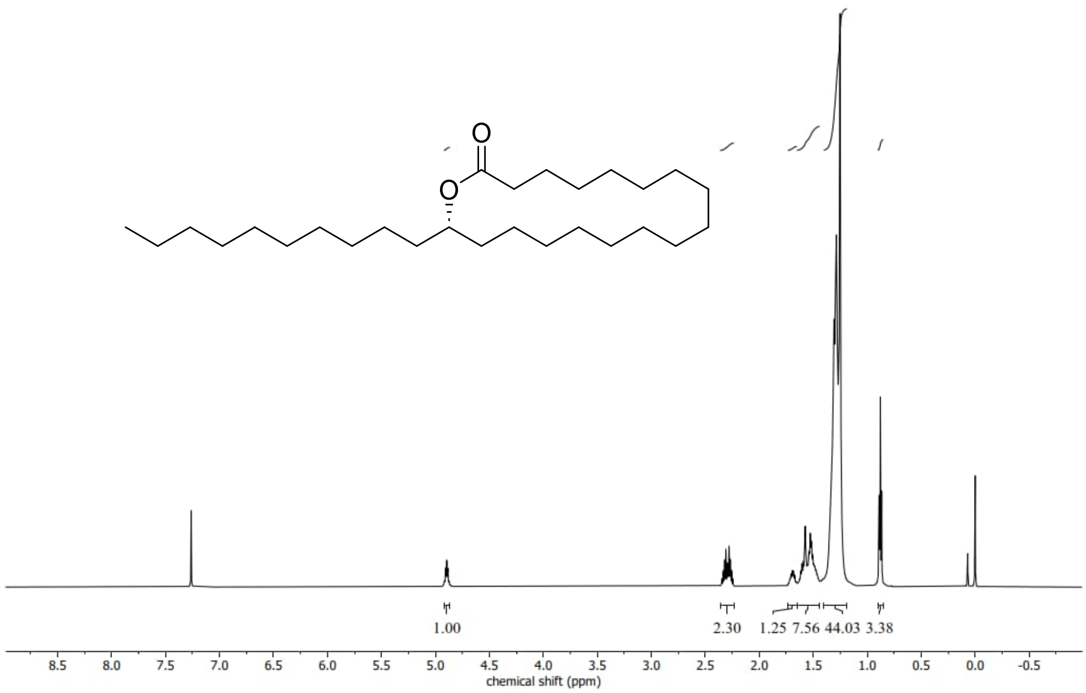

Figure S75. 600 MHz ^{1}H NMR spectrum of heptacosan-17-olide (*S*-**2**).

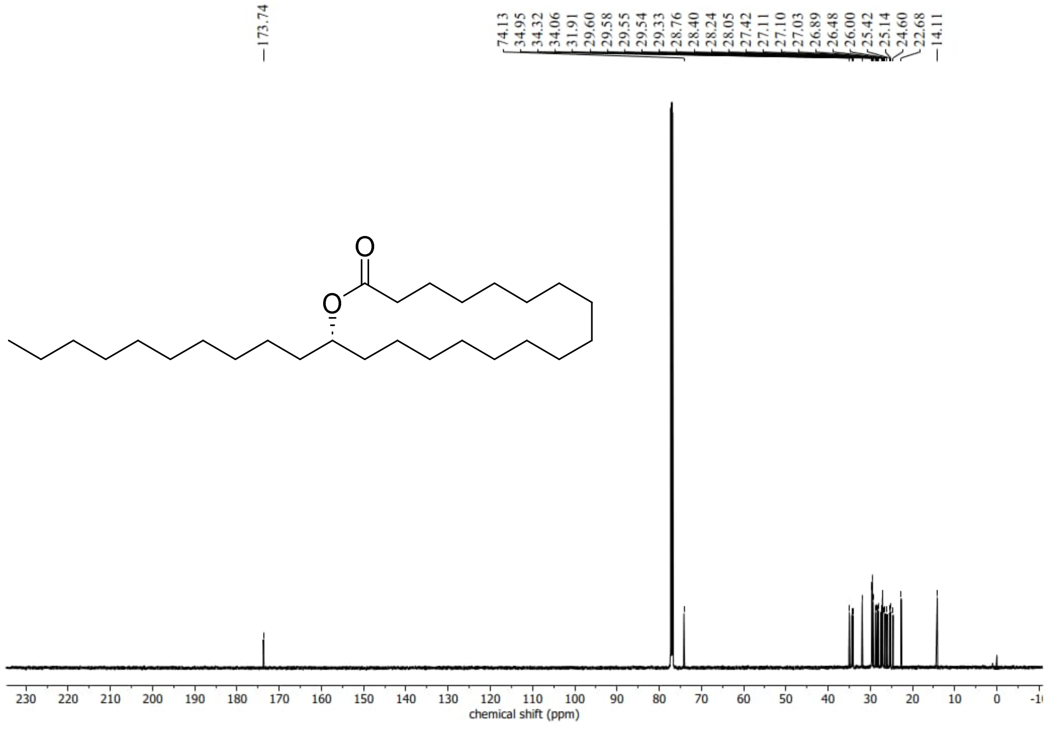

Figure S76. 150 MHz ^{13}C NMR spectrum of heptacosan-17-olide (*S*-**2**).

7 IR Spectra

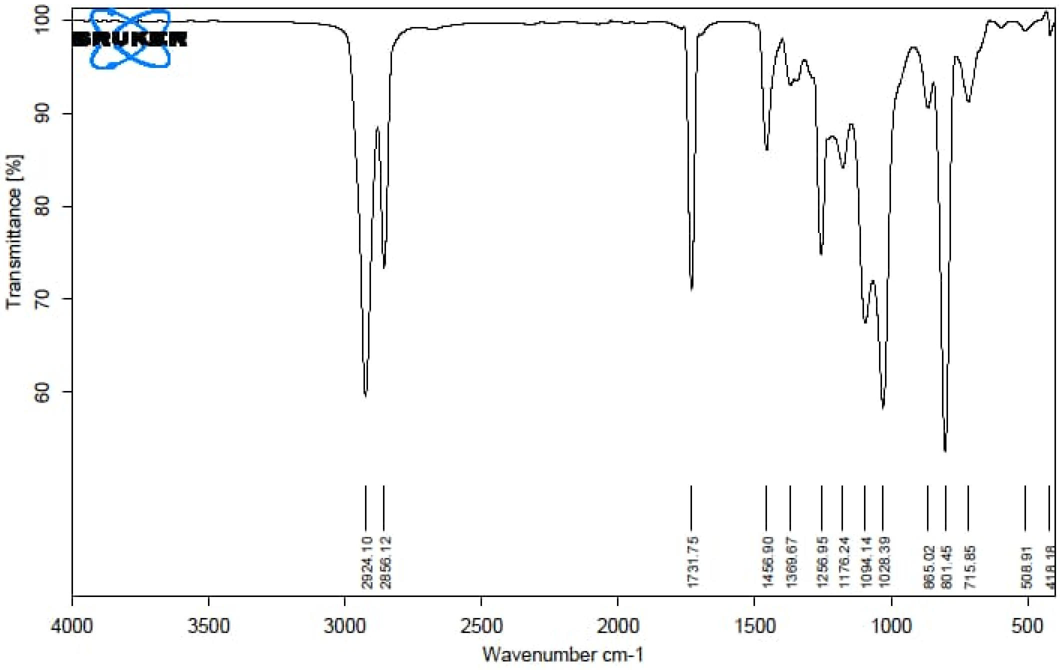

Figure S77. IR spectrum of tricosan-17-olide.

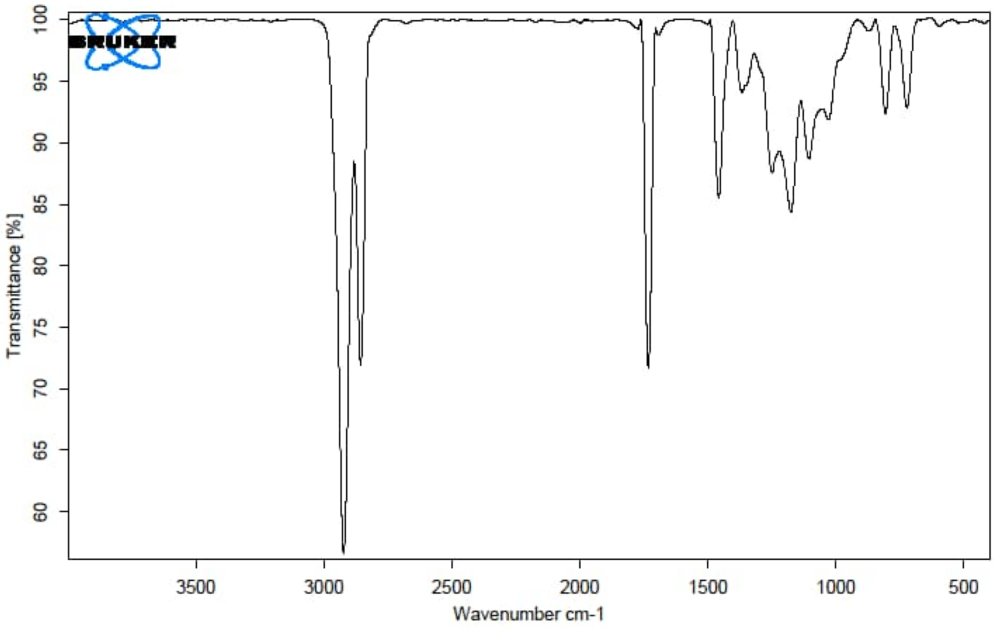

Figure S78. IR spectrum of hexacosan-17-olide.

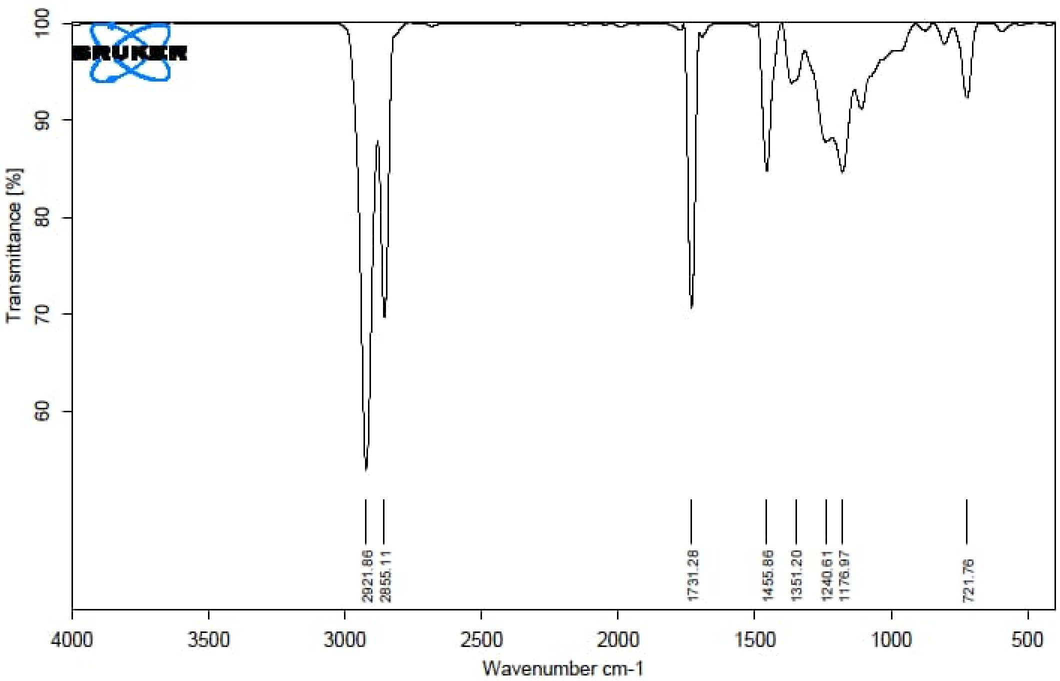

Figure S79. IR spectrum of heptacosan-17-olide (**2**).

8 References

(1) Schulz, S.; Möllerke, A. MACE, an Open Access Database of Electron Impact Mass Spectra of Natural Products. *J. Nat. Prod.* **2024**, *87*, 2567. DOI: 10.1021/acs.jnatprod.4c01240.
(2) Schulz, S.; Möllerke, A. MACE - An Open Access Data Repository of Mass Spectra for Chemical Ecology. *J. Chem. Ecol.* **2022**, *48*, 589–597. DOI: 10.1007/s10886-022-01364-4.
(3) Kozak, K. M.; Wahlberg, N.; Neild, A. F. E.; Dasmahapatra, K. K.; Mallet, J.; Jiggins, C. D. Multilocus species trees show the recent adaptive radiation of the mimetic *Heliconius* butterflies. *Syst. Biol.* **2015**, *64*, 505–524. DOI: 10.1093/sysbio/syv007.
